# Diagnostic tests for human *Schistosoma mansoni* and *Schistosoma haematobium* infection: a systematic review and meta-analysis

**DOI:** 10.1016/S2666-5247(23)00377-4

**Published:** 2024-04

**Authors:** Michel T Vaillant, Fred Philippy, Anouk Neven, Jessica Barré, Dmitry Bulaev, Piero L Olliaro, Jürg Utzinger, Jennifer Keiser, Amadou T Garba

**Affiliations:** a Competence Center for Methodology and Statistics, Luxembourg Institute of Health, Strassen, Luxembourg; b Zortify, Luxembourg City, Luxembourg; c Luxembourg National Office of Health, Luxembourg City, Luxembourg; d International Severe Acute Respiratory and Emerging Infection Consortium, Pandemic Sciences Institute, University of Oxford, Oxford, UK; e Swiss Tropical and Public Health Institute, Allschwil, Switzerland; f Swiss Tropical and Public Health Institute and Medical Parasitology and Infection Biology Department, University of Basel, Basel, Switzerland; g Department of Control of Neglected Tropical Diseases, WHO, Geneva, Switzerland

## Abstract

**Background:**

Accurate diagnosis is pivotal for implementing strategies for surveillance, control, and elimination of schistosomiasis. Despite their low sensitivity in low-endemicity areas, microscopy-based urine filtration and the Kato-Katz technique are considered as reference diagnostic tests for *Schistosoma haematobium* and *Schistosoma mansoni* infections, respectively. We aimed to collate all available evidence on the accuracy of other proposed diagnostic techniques.

**Methods:**

In this systematic review and meta-analysis, we searched PubMed, Embase, the Cochrane Library, and LILACS for studies published from database inception to Dec 31, 2022, investigating the sensitivity and specificity of diagnostic tests for *S haematobium* and *S mansoni* infections against Kato-Katz thick smears or urine microscopy (reference tests) involving adults (aged ≥18 years), school-aged children (aged 7 to 18 years), or preschool-aged children (aged 1 month to 7 years). We extracted raw data on true positives, true negatives, false positives, and false negatives for the diagnostic tests and data on the number of participants, study authors, publication year, journal, study design, participants’ age and sex, prevalence of *Schistosoma* infection, and treatment status. To account for imperfect reference tests, we used a hierarchical Bayesian latent class meta-analysis to model test accuracy.

**Findings:**

Overall, we included 121 studies, assessing 28 different diagnostic techniques. Most studies (103 [85%] of 121) were done in Africa, 14 (12%) in South America, one (1%) in Asia, and one (1%) in an unknown country. Compared with the reference test, Kato-Katz thick smears, circulating cathodic antigen urine cassette assay version 1 (CCA1, 36 test comparisons) had excellent sensitivity (95% [95% credible interval 88–99]) and reasonable specificity (74% [63–83]) for *S mansoni*. ELISA-based tests had a performance comparable to circulating cathodic antigen, but there were few available test comparisons. For *S haematobium*, proteinuria (42 test comparisons, sensitivity 73% [62–82]; specificity 94% [89–98]) and haematuria (75 test comparisons, sensitivity 85% [80–90]; specificity 96% [92–99]) reagent strips showed high specificity, with haematuria reagent strips having better sensitivity. Despite limited data, nucleic acid amplification tests (NAATs; eg, PCR or loop-mediated isothermal amplification [LAMP]) showed promising results with sensitivity estimates above 90%. We found an unclear risk of bias of about 70% in the use of the reference or index tests and of 50% in patient selection. All analyses showed substantial heterogeneity (*I*^2^>80%).

**Interpretation:**

Although NAATs and immunological diagnostics show promise, the limited information available precludes drawing definitive conclusions. Additional research on diagnostic accuracy and cost-effectiveness is needed before the replacement of conventional tests can be considered.

**Funding:**

WHO and Luxembourg Institute of Health.

## Introduction

Schistosomiasis is a parasitic disease caused by blood flukes of the genus *Schistosoma.* The disease has an acute phase that is followed by a chronic phase due to repeated exposure to the parasite if untreated.^1^, ^2^, ^3^ The main species causing human infections are *Schistosoma mansoni* and *Schistosoma haematobium*. Approximately 779 million people are at risk of infection.^4^ In 2021, an estimated 136 million school-aged children (aged 7–18 years) and 115 million adults (≥18 years) worldwide needed preventive chemotherapy in 51 countries. However, only 8·9 million school-aged children and 16·4 million adults were treated.^4^ A national coverage of more than 75% was achieved in 16 of 37 countries. The African region has the highest disease burden with 41 of 51 countries affected and with 90·6% of people infected requiring preventive chemotherapy.^4^

**Research in context**

**Evidence before this study**

Accurate tests for the diagnosis of human *Schistosoma* infections are crucial in implementing strategies for the surveillance, control, and elimination of schistosomiasis. The conventional reference diagnostic test for *Schistosoma mansoni* is a duplicate Kato-Katz thick smear, done on stool samples and subsequent microscopic readout. The conventional reference diagnostic test for *Schistosoma haematobium* is urine filtration and microscopy. However, both tests underestimate the prevalence of infection due to low sensitivity, especially in areas where the intensity of infection is low. New diagnostic methods have emerged since the 2017 update of WHO's guidelines on control and elimination of schistosomiasis using antigen-based assays or nucleic acid amplification tests (NAATs). Several systematic reviews and meta-analyses on diagnostic techniques for *Schistosoma* spp have been done, but they all either focused on a specific diagnostic type (eg, molecular-based assays) or did not account in their analyses for the imperfect nature (sensitivity <100%) of the reference tests.

**Added value of this study**

Our meta-analysis summarises the current literature of all available diagnostic assays up to Dec 31, 2022, using as reference standard the Kato-Katz test for *S mansoni* and urine filtration for *S haematobium*. The innovative aspect of the current work lies in the modelling of the diagnostic accuracy of tests through a hierarchical Bayesian latent-class model to account for imperfect reference tests. Our analyses suggest that the circulating cathodic antigen (CCA) test for *S mansoni* detects a large proportion of infections identified by the Kato-Katz test (sensitivity 95%) and has reasonable specificity (74%) based on 36 direct comparisons. Confidence in the results of other diagnostic tests is limited, due to small number of studies and sample size. NAATs provided promising preliminary results. For the detection of *S haematobium*, our results suggest that haematuria reagent strips detect the largest proportion of infections (sensitivity 85%) and non-infections (specificity 96%) identified by microscopy. Proteinuria reagent strips have a similar specificity (94%) but a lower sensitivity (73%), whereas leukocyturia reagent strips have lower sensitivity and specificity.

**Implications of all the available evidence**

The use of sensitive and specific diagnostic tools is pivotal for accurate surveillance and for supporting the move towards the elimination of schistosomiasis. New alternative diagnostic tools show promise but have insufficient data on diagnostic accuracy. Although conventional methods are well accepted, cheap, and feasible, new tools often lack commercially ready forms and might require specific laboratory facilities and dedicated resources. More research, including on diagnostic accuracy in low-prevalence areas and on the cost-effectiveness of new assays, is needed to identify a suitable replacement for the standard references.

The main WHO elimination objective translates into eliminating the morbidity associated with the disease in the target population by reducing the prevalence of moderate-intensity and heavy-intensity infections and the overall prevalence of infection, mainly by preventive chemotherapy with praziquantel.^4^ However, the main hurdles to this strategy are the diagnostic tests used to detect the presence of the parasite, which are inaccurate in terms of sensitivity, especially in low-prevalence settings. For *S mansoni*, the conventional reference standard diagnostic test is duplicate Kato-Katz thick smears and the main method for detecting infection with *S haematobium* is urine filtration and microscopy. These methods have been available for decades and are widely implemented because of their simplicity and low cost; however, their sensitivity is low.

To inform policy recommendations by the WHO expert group on diagnostic tools for human *Schistosoma* infections in the context of verification of transmission interruption, we performed a systematic review and meta-analysis. Originally published on MedRxiv on May 9, 2021,^5^ we have updated this analysis to include articles published until Dec 31, 2022. The objectives of the current work were to assess and compare the sensitivity and specificity of a wide range of diagnostic tools, using as the reference standards Kato-Katz thick smears for *S mansoni* and urine filtration for *S haematobium* (appendix 1 pp 2–6). For *Schistosoma japonicum* and *Schistosoma mekongi* species, a systematic review and meta-analysis was published in 2021.^6^

## Methods

### Search strategy and selection criteria

In this systematic review and meta-analysis, we searched PubMed, Embase, the Cochrane Library, and LILACS from database inception to Dec 31, 2022 (the initial search was from database inception to Feb 28, 2021) using a broad search strategy. Keywords included (“schistosomiasis” OR “*Schistosoma*”) AND (“diagnostic” OR “sensitivity” OR “specificity”). Details of the search strategy are presented in appendix 1 (p 3). Citations from relevant articles were hand-searched to identify potential additional studies. We included all studies published in English or French that involved adults (aged ≥18 years), school-aged children (aged 7 to 18 years), or preschool-aged children (aged 1 month to 7 years) living in schistosomiasis-endemic areas and being investigated for *S mansoni* or *S haematobium* infection. Acceptable primary research studies were studies in humans investigating the use of diagnostic tests, validation studies, and studies with test performance measurements available in crossed categories of at least two diagnostic tests (confusion matrix or sensitivity and specificity) and using Kato-Katz thick smears or urine microscopy as the reference standard. We excluded articles if they did not report or provided insufficient data to obtain sensitivity and specificity; were animal or in-vitro studies; or used a composite gold standard. We only used data included in the articles and we did not contact the authors of studies included. All search results were uploaded in Rayyan QRCI^7^ for sorting and selection of articles by two review authors (FP and DB for the initial search, AN and DB for the updated search). Studies were independently reviewed by title, then by abstract, and, finally, by full-text review. We resolved uncertainties regarding the inclusion or exclusion of studies by consensus.

The systematic review of the literature and the diagnostic test accuracy meta-analysis were conducted in accordance with PRISMA guidelines.^8^ The study was not registered in PROSPERO at the time of inception.

### Data analysis

For studies selected after full-text review, items for data extraction were predefined and agreed on by all authors, with any uncertainty resolved by consensus. We extracted raw data on true positives, true negatives, false positives, and false negatives for the diagnostic tests. Other extracted variables were study authors, publication year, journal, study design, participants’ age and sex, prevalence of *Schistosoma* infection, praziquantel treatment status of participants before the study start, reference standard (urine filtration and Kato-Katz thick smears), and index tests.

Quality assessment was done using the QUADAS-2 guidelines for the review of diagnostic accuracy.^9^ We assessed risk of bias and applicability concerns using four domains: patient selection, index test, reference standard, and patient flow and timing of tests. We reported the level of risk or concern as either high, low, or unclear as per the Cochrane handbook assessment guideline.^10^ We also used the Grading of Recommendations, Assessment, Development, and Evaluation (GRADE) methodology^11^^,^^12^ to rate the quality of evidence and strength of recommendation for each diagnostic tool. Information was gathered through the GRADEpro guideline development tool.^13^

We used forest plots to display sensitivity and specificity estimates for each study (with 95% CIs) against Kato-Katz thick smears for *S mansoni* and urine filtration for *S haematobium*, stratified by test comparison. When data from at least two studies were available, we derived estimates of the pooled sensitivity and specificity with their 95% credible intervals (CrIs) for the two diagnostic tests under comparison using a Bayesian bivariate random effect model with non-informative priors.^14^ We also plotted the hierarchical summary receiver operating characteristic (HSROC) curves.^15^

To acknowledge that the reference standards for schistosomiasis are imperfect tests with low sensitivity, especially when applied in regions of low endemicity,^16^^,^^17^ we conducted a hierarchical Bayesian latent-class meta-analysis under conditional independence assumption as a second step.^18^, ^19^, ^20^ Statistical models and methodology are further detailed in appendix 1 (pp 4–6).

For diagnostic assay comparisons available in at least ten studies, we did a sensitivity analysis incorporating conditional dependence.^21^ For every test comparison described in more than four studies, we investigated publication bias using Deeks funnel plots and asymmetry tests and heterogeneity across studies with forest plots and the *I*^2^ statistic.^22^ Statistical analyses were done using the Review Manager V5, SAS System (9.4), R Studio (2022.07.1), and R (4.2.3).

### Role of the funding source

WHO commissioned this review and meta-analysis to inform its policy statement and received an early version of the manuscript in the form of an official report. The search update from Feb 28, 2021, to Dec 31, 2022, was done on the authors’ own resources. The funders had no role in data collection and analysis but participated in the interpretation and writing of the manuscript (ATG).

## Results

**Figure 1:**
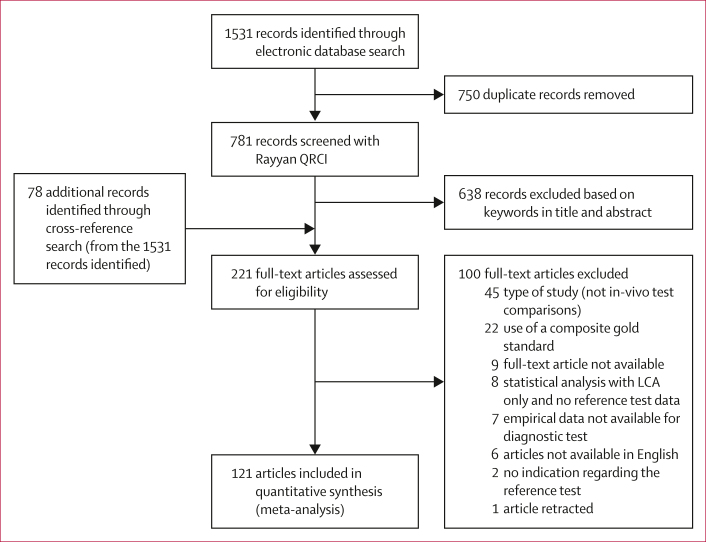
Study selection LCA=latent-class analysis.

We identified 1531 records from our electronic search, and, after removing duplicates, we identified 78 records through hand-searches of the reference lists of these articles, resulting in a total of 221 records qualifying for full-text assessment. After full-text review, 121 records were eligible for inclusion (figure 1; appendix 1 pp 7–13; appendix 2 pp 1–3).

The studies included were published between March 28, 1979, and Dec 31, 2022, and all except nine studies used a cross-sectional design. Two studies were longitudinal surveys,^23^^,^^24^ two were nested case-control studies,^25^^,^^26^ and five were based on a cohort design.^27^, ^28^, ^29^, ^30^, ^31^ 103 (85%) of 121 studies were done in Africa, 14 (12%) in South America (13 in Brazil, one in Surinam), one (1%) in Asia (Yemen), and the country was unknown for one study (1%; appendix 2 pp 4–9).^32^ The remaining two studies (2%) recruited African immigrants in Europe.^33^^,^^34^ Most studies did not provide data on praziquantel treatment and only five studies indicated a history of treatment with praziquantel.^27^^,^^35^, ^36^, ^37^, ^38^

The overall risk of bias is summarised graphically in appendix 1 (pp 14–17). In general, poor reporting of quality items (eg, patient selection, index and reference test description and use, flow and timing) hindered our evaluation of quality. Following the Cochrane handbook, when insufficient data were available to make a judgement, the review authors evaluated an unclear risk of bias of about 70% in the use of the reference or index tests and of 50% in patient selection. Concerns for applicability were predominantly low.

We identified 28 different techniques (not including reference tests) for diagnosis of *S mansoni* and *S haematobium* infections in humans (table 1). For 14 diagnostic tests, data from at least two studies were available for at least one *Schistosoma* species, which allowed the computation of meta-analytic estimates. The other 14 tests were investigated in only one study (appendix 1 pp 18–19).

**Table 1:** Diagnostic tests for *Schistosoma mansoni* and *Schistosoma haematobium* infection identified through the search of articles

	Target	Technique
***S mansoni***		
Single Kato-Katz thick smears∗	Eggs	Microscopy
Duplicate Kato-Katz thick smears∗	Eggs	Microscopy
Quadruple Kato-Katz thick smears∗	Eggs	Microscopy
Sextuple Kato-Katz thick smears∗	Eggs	Microscopy
Triplicate Kato-Katz thick smears∗	Eggs	Microscopy
16 Kato-Katz thick smears∗	Eggs	Microscopy
18 Kato-Katz thick smears∗	Eggs	Microscopy
27 Kato-Katz thick smears∗	Eggs	Microscopy
CCA1†	Cathodic antigen	Immunochromatographic test
CCA2	Cathodic antigen	Immunochromatographic test
CAA†	Anodic antigen	Lateral flow test
AWE-SEA ELISA†	Adult worm extract and soluble egg antigen	Indirect haemagglutination ELISA
Helmintex‡	Eggs	Magnetic field
SmCTF-RDT†	Cercarial transformation fluid	point-of-care-RDT
*S mansoni* DNA PCR	DNA	PCR
IgM ELISA§	Adult worm antigen	ELISA
IgG ELISA†,¶	Adult worm antigen	ELISA
PCR†	DNA	PCR
rtPCR†	DNA	PCR
LAMP†	DNA	LAMP
SWAP Elisa†,||	Adult worm antigen	ELISA
PCR-ELISA platform version 1	DNA	PCR-ELISA
PCR-ELISA platform version 2	DNA	PCR-ELISA
COPT	Eggs	COPT
***S haematobium***		
Urine filtration∗	Eggs	Microscopy
Proteinuria reagent strips†	Urinary proteins	Reagent strips
Haematuria reagent strips†	Urinary red cells	Reagent strips
Leukocyturia reagent strips†	Urinary white cells	Reagent strips
FLOTAC	Eggs	Microscopy
LAMP†	DNA	LAMP
IgG SEA ELISA†	Soluble egg antigen	ELISA
Immunomagnetic beads-based ELISA	Circulating schistosomal antigen	ELISA
Anti-IgG RDT *S haematobium*	Eggs	RDT
IHA	Adult worm antigen	IHA
Macroscopic haematuria by colorimetric test	Urinary red cells	Colorimetric test
DDIA	Soluble egg antigen	Dipstick
Schistoscope††	Eggs	Microscopy

AWE=*S mansoni* adult worm extract. CAA=circulating anodic antigen serum and urine assay. CCA1=circulating cathodic antigen urine cassette assay version 1. CCA2=circulating cathodic antigen urine cassette assay version 2. COPT=circumoval precipitin test. DDIA=dipstick dye immunoassay. FLOTAC=centrifugal flotation of a faecal sample suspension. IHA=indirect haemagglutination assay. LAMP=loop-mediated isothermal amplification. RDT=rapid diagnostic test. rtPCR=real-time PCR. SEA=*S mansoni* soluble egg antigen. SmCTF=*S mansoni* cercarial transformation fluid. SWAP=soluble adult worm antigen preparation.

∗ Reference tests.

† Diagnostic tests were investigated in at least two studies.

‡ Isolation of eggs from faecal samples by using paramagnetic particles in a magnetic field.

§ IgM antibodies against a fraction of *S mansoni* adult worm antigen.

¶ IgG antibodies against a fraction of *S mansoni* adult worm antigen.

|| SWAP-specific IgG ELISA.

†† Mobile phone microscopy.

**Figure 2:**
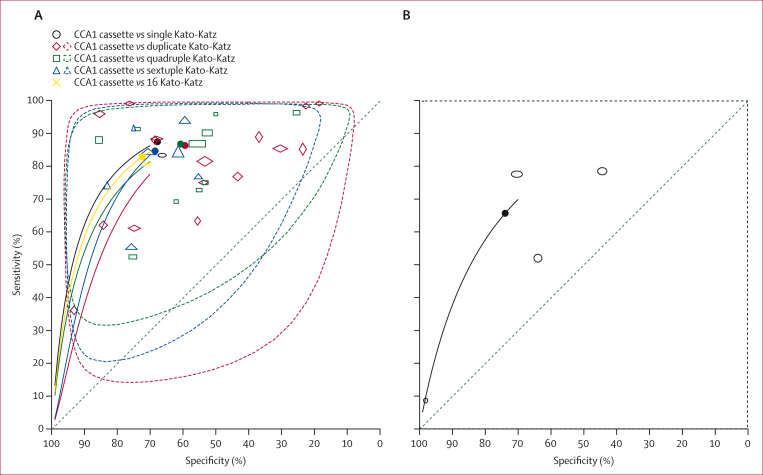
HSROC plot with summary points (A) CCA1 tests versus single, double, quadruple, sextuple, or 16 Kato-Katz thick smears. (B) CCA1 tests versus urine filtration. Each unfilled symbol represents the individual study estimates pair (sensitivity, specificity), and larger symbols reflect a larger sample size. The filled circles are the meta-analytic estimates across the studies included in the meta-analysis. The solid lines are the summary HSROC curves and the dashed curves in panel A (green, blue, red) are the 95% prediction regions for a future study. The 95% prediction region in panel B is the whole plot area due to heterogeneity and few number of studies. CCA1=circulating cathodic antigen urine cassette assay version 1. HSROC=hierarchical summary receiver operating characteristic.

For *S mansoni*, the circulating cathodic antigen urine cassette assay version 1 (CCA1) test was investigated in 24 studies, reported in 23 articles with Kato-Katz technique as the reference test,^23^^,^^33^^,^^39^, ^40^, ^41^, ^42^, ^43^, ^44^, ^45^, ^46^, ^47^, ^48^, ^49^, ^50^, ^51^, ^52^, ^53^, ^54^, ^55^, ^56^, ^57^, ^58^, ^59^ totalling 36 comparisons against single (one comparison), duplicate (17 comparisons), quadruple (ten comparisons), sextuple (seven comparisons), or 16 Kato-Katz thick smears (one comparison). Prevalence ranged from 5·5% to 94·7% and the point estimates ranged from 36% to 99% for sensitivity and from 19% to 93% for specificity across all of the CCA1 test comparisons (appendix 1 pp 19–20; appendix 2 p 10–15). The meta-analysis results, stratified by test comparison, assuming both a perfect and imperfect reference test are presented in table 2. Using Kato-Katz thick smears (different thick smears lumped together as reference test), the latent-class model estimated that the pooled sensitivity of CCA1 tests was 95% (95% CrI 88–99) and that the pooled specificity was 74% (63–83). A total of four studies^60^, ^61^, ^62^, ^63^ evaluated CCA1 tests against urine filtration for detection of *S haematobium*. The pooled sensitivity estimate was 68% (95% CrI 29–95) and the pooled specificity estimate was 81% (47–98; table 2). All analyses showed substantial heterogeneity (*I*^2^>80%, appendix 1 pp 35–36). The HSROC plots are displayed in figure 2, which confirm the heterogeneity given the large prediction limits. The sensitivity analysis allowing for conditional dependence did not significantly alter the estimates (appendix 1 p 21).

**Table 2:** Pooled sensitivity and specificity of *Schistosoma mansoni* and *Schistosoma haematobium* diagnostic tests

	Test comparisons	Participants	Perfect reference test assumption		Imperfect reference test assumption	
			Specificity	Sensitivity	Specificity	Sensitivity
***S mansoni***						
CCA1 *vs* duplicate Kato-Katz	17	4884	59% (45–72)	85% (74–93)	76% (60–88)	95% (88–99)
CCA1 *vs* quadruple Kato-Katz	10	4215	60% (45–74)	86% (76–93)	74% (59–88)	96% (90–99)
CCA1 *vs* sextuple Kato-Katz	7	2325	68% (55–79)	83% (71–92)	78% (62–95)	92% (76–99)
CCA1 *vs* Kato-Katz (all)	36	11 858	61% (51–70)	87% (81–91)	74% (63–83)	95% (88–99)
CAA *vs* duplicate Kato-Katz	3	830	67% (3–100)	61% (02–99)	95% (91–98)∗	90% (86–93)∗
SmCTF-RDT *vs* quadruple Kato-Katz	4	291	35% (14–63)	86% (30–100)	39% (11–70)	83% (33–99)
IgG ELISA *vs* triplicate Kato-Katz	3	844	80% (12–100)	95% (83–99)	98% (96–100)∗	99% (97–100)∗
AWE-SEA Elisa *vs* Kato-Katz	2	147	68% (19–97)	88% (44–100)	68% (57–79)∗	93% (81–100)∗
SWAP Elisa *vs* Kato-Katz	2	522	70% (12–99)	89% (53–99)	69% (61–80)∗	97% (92–100)∗
PCR *vs* Kato-Katz	5	652	80% (50–95)	92% (48–100)	87% (62–99)	97% (84–100)
rtPCR *vs* Kato-Katz	5	811	72% (4–100)	95% (86–99)	79% (20–100)	92% (17–100)
LAMP *vs* Kato-Katz	2	545	86% (1–100)	93% (62–100)	90% (86–93)∗	99% (96–100)∗
***S haematobium***						
CCA1 *vs* urine filtration	4	991	74% (28–97)	51% (11–90)	81% (47–98)	68% (29–95)
CAA *vs* urine filtration	4	1247	79% (14–100)	71% (20–98)	83% (25–100)	81% (24–99)
Proteinuria *vs* urine filtration	42	79 756	82% (75–88)	59% (50–67)	94% (89–98)	73% (62–82)
Haematuria *vs* urine filtration	75	174 199	87% (84–90)	75% (70–80)	96% (92–99)	85% (80–90)
Leukocyturia *vs* urine filtration	5	1532	60% (25–88)	56% (34–76)	69% (27–96)	60% (38–81)
IgG SEA ELISA *vs* urine filtration	5	793	77% (43–96)	89% (77–96)	88% (47–100)	94% (72–100)
LAMP *vs* urine filtration	3	335	64% (20–94)	86% (46–100)	92% (80–100)∗	93% (87–99)∗

Data are n, sensitivity (95% credible interval), or specificity (95% credible interval). AWE=*S mansoni* adult worm extract. CAA=circulating anodic antigen serum and urine assay. CCA1=circulating cathodic antigen urine cassette assay version 1. CCA2=circulating cathodic antigen urine cassette assay version 2. LAMP=loop-mediated isothermal amplification. RDT=rapid diagnostic test. rtPCR=real-time PCR. SEA=*S mansoni* soluble egg antigen. SmCTF=*S mansoni* cercarial transformation fluid. SWAP=soluble adult worm antigen preparation.

∗ Sensitivity and specificity were assumed constant across studies when the number of comparisons was less than four.

A total of seven comparisons in six studies (two including Kato-Katz thick smears,^49^^,^^52^ four including urine filtration)^25^^,^^60^^,^^64^^,^^65^ evaluated the diagnostic test circulating anodic antigen serum and urine assay (CAA; prevalence range 18·1–91·0%; table 2; appendix 2 pp 10–15). Forest and HSROC plots reveal a high degree of heterogeneity for the estimates (appendix 1 pp 22–23). When compared with duplicate Kato-Katz thick smears, individual estimates ranged from 10% to 90% for sensitivity and from 37% to 99% for specificity. Sensitivity ranged from 16% to 97% and specificity ranged from 24% to 100% for comparisons of CAA to urine filtration. Assuming imperfect standard tests, CAA was estimated to have a sensitivity of 90% (95% CrI 86–93) and a specificity of 95% (91–98) against Kato-Katz thick smears, and a sensitivity of 81% (24–99) and a specificity of 83% (25–100) against urine filtration (table 2).

**Figure 3:**
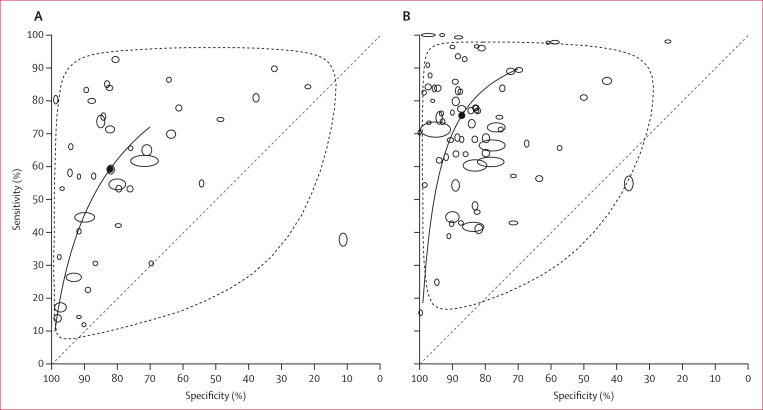
HSROC plot with summary points (A) Proteinuria reagent strips versus urine filtration. B) Haematuria reagent strips versus urine filtration. Each unfilled circle represents the individual study estimates pair (sensitivity, specificity), and larger circles reflect a larger sample size. The filled circle is the meta-analytic estimates across the studies included in the meta-analysis, the solid line is the summary HSROC curve, and the dashed curve is the 95% prediction region for a future study. HSROC=hierarchical summary receiver operating characteristic.

The accuracy of proteinuria reagent strips was assessed against urine filtration in 41 studies published in 37 articles (with 42 test comparisons)^27^^,^^29^^,^^30^^,^^35^^,^^37^^,^^66^, ^67^, ^68^, ^69^, ^70^, ^71^, ^72^, ^73^, ^74^, ^75^, ^76^, ^77^, ^78^, ^79^, ^80^, ^81^, ^82^, ^83^, ^84^, ^85^, ^86^, ^87^, ^88^, ^89^, ^90^, ^91^, ^92^, ^93^, ^94^, ^95^, ^96^, ^97^ and the accuracy of haematuria reagent strips was assessed in 74 studies published in 67 articles (with 75 test comparisons; prevalence 0·1–88·6%; appendix 2 p 3).^24^^,^^26^, ^27^, ^28^, ^29^, ^30^, ^31^^,^^34^, ^35^, ^36^, ^37^, ^38^^,^^66^, ^67^, ^68^, ^69^, ^70^, ^71^, ^72^, ^73^, ^74^, ^75^, ^76^, ^77^, ^78^, ^79^, ^80^, ^81^, ^82^, ^83^, ^84^^,^^86^, ^87^, ^88^, ^89^, ^90^, ^91^, ^92^, ^93^, ^94^, ^95^, ^96^, ^97^, ^98^, ^99^, ^100^, ^101^, ^102^, ^103^, ^104^, ^105^, ^106^, ^107^, ^108^, ^109^, ^110^, ^111^, ^112^, ^113^, ^114^, ^115^, ^116^, ^117^, ^118^, ^119^, ^120^, ^121^ For proteinuria reagent strips, the individual study estimates for sensitivity ranged from 12% to 93% and ranged from 11% to 99% for specificity. For haematuria reagent plural, sensitivity varied between 16% and 100% and specificity between 24% and 100% (appendix 1 pp 24–25; appendix 2 pp 10–15). All analyses showed high heterogeneity (*I*^2^>95%; appendix 1 pp 35–36), also observed in the HSROC plots (figure 3), which show high sensitivity at low specificity values and high specificity at low sensitivity values but with the majority of points between 60% and 90% for both specificity and sensitivity, leading to large CIs. Compared with urine filtration, the meta-analytic estimates for proteinuria reagent strips were 73% (95% CrI 62–82) for sensitivity and 94% (89–98) for specificity and, for haematuria reagent strips, were 85% (80–90) for sensitivity and 96% (92–99) for specificity (table 2). When allowing for moderate conditional dependency, the pooled sensitivity decreased slightly (61% [95% CrI 48–73] for proteinuria and 77% [70–83] for haematuria reagent strips) and specificity results were similar to the main model (appendix 1 p 21).

The leukocyturia reagent strip was evaluated in five comparisons against urine filtration^35^^,^^73^^,^^86^^,^^88^ and estimated to have a sensitivity of 60% (95% CrI 38–81) and specificity of 69% (27–96; table 2). The forest plot and HSROC showed variable specificity estimates across studies (appendix 1 p 26).

Using quadruple Kato-Katz thick smears as a reference test, *S mansoni* cercarial transformation fluid rapid diagnostic test (SmCTF-RDT) was investigated in four comparisons^42^^,^^54^^,^^122^ and the HSROC revealed poor accuracy of the test (appendix 1 p 27), as well as substantial heterogeneity of sensitivity (*I*^2^=87%; appendix 1 pp 35–36). The meta-analytic sensitivity was 83% (95% CrI 33–99) and specificity was 39% (11–70; table 2).

Forest plots and HSROC for the remainder diagnostics, including molecular-based assays, are presented in appendix 1 (pp 28–33). The IgG ELISA test was studied in three studies against triplicate Kato-Katz thick smears,^123^, ^124^, ^125^ with high individual sensitivity estimates above 90% but variable specificity estimates ranging from 30% to 99% across studies. Two studies^33^^,^^126^ assessed the accuracy of *S mansoni* adult worm extract (AWE) and *S mansoni* soluble egg antigen (SEA) ELISA in reference to Kato-Katz thick smears and the forest plot shows consistent moderate specificity estimates (62% and 80%) and high sensitivity estimates (96% and 85%) across both studies. Soluble adult worm antigen preparation (SWAP) ELISA was investigated in two studies against Kato-Katz thick smears^56^^,^^126^ with high sensitivity (90% and 92%) but varying specificity (90% and 57%) estimates. IgG SEA ELISA was investigated against urine filtration in five studies^32^^,^^63^^,^^127^, ^128^, ^129^ and estimated to have a pooled sensitivity of 94% (95% CrI 72–100) and a pooled specificity of 88% (47–100). PCR was assessed against Kato-Katz thick smears in five comparisons published in three articles,^130^, ^131^, ^132^ with a pooled specificity of 87% (95% CrI 62–99) and a pooled sensitivity of 97% (84–100). Three studies^46^^,^^133^^,^^134^ totalling five comparisons, investigated real-time PCR (rtPCR) against Kato-Katz thick smears and yielded slightly lower meta-analytic estimates than PCR, with a pooled sensitivity of 92% (CrI 17–100) and specificity of 79% (20–100). Finally, five studies (two *vs* Kato-Katz thick smears^135^^,^^136^ and three *vs* urine filtration^34^^,^^137^^,^^138^) evaluated a loop-mediated isothermal amplification (LAMP) assay. Against Kato-Katz thick smears, LAMP had sensitivity above 90% and specificity ranging from 75% to 100%, whereas, against urine filtration, estimates varied between studies, with specificity ranging between 39% and 88% and sensitivity ranging between 72% and 100%.

Deeks funnel plots used to assess potential publication bias show a symmetrical funnel shape and suggest no publication bias (appendix 1 p 34). We rated the certainty of evidence for all test comparisons as moderate. We downgraded one point for imprecision, as the reference standards, Kato-Katz thick smears and urine filtration, were imperfect tests with low sensitivity at low infection intensities.

## Discussion

In this meta-analysis, we summarise the current body of published information on available diagnostic assays against the Kato-Katz test for *S mansoni* and urine filtration test for *S haematobium* as reference standards. We identified 121 studies meeting our inclusion criteria, assessing a suite of 28 diagnostic techniques. Among the index tests used to detect *S mansoni*, CCA1 tests were by far the most frequently reported (36 comparisons), followed by PCR and rtPCR (each investigated in five comparisons). Haematuria (75 comparisons), proteinuria (42 comparisons), IgG ELISA (five comparisons), and leukocyturia (five comparisons) reagent strips were the index tests most reported for the detection of *S haematobium*. Our analyses suggest that CCA1 for *S mansoni* detects a large proportion of infections identified by the Kato-Katz test (sensitivity 95%) and has a reasonable specificity (74%). A Cochrane meta-analysis including 90 studies published before Feb 12, 2014^139^ produced a similar sensitivity estimate (89%), but a considerably lower specificity estimate (55%). The main reason for this difference lies in the statistical modelling. The Cochrane review used a Bayesian bivariate model assuming perfect reference standards, whereas we modelled the diagnostic accuracy through a hierarchical Bayesian latent-class model acknowledging the imperfections of the reference test.^140^^,^^141^ Our findings for CCA1 tests are in line with those of Colley and colleagues,^53^ who assessed CCA1 against Kato-Katz tests in a multicentre study and adjusted the analyses for imperfect standard tests (sensitivity 89%; specificity 72%). A 2018 study that translated the Kato-Katz thick smear prevalence to a range of CCA prevalence as a result of statistical modelling recommended replacing Kato-Katz thick smears with CCA in low-endemic settings based on the good performance of CCA.^142^ However, data from a 2022 study highlighted the need for standardisation of the point-of-care CCA.^143^ Straily and colleagues^144^ observed a non-linear relationship between both tests and issues with CCA specificity and sensitivity in low-prevalence settings, thereby making CCA not optimally suited for control programmes. Clark and colleagues^145^ also used Bayesian latent-class analysis to help guide policy for control and found that CCA could be used at the population level, but not for low-intensity infections at the individual level.

For the detection of *S haematobium*, our results suggest that haematuria reagent strips detect the largest proportion of infections (sensitivity 85%) and non-infections (specificity 96%) identified by microscopy. Proteinuria reagent strips had similar specificity to haematuria reagent strips (94%) but lower sensitivity (73%), whereas leukocyturia reagent strips had lower sensitivity (60%) and specificity (69%) summary estimates. The superior performance of haematuria over proteinuria reagent strips has also been observed in the Cochrane meta-analysis^139^ but was not statistically significant. Our estimates are similar to those of another 2013 meta-analysis^146^ that assessed only haematuria reagent strips against urine filtration (85% *vs* 81% for sensitivity; 96% *vs* 89% for specificity).

Despite little data, CAA compared with the reference tests provided preliminary promising sensitivity and specificity results (sensitivity and specificity estimates around 80% for *S haematobium* and 90% for *S mansoni*). In addition to sparse data, this test is not very applicable in field settings^147^ given that it is still under development. Efforts to provide a commercially available test are currently ongoing^148^ and studies (data not shown) carried out within the Schistosomiasis Consortium for Operational Research and Evaluation (SCORE) with dry reagents showed promising improvements in sensitivity for both species for CAA. On the contrary, SmCTF-RDT has very low specificity (39%), limiting its usefulness in practice. Compared with Kato-Katz thick smears, the IgG and AWE-SEA ELISA tests have a performance similar to CCA1 tests, but the low number of comparisons and the wide variation of specificity reduce the reliability of conclusions, which is a problem also highlighted by Graeff-Teixeira and colleagues.^149^

Finally, qualitative PCR and LAMP showed promising performance (sensitivity ≥90%); however, these tests need further validation and investigation as the sample sizes were small and the number of studies was limited.^144^ Our results align with the conclusions of two reviews by Diego and colleagues^150^ and Li and colleagues.^151^ Moreover, nucleic acid amplification tests (NAATs) are currently only applied in research settings, but not in routine health care. Qualitative PCR techniques require complex extraction methods and are time consuming, but LAMP is a more user-friendly technique that warrants further research in view of large-scale diagnostic surveys.

Our meta-analysis has several strengths, including a comprehensive search and a standardised data extraction and analyses accounting for imperfect reference tests and allowing the diagnostic accuracy to vary across studies. Informative priors in the Bayesian model were only used for the diagnostic accuracy of the reference tests. For the index tests, we chose non-informative priors to allow posterior distributions to be informed by data rather than by priors. The modelling accounted for heterogeneity across countries (ie*,* diagnostic accuracy or prevalence). Except for the reference tests Kato-Katz thick smears and urine filtration, we chose not to lump together similar index tests and we chose to analyse them as they were presented in the individual studies. This decision included, for instance, how authors managed trace positive results for antibody-based assays.

The main limitation of our study is the small number of studies and the small sample size for some test comparisons. When data were sparse, the accuracy of the tests was assumed to be constant across studies, which might lead to biased estimates,^152^ although parsimonious models are recommended in empirical studies or meta-analyses including small numbers of studies.^153^ Since we finalised our analyses, we checked for new studies and found that nine that met our inclusion criteria were published between Jan 1 to April 23, 2023, of which two were reviews^154^^,^^155^ and seven original research articles.^156^, ^157^, ^158^, ^159^, ^160^, ^161^, ^162^, ^163^ The two reviews were a meta-analysis evaluating the accuracy of antigen, antibody, and molecular tests for *S mansoni* and *S haematobium* only that did not use Bayesian latent class models, and a scoping review on proteins for antibody-based tests. The original research articles were on point-of-care CCA (two articles), CRISPR (one article), multidiagnostic microscope (one article), and Helminthex (one article).^156^, ^157^, ^158^, ^159^, ^160^, ^161^, ^162^, ^163^ These articles might have added some value to our comparisons based on CCA, CAA, and Helminthex. One research study was done in Ontario, Canada. The last article assessed diagnostic tests of *S japonicum*.

We also found issues related to substantial heterogeneity in the sensitivity and specificity estimates across studies, which included the use as reference test for *S mansoni* of multiple repeats (up to 27) of the Kato-Katz test (beyond the WHO-recommended standard) and the absence of information on infection intensity, treatment history, and age of participants. The accuracy of diagnostics probably varies by setting and is affected by disease prevalence and infection intensity.^16^^,^^164^ Previous meta-analyses have found that, independent of prevalence, haematuria reagent strips performed better in school-aged children than in adults.^139^^,^^146^

There should be some caution when interpreting our findings. First, as sample sizes were relatively small and reporting of covariates was insufficient, we were unable to explore potential sources of heterogeneity using a meta-regression approach. Second, Deeks funnel plots have low power to detect asymmetry in the presence of high heterogeneity.^165^ Third, nearly 70% of the studies had an unclear risk of bias for the reference standard or the index tests, which is similar to the results observed by Ochodo and colleagues.^139^ Knowing the risk of bias of these studies would help to assess the strength of evidence more objectively. We encourage all study authors to follow the Standards for Reporting of Diagnostic Accuracy Studies (STARD) guidelines for reporting diagnostic studies.^166^

Based on the information provided in the articles, we grouped all circulating antigen-based assays (CCA1, circulating cathodic antigen urine cassette assay version 2 [CCA2], or CAA tests) together for the pooled analyses. However, the format of these assays might vary and can be performed using ELISA or using upconverting phosphor and lateral flow (UCP-LF) technology. Sensitivity might vary depending on the test format, especially for low antigen concentrations. This variation makes it challenging to recommend one specific format and, despite the good results for CCA and promising results for CAA, advice about the use of any alternative test would be guided by the use-case. Another shortcoming of CCA is the variable quality of different batches of the test, subjective reading, and non-specificity in non-endemic areas. Information on these aspects was not available from the articles used for the review.

In conclusion, the availability of sensitive and specific diagnostic tools is crucial for accurate surveillance of schistosomiasis and the move towards the elimination of schistosomiasis. Our meta-analysis and systematic review cannot identify a suitable practicable alternative to conventional diagnostic assays (ie, Kato-Katz tests for *S mansoni* and urine filtration for *S haematobium*), even though microscopy has low sensitivity for light infections. New alternative diagnostic tools, including NAATs and immunological diagnostics, show promise but there is little sufficient data on their diagnostic accuracy. Several criteria should be taken into consideration when choosing a diagnostic test, including use-case (eg, for surveillance in the control or the elimination phase, or case management), accuracy, deployability, cost, and acceptability. Although conventional methods are well accepted, relatively cheap, and easily deployed, new tools often lack easy-to-use, commercially ready forms and might require specific laboratory facilities and resources. Additional research, including into the diagnostic accuracy in low-prevalence areas and the cost-effectiveness of new assays, is needed if a suitable replacement for the standard references is to be reliably recommended.

## Data sharing

The data supporting this systematic review and meta-analysis are from previously reported studies, which have all been cited. The processed data are available from the corresponding author on reasonable request. Aggregated data from the articles used for the analyses are provided in appendix 2 (pp 10–15).

## Declaration of interests

We declare no competing interests.
