# Supplementary material for: Diagnostic tests for human Schistosoma mansoni and Schistosoma haematobium infection: a systematic review and meta-analysis
Source: Lancet Microbe. 2024 Apr;5(4):e366–78. doi: 10.1016/S2666-5247(23)00377-4 (PMC10990967; doi:10.1016/S2666-5247(23)00377-4)
Supplement: Supplementary appendix 1 [file mmc1.pdf]

THE LANCET
Microbe

Supplementary appendix 1

This appendix formed part of the original submission and has been peer reviewed. We post it as supplied by the authors.

Supplement to: Vaillant MT, Philippy F, Neven A, et al. Diagnostic tests for human *Schistosoma mansoni* and *Schistosoma haematobium* infection: a systematic review and meta-analysis. *Lancet Microbe* 2024. https://doi.org/10.1016/S2666-5247(23)00377-4

Table of Contents

1 : supplement to the manuscript 2

1.1: Full Background 2

1.2 : Search strategy 3

1.3: Statistical methodology 4

2: References of all included studies (1-121) 7

3: Quality assessment of included studies 14

3.1: Risk of bias and applicability concerns graph: review authors' judgements about each domain presented as percentages across the included studies 14

3.2: Risk of bias and applicability concerns summary: review author's judgements about each domain for each included study 15

4: Additional figures and tables 18

4.1: Prevalence, sensitivity (Se) and specificity (Sp) for diagnostic tests investigated in one single study 18

4.2: Forest plot - Sensitivity and specificity of CCA1 versus 19

4.3: Pooled Se and Sp in models incorporating conditional dependence 21

4.4: Forest plot – Sensitivity and specificity of CAA versus 22

4.5: Hierarchical summary Receiver Operating Characteristic Plot with summary points 23

4.6: Forest plot – Sensitivity and specificity 24

4.7: Leukocyturia vs urine microscopy 26

4.8: SmCTF-RDT vs quadruple Kato-Katz smears 27

4.9: Forest plots - Sensitivity and specificity 28

4.10: Hierarchical summary Receiver Operating Characteristic Plots with summary point 30

4.11: Deek's funnel plots whenever data from more than four studies were available 34

4.12: Assessment of heterogeneity for Se, Sp and DOR whenever data from more than four studies were available 35

1 : supplement to the manuscript

1.1: Full Background

Schistosomiasis is a parasitic disease caused by blood flukes of the genus Schistosoma with an acute phase followed by a chronic phase due to repeated exposure to the parasite if left untreated. Transmission occurs when people performing their agricultural, housing, professional or recreational usual activities are exposed to freshwater sources contaminated with excreta containing parasite eggs1-3. The roadmap for neglected tropical diseases (NTDs), first launched in 20124, followed by the London Declaration of NTDs5 created a new emphasis for the control of schistosomiasis with a new roadmap, which targets elimination of schistosomiasis by 20306.

Updated WHO guidelines on control and elimination of schistosomiasis were published in February 20227 to revise according to the latest scientific evidence using appropriate methodologies8, 9. The goal of the WHO guidelines is to provide evidence-based recommendations to countries in their effort to move from morbidity control to interruption of transmission10, 11.

Accurate diagnostic tools play a crucial role in implementing strategies for the surveillance, control and elimination of schistosomiasis12. Six Schistosoma species cause the human chronic disease, but most infections and the bulk of the global burden are due to Schistosoma mansoni and S. haematobium. For S. mansoni, the conventional reference standard diagnostic test is duplicate Kato-Katz thick smears (KK) 13, 14. However, multiple thick smears from a single stool sample or multiple stool sample examinations with single or duplicate KK are also found in the literature. The main parasitological method for detecting infection with S. haematobium is urine filtration and microscopy and multiple filtrations over consecutive days were tested to detect very light infections12, 15. Both reference diagnostic tests rely on the identification of Schistosoma eggs.

To inform policy recommendations by a WHO Expert Group on diagnostic tools for human Schistosoma infections in the context of verification of transmission interruption, we performed a systematic review and meta-analysis. The present work was initiated as part of nine systematic reviews and meta-analyses performed for the purpose of the new WHO guidelines. Originally posted on MedRxiv on 9 May 2021 the work is updated until December 31, 2022. The objectives of the current work were to assess and compare the sensitivity (Se) and specificity (Sp) of a wide range of diagnostic tools, using KK for S. mansoni and urine filtration for S. haematobium as the reference standards. A recent systematic review and meta-analysis on S. japonicum and S. mekongii by Rahman et al was published in 202116. The current work complete the landscape of diagnostic tools accuracy for Schistosoma species.

1. Colley DG, Bustinduy AL, Secor WE, King CH. Human schistosomiasis. *Lancet* 2014; **383**(9936): 2253-64.
2. McManus DP, Dunne DW, Sacko M, Utzinger J, Vennervald BJ, Zhou XN. Schistosomiasis. *Nat Rev Dis Primers* 2018; **4**(1): 13.
3. Schistosomiasis and soil-transmitted helminthiases: progress report, 2020. *Wkly Epidemiol Rec* 2021; 96 (48): 585 - 595.
4. World Health Organization. Accelerating work to overcome the global impact of neglected tropical disease: a road map for implementation. Geneva: World Health Organization. ; 2012.

5. London Declaration on neglected tropical diseases. Uniting to Combat NeglectedTropical Diseases; . 2012. https://unitingtocombatntds.org/resource-hub/who-resources/london-declaration-neglected-tropical-diseases/ (accessed July 18 2022).
6. World Health Organization. Ending the neglect to attain the Sustainable Development Goals: a road map for neglected tropical diseases 2021–2030. Geneva: World Health Organization. ; 2020.
7. World Health Organization. WHO guideline on control and elimination of human schistosomiasis. Geneva: World Health Organization; 2022.
8. Lo NC, Bezerra FSM, Colley DG, et al. Review of 2022 WHO guidelines on the control and elimination of schistosomiasis. *Lancet Infect Dis* 2022; **22**(11): e327-e35.
9. Bärenbold O, Garba A, Colley DG, et al. Translating preventive chemotherapy prevalence thresholds for *Schistosoma mansoni* from the Kato-Katz technique into the point-of-care circulating cathodic antigen diagnostic test. *PLoS Negl Trop Dis* 2018; **12**(12): e0006941.
10. Kajihara N, Hirayama K. The War against a Regional Disease in Japan A History of the Eradication of Schistosomiasis japonica. *Trop Med Health* 2011; **39**(1 Suppl 1): 3-44.
11. Wang LD, Chen HG, Guo JG, et al. A strategy to control transmission of *Schistosoma japonicum* in China. *N Engl J Med* 2009; **360**(2): 121-8.
12. Utzinger J, Becker SL, van Lieshout L, van Dam GJ, Knopp S. New diagnostic tools in schistosomiasis. *Clin Microbiol Infect* 2015; **21**(6): 529-42.
13. Katz N, Chaves A, Pellegrino J. A simple device for quantitative stool thick-smear technique in Schistosomiasis mansoni. *Rev Inst Med Trop Sao Paulo* 1972; **14**(6): 397-400.
14. World Health Organization. Assessing the efficacy of anthelminthic drugs against schistosomiasis and soil-transmitted helminthiases.; 2013.
15. Weerakoon KG, Gobert GN, Cai P, McManus DP. Advances in the diagnosis of human schistosomiasis. *Clin Microbiol Rev* 2015; **28**(4): 939-67.

1.2 : Search strategy

In this systematic review and meta-analysis, we searched PubMed, EMBASE, the Cochrane Library and LILACS up to December 31, 2022 using a broad search strategy. Key words included ("schistosomiasis" OR "Schistosoma") AND ("diagnostic" OR "sensitivity" OR "specificity"). Details of the search strategy are presented in appendix S1. We initially searched the databases up to February 2021 to reflect the preparatory work on the new WHO guidelines for human schistosomiasis that started in October 20187. Subsequently, our search was updated to include all studies published until December 31, 2022. Citations from relevant articles were hand-searched to identify potential additional studies.

- Search in EMBASE
  - MeSH: (('schistosomiasis':ti OR 'schistosoma':ti OR 'schistosomiasis':kw OR 'schistosoma':kw) AND ('diagnostic*':ti OR 'diagnostic*':kw)) OR (('schistosomiasis':ti OR 'schistosoma':ti OR 'schistosomiasis':kw OR 'schistosoma':kw) AND ('specificity':ti OR 'specificity':kw OR 'sensitivity':ti OR 'sensitivity':kw))
- Search in PubMed
  - MeSH: ((("schistosomiasis"[Title] OR "schistosoma"[Title]) OR ("schistosomiasis"[Other Term] OR "schistosoma"[Other Term])) AND ("diagnostic*"[Title] OR "diagnostic*"[Other Term])) OR (("schistosomiasis"[Title] OR "schistosoma"[Title] OR ("schistosomiasis"[Other Term] OR "schistosoma"[Other

Term])) AND ("specificity"[Title] OR "sensitivity"[Title] OR ("specificity"[Other Term] OR "sensitivity"[Other Term]))))

    - 416 results

- Search in LILACS
    - MeSH: (schistosomiasis OR schistomosa) AND (diagnostic* OR sensitivity OR specificity) AND (db:("LILACS") AND la:("en"))
- Search in Cochrane
    - MeSH: (schistosomiasis OR schistosoma) AND (diagnostic* OR specificity OR sensitivity)

1.3: Statistical methodology

We derived estimates of the pooled Se and Sp with their 95% credible intervals (CrIs) for the two diagnostic tests under comparison using a Bayesian bivariate random effect model and non-informative priors22. We also plotted the hierarchical summary receiver operating characteristic curves (HSROC) 23. These meta-analytic approaches jointly model Se and Sp and account for both within- and between-study variability in Se and Sp as well as for the correlation between Se and Sp across studies.

For every test comparison included in more than four studies, we investigated publication bias using Deeks' funnel plots and asymmetry tests, as well as heterogeneity across studies with forest plots and the I^2 statistic[32]. However as the I2 statistic do not account for positivity threshold effect, heterogeneity was further investigated by using the HsROC curve showing the joint estimate of sensitivity and 1-specificity together with a 95% confidence and prediction region. The larger the prediction region is, the bigger the extent of statistical heterogeneity, assuming the model is correct. It gives 95% confidence that the true sensitivity and specificity of a future study will take place in this prediction region.

However, the underlying models require that the reference tests have perfect accuracy.

To acknowledge that the reference standards for schistosomiasis are imperfect tests with low Se, especially when applied in regions of low endemicity24, 25, we conducted in a second step a hierarchical Bayesian latent-class meta-analysis under conditional independence. The latent class model was parameterized using a random-effects approach, allowing for correction of the heterogeneity in Se and Sp across the included studies. When data from at least four studies were available, we applied the model developed by Chu et al. 26 allowing the accuracy of the tests to vary across studies. When fewer than four studies were available, the Se and Sp of the test were assumed to be constant across studies27. A more parsimonious model had to be used as convergence problems were observed with between-studies variations of Se and Sp. Convergence of models was assessed visually and with the Gelman-Rubin diagnostic28.

For the Bayesian latent class estimation, prior distributions for most parameters were non-informative. For the "true" schistosomiasis prevalence in each study and the hyper prior distributions of the index test Se and Sp (on the logit scale), we used a Beta(1,1) distribution. A Wishart

distribution W(2, I2) was used as multivariate prior for the random-effects covariance matrix. Finally, for the reference test, we used a Beta(80,20) prior distribution for Se (95% CI - 71.7% to 87.2%) and a Beta(90,10) prior for Sp (95% CI - 83.4% to 95.0%).

When data from different sampling efforts, thereby resulting in multiple KK thick smears for a single test result (e.g. duplicate or quadruple KK), were available, we also modelled diagnostic accuracy lumping together the different KK-based diagnostic types: for the same index test compared to several types of the reference test, duplicate, triplicate or more were pooled together across all concerned studies.

For diagnostic assay comparisons available in at least 10 studies, we performed a sensitivity analysis incorporating conditional dependence29. The relative conditional dependence for Se and Sp was specified at proportions ranging from 0 to 0.3 of the maximum possible conditional dependence, which corresponds to moderate conditional dependence. When data were sparse, statistical models with conditional dependencies resulted in convergence problems and overfitting, leading to unstable estimates. The reporting of infection intensity was not reported or reported either by arithmetic or geometric means, range of infection or proportions i.e. light moderate or heavy infections. It did not allow to account homogeneously for intensity of infection in a sensitivity analysis.

Posterior distributions were obtained by using the Markov chain Monte Carlo (MCMC) algorithm implemented with JAGS software accessed through R for the latent class model30 and with PROC MCMC in SAS for the bivariate model31. Forest plots and HSROC curves were generated using Review Manager 5.4.

Statistical analyses were carried out by using the Review Manager V5 (Cochrane Collaboration, UK), SAS System V9.4 (SAS Institute, Cary, NC, US), R Studio V2022.07.1 and R V4.2.3 (The Foundation for Statistical Computing, US).

22. Reitsma JB, Glas AS, Rutjes AW, Scholten RJ, Bossuyt PM, Zwinderman AH. Bivariate analysis of sensitivity and specificity produces informative summary measures in diagnostic reviews. *J Clin Epidemiol* 2005; **58**(10): 982-90.
23. Rutter CM, Gatsonis CA. A hierarchical regression approach to meta-analysis of diagnostic test accuracy evaluations. *Stat Med* 2001; **20**(19): 2865-84.
24. Bärenbold O, Raso G, Coulibaly JT, N'Goran EK, Utzinger J, Vounatsou P. Estimating sensitivity of the Kato-Katz technique for the diagnosis of *Schistosoma mansoni* and hookworm in relation to infection intensity. *PLoS Negl Trop Dis* 2017; **11**(10): e0005953.
25. Stete K, Krauth SJ, Coulibaly JT, et al. Dynamics of *Schistosoma haematobium* egg output and associated infection parameters following treatment with praziquantel in school-aged children. *Parasit Vectors* 2012; **5**: 298.
26. Chu H, Chen S, Louis TA. Random effects models in a meta-analysis of the accuracy of two diagnostic tests without a gold standard. *J Am Stat Assoc* 2009; **104**(486): 512-23.
27. Hui SL, Walter SD. Estimating the error rates of diagnostic tests. *Biometrics* 1980; **36**(1): 167-71.

28. Gelman A, Carlin JB, Stern HS, Rubin DB. Bayesian Data Analysis. 2nd ed. London: Chapman & Hall; 2004.
29. Vacek PM. The effect of conditional dependence on the evaluation of diagnostic tests. *Biometrics* 1985; **41**(4): 959-68.
30. M. P. rJAGS: Bayesian graphical models using MCMC. https://cran.r-project.org/web/packages/rjags/rjags.pdf.
31. Menke J. Bivariate random-effects meta-analysis of sensitivity and specificity with the Bayesian SAS PROC MCMC: methodology and empirical evaluation in 50 meta-analyses. *Med Decis Making* 2013; **33**(5): 692-701.
32. Higgins JP, Thompson SG. Quantifying heterogeneity in a meta-analysis. *Stat Med* 2002; **21**(11): 1539-58.

2: References of all included studies (1-121)

1. Abdel-Wahab MF, Esmat G, Ramzy I, Fouad R, Abdel-Rahman M, Yosery A, et al. *Schistosoma haematobium* infection in Egyptian schoolchildren: demonstration of both hepatic and urinary tract morbidity by ultrasonography. Trans R Soc Trop Med Hyg. 1992;86(4):406-9.
2. Abdel-Wahab MF, Esmat G, Ramzy I, Narooz S, Medhat E, Ibrahim M, et al. The epidemiology of schistosomiasis in Egypt: Fayoum Governorate. Am J Trop Med Hyg. 2000;62(2 Suppl):55-64.
3. Adriko M, Standley CJ, Tinkitina B, Tukahebwa EM, Fenwick A, Fleming FM, et al. Evaluation of circulating cathodic antigen (CCA) urine-cassette assay as a survey tool for *Schistosoma mansoni* in different transmission settings within Bugiri district, Uganda. Acta Trop. 2014;136:50-7.
4. Al-Shehri H, Koukounari A, Stanton MC, Adriko M, Arinaitwe M, Atuhaire A, et al. Surveillance of intestinal schistosomiasis during control: a comparison of four diagnostic tests across five Ugandan primary schools in the Lake Albert region. Parasitology. 2018;145(13):1715-22.
5. Al-Sherbiny MM, Osman AM, Hancock K, Deelder AM, Tsang VC. Application of immunodiagnostic assays: detection of antibodies and circulating antigens in human schistosomiasis and correlation with clinical findings. Am J Trop Med Hyg. 1999;60(6):960-6.
6. Anosike JC, Nwoke BE, Njoku AJ. The validity of haematuria in the community diagnosis of urinary schistosomiasis infections. J Helminthol. 2001;75(3):223-5.
7. Anyan WK, Pulkkila BR, Dyra CE, Price M, Naples JM, Quartey JK, et al. Assessment of dual schistosome infection prevalence from urine in an endemic community of Ghana by molecular diagnostic approach. Parasite Epidemiol Control. 2020;9:e00130.
8. Aryeetey ME, Wagatsuma Y, Yeboah G, Asante M, Mensah G, Nkrumah FK, et al. Urinary schistosomiasis in southern Ghana: 1. Prevalence and morbidity assessment in three (defined) rural areas drained by the Densu River. Parasitol Int. 2000;49(2):155-63.
9. Assaré RK, Tra MBI, Ouattara M, Hürlimann E, Coulibaly JT, N'Goran EK, et al. Sensitivity of the point-of-care circulating cathodic antigen urine cassette test for diagnosis of *Schistosoma mansoni* in low-endemicity settings in Côte d'Ivoire. Am J Trop Med Hyg. 2018;99(6):1567-72.
10. Ayele B, Erko B, Legesse M, Hailu A, Medhin G. Evaluation of circulating cathodic antigen (CCA) strip for diagnosis of urinary schistosomiasis in Hassoba school children, Afar, Ethiopia. Parasite. 2008;15(1):69-75.
11. Barakat RM, El-Gassim EE, Awadalla HN, El-Molla A, Omer EA. Evaluation of enzyme linked immunosorbent assay (ELISA) as a diagnostic tool for schistosomiasis. Trans R Soc Trop Med Hyg. 1983;77(1):109-11.
12. Bassiouny HK, Hasab AA, El-Nimr NA, Al-Shibani LA, Al-Waleedi AA. Rapid diagnosis of schistosomiasis in Yemen using a simple questionnaire and urine reagent strips. East Mediterr Health J. 2014;20(4):242-9.
13. Bayoumi A, Al-Refai SA, Badr MS, Abd El-Aal AA, El Akkad DMH, Saad N, et al. Loop-Mediated Isothermal Amplification (Lamp): Sensitive and Rapid Detection of Schistosoma Haematobium DNA in Urine Samples of Egyptian Suspected Cases. J Egypt Soc Parasitol. 2016;46(2):299-308.
14. Bezerra DF, Pinheiro MCC, Barbosa L, Viana AG, Fujiwara RT, Bezerra FSM. Diagnostic comparison of stool exam and point-of-care circulating cathodic antigen (POC-CCA) test for schistosomiasis mansoni diagnosis in a high endemicity area in northeastern Brazil. Parasitology. 2021;148(4):420-6.
15. Birrie H, Medhin G, Jemaneh L. Comparison of urine filtration and a chemical reagent strip in the diagnosis of urinary schistosomiasis in Ethiopia. East Afr Med J. 1995;72(3):180-5.
16. Bocanegra C, Gallego S, Mendioroz J, Moreno M, Sulleiro E, Salvador F, et al. Epidemiology of schistosomiasis and usefulness of indirect diagnostic tests in school-age children in Cubal, Central Angola. PLoS Negl Trop Dis. 2015;9(10):e0004055.
17. Bogoch, II, Andrews JR, Dadzie Ephraim RK, Utzinger J. Simple questionnaire and urine reagent strips compared to microscopy for the diagnosis of *Schistosoma haematobium* in a community in northern Ghana. Trop Med Int Health. 2012;17(10):1217-21.

18. Bosompem KM, Ayi I, Anyan WK, Nkrumah FK, Kojima S. Limited field evaluation of a rapid monoclonal antibody-based dipstick assay for urinary schistosomiasis. Hybridoma. 1996;15(6):443-7.
19. Bosompem KM, Owusu O, Okanla EO, Kojima S. Applicability of a monoclonal antibody-based dipstick in diagnosis of urinary schistosomiasis in the Central Region of Ghana. Trop Med Int Health. 2004;9(9):991-6.
20. Bouilhac M, Le Bras J, Payet M, Savel J, Coulaud JP. [Comparative sensitivity of I. F. A. T. using adult Schistosoma antigen and E. L. I. S. A. with ovular antigen in the immunodiagnosis of schistosomiasis (author's transl)]. Bull Soc Pathol Exot Filiales. 1981;74(6):668-75.
21. Chernet A, Kling K, Sydow V, Kuenzli E, Hatz C, Utzinger J, et al. Accuracy of diagnostic tests for *Schistosoma mansoni* infection in asymptomatic Eritrean refugees: serology and point-of-care circulating cathodic antigen against stool microscopy. Clin Infect Dis. 2017;65(4):568-74.
22. Colley DG, Binder S, Campbell C, King CH, Tchuem Tchuenté LA, N'Goran EK, et al. A five-country evaluation of a point-of-care circulating cathodic antigen urine assay for the prevalence of *Schistosoma mansoni*. Am J Trop Med Hyg. 2013;88(3):426-32.
23. Cooppan RM, Schutte CH, Dingle CE, van Deventer JM, Becker PJ. Urinalysis reagent strips in the screening of children for urinary schistosomiasis in the RSA. S Afr Med J. 1987;72(7):459-62.
24. Coulibaly JT, N'Goran EK, Utzinger J, Doenhoff MJ, Dawson EM. A new rapid diagnostic test for detection of anti-*Schistosoma mansoni* and anti-*Schistosoma haematobium* antibodies. Parasit Vectors. 2013;6:29.
25. Coulibaly JT, Silue KD, Armstrong M, Diaz de Leon Derby M, D'Ambrosio MV, Fletcher DA, et al. High Sensitivity of Mobile Phone Microscopy Screening for Schistosoma haematobium in Azaguie, Cote d'Ivoire. Am J Trop Med Hyg. 2023;108(1):41-3.
26. Dawson EM, Sousa-Figueiredo JC, Kabatereine NB, Doenhoff MJ, Stothard JR. Intestinal schistosomiasis in pre school-aged children of Lake Albert, Uganda: diagnostic accuracy of a rapid test for detection of anti-schistosome antibodies. Trans R Soc Trop Med Hyg. 2013;107(10):639-47.
27. De Clercq D, Sacko M, Vercruysse J, Diarra A, Landoure A, vanden Bussche V, et al. Comparison of the circulating anodic antigen detection assay and urine filtration to diagnose *Schistosoma haematobium* infections in Mali. Trans R Soc Trop Med Hyg. 1995;89(4):395-7.
28. Deribew K, Yewhalaw D, Erko B, Mekonnen Z. Urogenital schistosomiasis prevalence and diagnostic performance of urine filtration and urinalysis reagent strip in schoolchildren, Ethiopia. PLoS One. 2022;17(7):e0271569.
29. El-Morshedy H, Kinosien B, Barakat R, Omer E, Khamis N, Deelder AM, et al. Circulating anodic antigen for detection of *Schistosoma mansoni* infection in Egyptian patients. Am J Trop Med Hyg. 1996;54(2):149-53.
30. el-Sayed HF, Rizkalla NH, Mehanna S, Abaza SM, Winch PJ. Prevalence and epidemiology of *Schistosoma mansoni* and *S. haematobium* infection in two areas of Egypt recently reclaimed from the desert. Am J Trop Med Hyg. 1995;52(2):194-8.
31. Elbasheir MM, Karti IA, Elamin EM. Evaluation of a rapid diagnostic test for *Schistosoma mansoni* infection based on the detection of circulating cathodic antigen in urine in Central Sudan. PLoS Negl Trop Dis. 2020;14(6):e0008313.
32. Eltiro F, Ye-ebiyo Y, Taylor MG. Evaluation of an enzyme linked immunosorbent assay (ELISA) using *Schistosoma mansoni* soluble egg antigen as a diagnostic tool for *Schistosoma mansoni* infection in Ethiopian schoolchildren. J Trop Med Hyg. 1992;95(1):52-6.
33. Eltoum IA, Sulaiman S, Ismail BM, Ali MM, Elfatih M, Homeida MM. Evaluation of haematuria as an indirect screening test for schistosomiasis haematobium: a population-based study in the White Nile province, Sudan. Acta Trop. 1992;51(2):151-7.
34. Espirito-Santo MC, Alvarado-Mora MV, Pinto PL, Sanchez MC, Dias-Neto E, Castilho VL, et al. Comparative study of the accuracy of different techniques for the laboratory diagnosis of schistosomiasis mansoni in areas of low endemicity in Barra Mansa city, Rio de Janeiro State, Brazil. Biomed Res Int. 2015;2015:135689.

35. Fatiregun A, Osungbade K, Olumide E. Diagnostic performance of screening methods for urinary schistosomiasis in a school-based control programme, in Ibadan, Nigeria. Journal of Community Medicine and Primary Health Care. 2005;17(1):24-7.
36. Ferreira FT, Fidelis TA, Pereira TA, Otoni A, Queiroz LC, Amancio FF, et al. Sensitivity and specificity of the circulating cathodic antigen rapid urine test in the diagnosis of Schistosomiasis mansoni infection and evaluation of morbidity in a low- endemic area in Brazil. Rev Soc Bras Med Trop. 2017;50(3):358-64.
37. French MD, Rollinson D, Basanez MG, Mgeni AF, Khamis IS, Stothard JR. School-based control of urinary schistosomiasis on Zanzibar, Tanzania: monitoring micro-haematuria with reagent strips as a rapid urological assessment. J Pediatr Urol. 2007;3(5):364-8.
38. Fuss A, Mazigo HD, Tappe D, Kasang C, Mueller A. Comparison of sensitivity and specificity of three diagnostic tests to detect *Schistosoma mansoni* infections in school children in Mwanza region, Tanzania. PLoS One. 2018;13(8):e0202499.
39. Gabr NS, Hammad TA, Orieby A, Shawky E, Khattab MA, Strickland GT. The epidemiology of schistosomiasis in Egypt: Minya Governorate. Am J Trop Med Hyg. 2000;62(2 Suppl):65-72.
40. Gandasegui J, Fernandez-Soto P, Carranza-Rodriguez C, Perez-Arellano JL, Vicente B, Lopez-Aban J, et al. The rapid-heat LAMPellet method: a potential diagnostic method for human urogenital schistosomiasis. PLoS Negl Trop Dis. 2015;9(7):e0003963.
41. Gandasegui J, Fernandez-Soto P, Dacal E, Rodriguez E, Saugar JM, Yepes E, et al. Field and laboratory comparative evaluation of a LAMP assay for the diagnosis of urogenital schistosomiasis in Cubal, Central Angola. Trop Med Int Health. 2018;23(9):992-1001.
42. Gandasegui J, Fernandez-Soto P, Muro A, Simoes Barbosa C, Lopes de Melo F, Loyo R, et al. A field survey using LAMP assay for detection of Schistosoma mansoni in a low-transmission area of schistosomiasis in Umbuzeiro, Brazil: Assessment in human and snail samples. PLoS Negl Trop Dis. 2018;12(3):e0006314.
43. Glinz D, Silué KD, Knopp S, Lohourignon LK, Yao KP, Steinmann P, et al. Comparing diagnostic accuracy of Kato-Katz, Koga agar plate, ether-concentration, and FLOTAC for *Schistosoma mansoni* and soil-transmitted helminths. PLoS Negl Trop Dis. 2010;4(7):e754.
44. Grenfell RF, Martins W, Enk M, Almeida A, Siqueira L, Silva-Moraes V, et al. Schistosoma mansoni in a low-prevalence area in Brazil: the importance of additional methods for the diagnosis of hard-to-detect individual carriers by low-cost immunological assays. Mem Inst Oswaldo Cruz. 2013;108(3):328-34.
45. Gundersen SG, Kjetland EF, Poggensee G, Helling-Giese G, Richter J, Chitsulo L, et al. Urine reagent strips for diagnosis of schistosomiasis haematobium in women of fertile age. Acta Trop. 1996;62(4):281-7.
46. Hammad TA, Gabr NS, Talaat MM, Orieby A, Shawky E, Strickland GT. Hematuria and proteinuria as predictors of *Schistosoma haematobium* infection. Am J Trop Med Hyg. 1997;57(3):363-7.
47. Hammam HM, Allam FA, Moftah FM, Abdel-Aty MA, Hany AH, Abd-El-Motagaly KF, et al. The epidemiology of schistosomiasis in Egypt: Assiut Governorate. Am J Trop Med Hyg. 2000;62(2 Suppl):73-9.
48. Hammam HM, Zarzour AH, Moftah FM, Abdel-Aty MA, Hany AH, El-Kady AY, et al. The epidemiology of schistosomiasis in Egypt: Qena Governorate. Am J Trop Med Hyg. 2000;62(2 Suppl):80-7.
49. Kassim OO. Proteinuria and haematuria as predictors of schistosomiasis in children. Ann Trop Paediatr. 1989;9(3):156-60.
50. Kiliku FM, Kimura E, Muhoho N, Migwi DK, Katsumata T. The usefulness of urinalysis reagent strips in selecting *Schistosoma haematobium* egg positives before and after treatment with praziquantel. J Trop Med Hyg. 1991;94(6):401-6.
51. King CH, Keating CE, Muruka JF, Ouma JH, Houser H, Siongok TK, et al. Urinary tract morbidity in schistosomiasis haematobia: associations with age and intensity of infection in an endemic area of Coast Province, Kenya. Am J Trop Med Hyg. 1988;39(4):361-8.

52. King CH, Lombardi G, Lombardi C, Greenblatt R, Hodder S, Kinyanjui H, et al. Chemotherapy-based control of schistosomiasis haematobia. I. Metrifonate versus praziquantel in control of intensity and prevalence of infection. Am J Trop Med Hyg. 1988;39(3):295-305.
53. Kitange HM, Swai AB, McLarty DG, Alberti KG. Schistosomiasis prevalence after administration of praziquantel to school children in Melela village, Morogoro region, Tanzania. East Afr Med J. 1993;70(12):782-6.
54. Knopp S, Ame SM, Hattendorf J, Ali SM, Khamis IS, Bakar F, et al. Urogenital schistosomiasis elimination in Zanzibar: accuracy of urine filtration and haematuria reagent strips for diagnosing light intensity *Schistosoma haematobium* infections. Parasit Vectors. 2018;11(1):552.
55. Knopp S, Corstjens PL, Koukounari A, Cercamondi CI, Ame SM, Ali SM, et al. Sensitivity and specificity of a urine circulating anodic antigen test for the diagnosis of *Schistosoma haematobium* in low endemic settings. PLoS Negl Trop Dis. 2015;9(5):e0003752.
56. Kosinski KC, Bosompem KM, Stadecker MJ, Wagner AD, Plummer J, Durant JL, et al. Diagnostic accuracy of urine filtration and dipstick tests for *Schistosoma haematobium* infection in a lightly infected population of Ghanaian schoolchildren. Acta Trop. 2011;118(2):123-7.
57. Lamberton PH, Kabatereine NB, Oguttu DW, Fenwick A, Webster JP. Sensitivity and specificity of multiple Kato-Katz thick smears and a circulating cathodic antigen test for *Schistosoma mansoni* diagnosis pre- and post-repeated-praziquantel treatment. PLoS Negl Trop Dis. 2014;8(9):e3139.
58. Legesse M, Erko B. Field-based evaluation of a reagent strip test for diagnosis of schistosomiasis mansoni by detecting circulating cathodic antigen (CCA) in urine in low endemic area in Ethiopia. Parasite. 2008;15(2):151-5.
59. Lengeler C, Mshinda H, Morona D, deSavigny D. Urinary schistosomiasis: testing with urine filtration and reagent sticks for haematuria provides a comparable prevalence estimate. Acta Trop. 1993;53(1):39-50.
60. Lindholz CG, Favero V, Verissimo CM, Candido RRF, de Souza RP, Dos Santos RR, et al. Study of diagnostic accuracy of Helmintex, Kato-Katz, and POC-CCA methods for diagnosing intestinal schistosomiasis in Candeal, a low intensity transmission area in northeastern Brazil. PLoS Negl Trop Dis. 2018;12(3):e0006274.
61. Lodh N, Mwansa JC, Mutengo MM, Shiff CJ. Diagnosis of *Schistosoma mansoni* without the stool: comparison of three diagnostic tests to detect *Schistosoma* [corrected] *mansoni* infection from filtered urine in Zambia. Am J Trop Med Hyg. 2013;89(1):46-50.
62. Mafe MA. The diagnostic potential of three indirect tests for urinary schistosomiasis in Nigeria. Acta Trop. 1997;68(3):277-84.
63. Mafe MA, von Stamm T, Utzinger J, N'Goran EK. Control of urinary schistosomiasis: an investigation into the effective use of questionnaires to identify high-risk communities and individuals in Niger State, Nigeria. Trop Med Int Health. 2000;5(1):53-63.
64. Magalhaes FDC, Resende SD, Senra C, Graeff-Teixeira C, Enk MJ, Coelho PMZ, et al. Accuracy of real-time polymerase chain reaction to detect *Schistosoma mansoni* - infected individuals from an endemic area with low parasite loads. Parasitology. 2020;147(10):1140-8.
65. Magnussen P, Ndawi B, Sheshe AK, Byskov J, Mbwana K, Christensen NO. The impact of a school health programme on the prevalence and morbidity of urinary schistosomiasis in Mwera division, Pangani district, Tanzania. Trans R Soc Trop Med Hyg. 2001;95(1):58-64.
66. Mahmoud DM, Saad GA, Bayoumi IR, Abdel-Hady ZM, Aminou HA. Evaluation of a developed IMB based-ELISA in diagnosis of urinary schistosomiasis in areas at risk in Upper Egypt. Parasitologists United Journal. 2021;14(1):63-71.
67. Mazigo HD, Kepha S, Kinung'hi SM. Sensitivity and specificity of point-of-care circulating cathodic antigen test before and after praziquantel treatment in diagnosing *Schistosoma mansoni* infection in adult population co-infected with human immunodeficiency virus-1, North-Western Tanzania. Arch Public Health. 2018;76:29.
68. Midzi N, Butterworth AE, Mduluza T, Munyati S, Deelder AM, van Dam GJ. Use of circulating cathodic antigen strips for the diagnosis of urinary schistosomiasis. Trans R Soc Trop Med Hyg. 2009;103(1):45-51.

69. Mohammed H, Landeryou T, Chernet M, Liyew EF, Wulataw Y, Getachew B, et al. Comparing the accuracy of two diagnostic methods for detection of light Schistosoma haematobium infection in an elimination setting in Wolaita Zone, South Western Ethiopia. PLoS One. 2022;17(4):e0267378.
70. Morenikeji O, Quazim J, Omoregie C, Hassan A, Nwuba R, Anumudu C, et al. A cross-sectional study on urogenital schistosomiasis in children; haematuria and proteinuria as diagnostic indicators in an endemic rural area of Nigeria. Afr Health Sci. 2014;14(2):390-6.
71. Mott KE, Dixon H, Osei-Tutu E, England EC, Ekue K, Tekle A. Indirect screening for *Schistosoma haematobium* infection: a comparative study in Ghana and Zambia. Bull World Health Organ. 1985;63(1):135-42.
72. Mtasiwa D, Mayombana C, Kilima P, Tanner M. Validation of reagent sticks in diagnosing urinary schistosomiasis in an urban setting. East Afr Med J. 1996;73(3):198-200.
73. Murare HM, Taylor P. Haematuria and proteinuria during *Schistosoma haematobium* infection: relationship to intensity of infection and the value of chemical reagent strips for pre- and post-treatment diagnosis. Trans R Soc Trop Med Hyg. 1987;81(3):426-30.
74. Mwangi IN, Agola EL, Mugambi RM, Shiraho EA, Mkoji GM. Development and evaluation of a loop-mediated isothermal amplification assay for diagnosis of *Schistosoma mansoni* infection in faecal samples. J Parasitol Res. 2018;2018:1267826.
75. N'Goran KE, Yapi Yapi Y, Rey JL, Soro B, Coulibaly A, Bellec C. [Screening for urinary Schistosoma by strips reactive to hematuria. Evaluation in zones of intermediate and weak endemicity in the Ivory Coast]. Bull Soc Pathol Exot Filiales. 1989;82(2):236-42.
76. Nausch N, Dawson EM, Midzi N, Mduluza T, Mutapi F, Doenhoff MJ. Field evaluation of a new antibody-based diagnostic for *Schistosoma haematobium* and *S. mansoni* at the point-of-care in northeast Zimbabwe. BMC Infect Dis. 2014;14:165.
77. Navaratnam AM, Mutumba-Nakalembe MJ, Stothard JR, Kabatereine NB, Fenwick A, Sousa-Figueiredo JC. Notes on the use of urine-CCA dipsticks for detection of intestinal schistosomiasis in preschool children. Trans R Soc Trop Med Hyg. 2012;106(10):619-22.
78. Ndamukong KJ, Ayuk MA, Dinga JS, Akenji TN, Ndiforchu VA, Titanji VP. Prevalence and intensity of urinary schistosomiasis in primary school children of the Kotto Barombi Health Area, Cameroon. East Afr Med J. 2001;78(6):287-9.
79. Ndhlovu P, Cadman H, Gundersen S, Vennervald BJ, Friis H, Christensen NO, et al. Circulating anodic antigen (CAA) levels in different age groups in a Zimbabwean rural community endemic for *Schistosoma haematobium* determined using the magnetic beads antigen-capture enzyme-linked immunoassay. Am J Trop Med Hyg. 1996;54(5):537-42.
80. Nduka FO, Ajaero CM, Nwoke BE. Urinary schistosomiasis among school children in an endemic community in south-eastern Nigeria. Appl Parasitol. 1995;36(1):34-40.
81. Ndyomugyenyi R, Minjas JN. Urinary schistosomiasis in schoolchildren in Dar-es-Salaam, Tanzania, and the factors influencing its transmission. Ann Trop Med Parasitol. 2001;95(7):697-706.
82. Ng'andu NH. The use of Baye's theorem and other indices of agreement in evaluating the use of reagent strips in screening rural schoolchildren for *Schistosoma haematobium* in Zambia. Int J Epidemiol. 1988;17(1):202-8.
83. Ngasala B, Juma H, Mwaiswelo RO. The usefulness of indirect diagnostic tests for *Schistosoma haematobium* infection after repeated rounds of mass treatment with praziquantel in Mpwapwa and Chakechake districts in Tanzania. Int J Infect Dis. 2020;90:132-7.
84. Nwaorgu OC, Anigbo EU. The diagnostic value of haematuria and proteinuria in *Schistosoma haematobium* infection in southern Nigeria. J Helminthol. 1992;66(3):177-85.
85. Ofori-Adjei D, Adjepon-Yamoah KK, Ashitey GA, Osei-Tutu E. Screening methods for urinary schistosomiasis in an endemic area (the Kraboa/Coaltar district of Ghana). Ann Trop Med Parasitol. 1986;80(3):365-6.
86. Okeke OC, Ubachukwu PO. Performance of three rapid screening methods in the detection of *Schistosoma haematobium* infection in school-age children in Southeastern Nigeria. Pathog Glob Health. 2014;108(2):111-7.

87. Oliveira EJ, Kanamura HY, Lima DM. Efficacy of an enzyme-linked immunosorbent assay as a diagnostic tool for schistosomiasis mansoni in individuals with low worm burden. Mem Inst Oswaldo Cruz. 2005;100(4):421-5.
88. Oliveira LM, Santos HL, Goncalves MM, Barreto MG, Peralta JM. Evaluation of polymerase chain reaction as an additional tool for the diagnosis of low-intensity Schistosoma mansoni infection. Diagn Microbiol Infect Dis. 2010;68(4):416-21.
89. Onayade AA, Abayomi IO, Fabiyi AK. Urinary schistosomiasis: options for control within endemic rural communities: a case study in south-west Nigeria. Public Health. 1996;110(4):221-7.
90. Poggensee G, Krantz I, Kiwelu I, Feldmeier H. Screening of Tanzanian women of childbearing age for urinary schistosomiasis: validity of urine reagent strip readings and self-reported symptoms. Bull World Health Organ. 2000;78(4):542-8.
91. Polman K, Stelma FF, Gryseels B, Van Dam GJ, Talla I, Niang M, et al. Epidemiologic application of circulating antigen detection in a recent Schistosoma mansoni focus in northern Senegal. Am J Trop Med Hyg. 1995;53(2):152-7.
92. Pontes LA, Oliveira MC, Katz N, Dias-Neto E, Rabello A. Comparison of a polymerase chain reaction and the Kato-Katz technique for diagnosing infection with Schistosoma mansoni. Am J Trop Med Hyg. 2003;68(6):652-6.
93. Pugh RN, Bell DR, Gilles HM. Malumfashi Endemic Diseases Research Project, XV. The potential medical importance of bilharzia in northern Nigeria: a suggested rapid, cheap and effective solution for control of *Schistosoma haematobium* infection. Ann Trop Med Parasitol. 1980;74(6):597-613.
94. Rasendramino MH, Rajaona HR, Ramarokoto CE, Ravaoalimalala VE, Leutscher P, Cordonnier D, et al. [Prevalence of uro-nephrologic complications of urinary bilharziasis in hyperendemic focus in Madagascar]. Nephrologie. 1998;19(6):341-5.
95. Robinson E, Picon D, Sturrock HJ, Sabasio A, Lado M, Kolaczinski J, et al. The performance of haematuria reagent strips for the rapid mapping of urinary schistosomiasis: field experience from Southern Sudan. Trop Med Int Health. 2009;14(12):1484-7.
96. Rollinson D, Klinger EV, Mgeni AF, Khamis IS, Stothard JR. Urinary schistosomiasis on Zanzibar: application of two novel assays for the detection of excreted albumin and haemoglobin in urine. J Helminthol. 2005;79(3):199-206.
97. Sarda RK. Frequency of haematuria and proteinuria in relation to prevalence and intensity of *Schistosoma haematobium* infection in Dar es Salaam, Tanzania. East Afr Med J. 1986;63(2):105-8.
98. Savioli L, Hatz C, Dixon H, Kisumku UM, Mott KE. Control of morbidity due to *Schistosoma haematobium* on Pemba Island: egg excretion and hematuria as indicators of infection. Am J Trop Med Hyg. 1990;43(3):289-95.
99. Schunk M, Kebede Mekonnen S, Wondafrash B, Mengele C, Fleischmann E, Herbinger KH, et al. Use of Occult Blood Detection Cards for Real-Time PCR-Based Diagnosis of Schistosoma Mansoni Infection. PLoS One. 2015;10(9):e0137730.
100. Sellin B, Simonkovich E, Ovazza L, Sellin E, Desfontaine M, Rey JL. [Value of macroscopic urine examination and reagent strips for the detection of hematuria and proteinuria in the mass diagnosis of urinary schistosomiasis, before and after treatment]. Med Trop (Mars). 1982;42(5):521-6.
101. Senra C, Gomes LI, Siqueira LMV, Coelho PMZ, Rabello A, Oliveira E. Development of a laboratorial platform for diagnosis of schistosomiasis mansoni by PCR-ELISA. BMC Res Notes. 2018;11(1):455.
102. Shane HL, Verani JR, Abudho B, Montgomery SP, Blackstock AJ, Mwinzi PN, et al. Evaluation of urine CCA assays for detection of *Schistosoma mansoni* infection in Western Kenya. PLoS Negl Trop Dis. 2011;5(1):e951.
103. Shaw DJ, Picquet M, Ly A, Sambou B, Vercruysse J. Evaluation of dipsticks in *Schistosoma haematobium* infections in four villages in the middle valley of the Senegal River Basin, Senegal. Trans R Soc Trop Med Hyg. 1998;92(6):634-5.
104. Sheele JM, Kihara JH, Baddorf S, Byrne J, Ravi B. Evaluation of a novel rapid diagnostic test for *Schistosoma haematobium* based on the detection of human immunoglobulins bound to filtered *Schistosoma haematobium* eggs. Trop Med Int Health. 2013;18(4):477-84.

105. Song HB, Kim J, Jin Y, Lee JS, Jeoung HG, Lee YH, et al. Comparison of ELISA and urine microscopy for diagnosis of *Schistosoma haematobium* infection. J Korean Med Sci. 2018;33(33):e238.
106. Sousa SRM, Nogueira JFC, Dias IHL, Fonseca ALS, Favero V, Geiger SM, et al. The use of the circulating cathodic antigen (CCA) urine cassette assay for the diagnosis and assessment of cure of *Schistosoma mansoni* infections in an endemic area of the Amazon region. Rev Soc Bras Med Trop. 2020;53:e20190562.
107. Standley CJ, Lwambo NJ, Lange CN, Kariuki HC, Adriko M, Stothard JR. Performance of circulating cathodic antigen (CCA) urine-dipsticks for rapid detection of intestinal schistosomiasis in schoolchildren from shoreline communities of Lake Victoria. Parasit Vectors. 2010;3(1):7.
108. Stephenson LS, Latham MC, Kinoti SN, Oduori ML. Sensitivity and specificity of reagent strips in screening of Kenyan children for *Schistosoma haematobium* infection. Am J Trop Med Hyg. 1984;33(5):862-71.
109. Stothard JR, Sousa-Figueiredo JC, Standley C, Van Dam GJ, Knopp S, Utzinger J, et al. An evaluation of urine-CCA strip test and fingerprick blood SEA-ELISA for detection of urinary schistosomiasis in schoolchildren in Zanzibar. Acta Trop. 2009;111(1):64-70.
110. Russell Stothard J, Sousa-Figueiredo JC, Simba Khamis I, Garba A, Rollinson D. Urinary schistosomiasis-associated morbidity in schoolchildren detected with urine albumin-to-creatinine ratio (UACR) reagent strips. J Pediatr Urol. 2009;5(4):287-91.
111. Tanner M, Holzer B, Marti HP, Saladin B, Degremont AA. Frequency of haematuria and proteinuria among *Schistosoma haematobium* infected children of two communities from Liberia and Tanzania. Acta Trop. 1983;40(3):231-7.
112. Tchuem Tchuenté LA, Kuete Fouodo CJ, Kamwa Ngassam RI, Sumo L, Dongmo Noumedem C, Kenfack CM, et al. Evaluation of circulating cathodic antigen (CCA) urine-tests for diagnosis of *Schistosoma mansoni* infection in Cameroon. PLoS Negl Trop Dis. 2012;6(7):e1758.
113. Traore M, Traore HA, Kardorff R, Diarra A, Landoure A, Vester U, et al. The public health significance of urinary schistosomiasis as a cause of morbidity in two districts in Mali. Am J Trop Med Hyg. 1998;59(3):407-13.
114. Uga S, Gatika SM, Kimura E, Muhoho DN, Waiyaki PG. Enzyme-linked immunosorbent assay as a diagnostic method for schistosomiasis haematobium. Standardization and application in the field. J Trop Med Hyg. 1989;92(6):407-11.
115. Ugbomoiko US, Dalumo V, Ariza L, Bezerra FS, Heukelbach J. A simple approach improving the performance of urine reagent strips for rapid diagnosis of urinary schistosomiasis in Nigerian schoolchildren. Mem Inst Oswaldo Cruz. 2009;104(3):456-61.
116. Ugbomoiko US, Obiezue RN, Ogunniyi TA, Ofoezie IE. Diagnostic accuracy of different urine dipsticks to detect urinary schistosomiasis: a comparative study in five endemic communities in Osun and Ogun States, Nigeria. J Helminthol. 2009;83(3):203-9.
117. Van Lieshout L, Panday UG, De Jonge N, Krijger FW, Oostburg BF, Polderman AM, et al. Immunodiagnosis of schistosomiasis mansoni in a low endemic area in Surinam by determination of the circulating antigens CAA and CCA. Acta Trop. 1995;59(1):19-29.
118. Verle P, Stelma F, Desreumaux P, Dieng A, Diaw O, Kongs A, et al. Preliminary study of urinary schistosomiasis in a village in the delta of the Senegal river basin, Senegal. Trans R Soc Trop Med Hyg. 1994;88(4):401-5.
119. Wilkins HA, Goll P, Marshall TF, Moore P. The significance of proteinuria and haematuria in *Schistosoma haematobium* infection. Trans R Soc Trop Med Hyg. 1979;73(1):74-80.
120. Zhang LJ, Mwanakasale V, Xu J, Sun LP, Yin XM, Zhang JF, et al. Diagnostic performance of two specific *Schistosoma japonicum* immunological tests for screening *Schistosoma haematobium* in school children in Zambia. Acta Trop. 2020;202:105285.
121. Zumstein A. A study of some factors influencing the epidemiology of urinary schistosomiasis at Ifakara (Kilombero District, Morogoro Region, Tanzania). Acta Trop. 1983;40(3):187-204.

3: Quality assessment of included studies

3.1: Risk of bias and applicability concerns graph: review authors' judgements about each domain presented as percentages across the included studies

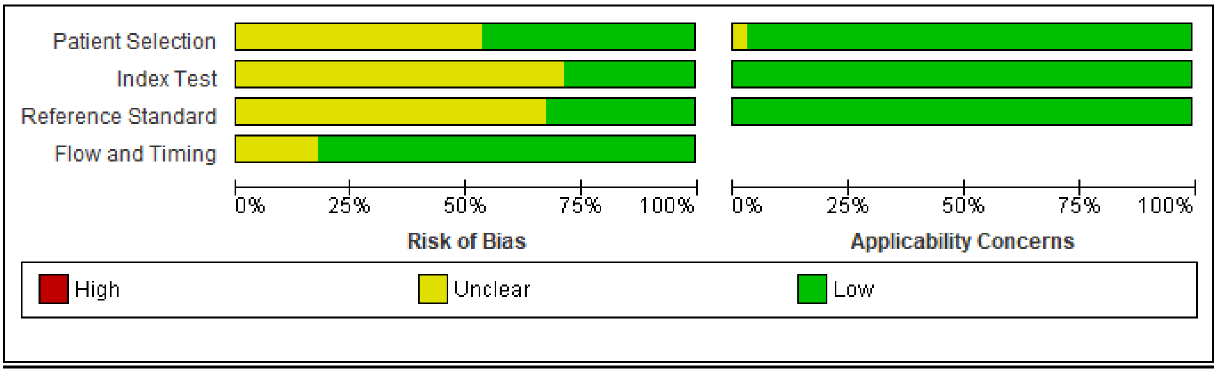

3.2: Risk of bias and applicability concerns summary: review author’s judgements about each domain for each included study

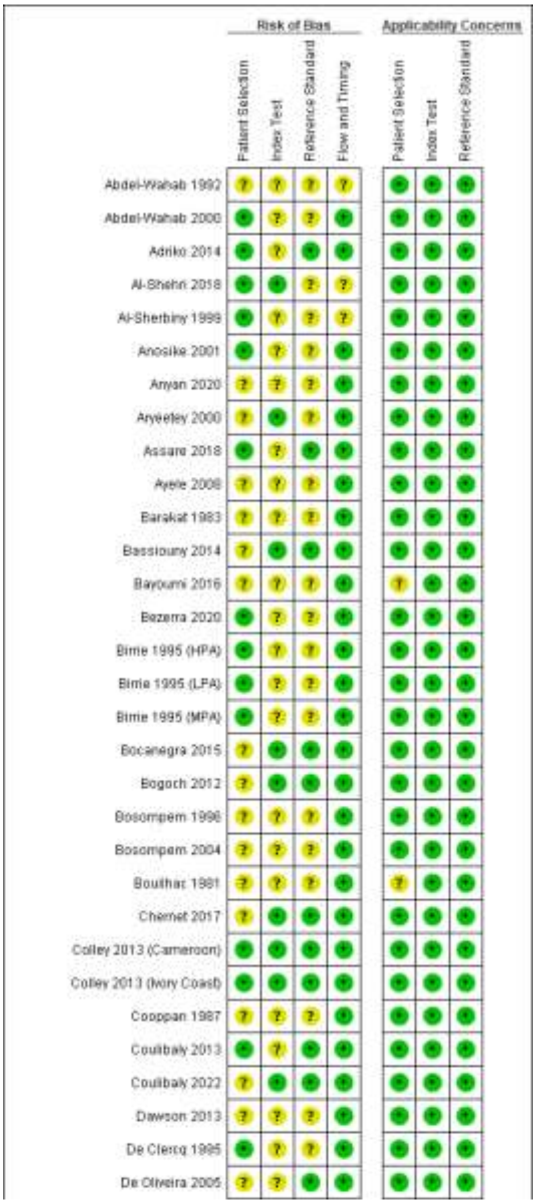

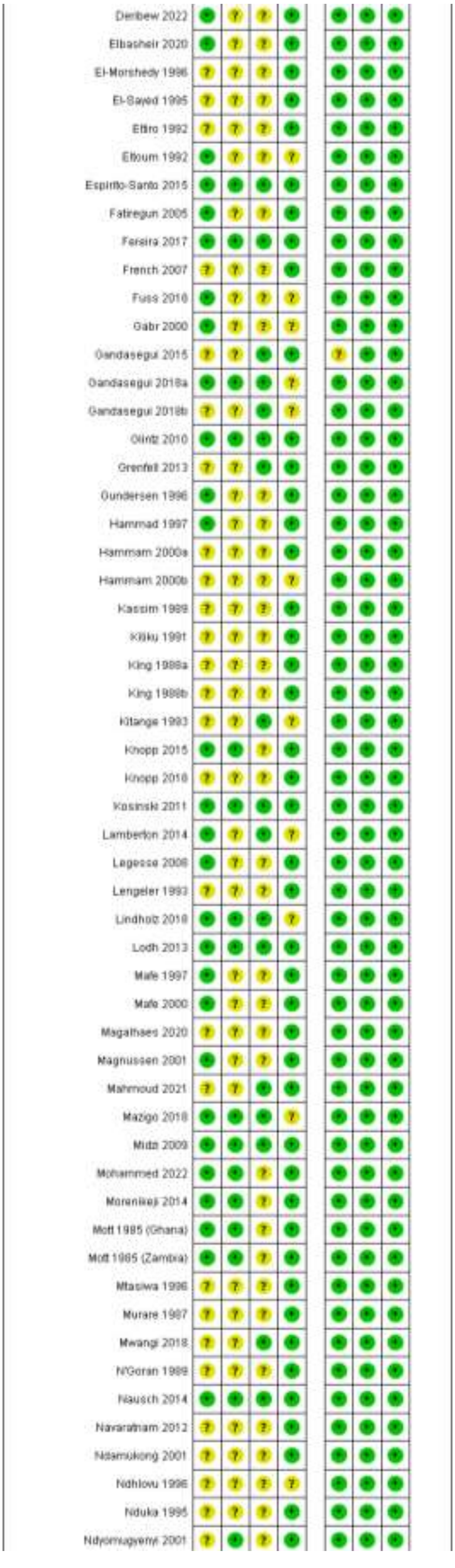

Deribew 2022
Elbasheir 2020
El-Morshedy 1996
El-Sayed 1995
Etiro 1992
Etoum 1992
Espirito-Santo 2015
Fatiregun 2005
Ferreira 2017
French 2007
Fuss 2016
Gabr 2000
Gandasegui 2015
Gandasegui 2018a
Gandasegui 2018b
Glinz 2010
Grenfell 2013
Gundersen 1996
Hammad 1997
Hammam 2000a
Hammam 2000b
Kassim 1989
Kiliku 1991
King 1988a
King 1988b
Kitange 1993
Knopp 2015
Knopp 2016
Kosinski 2011
Lamberton 2014
Legesse 2006
Lengeler 1993
Lindholz 2018
Lodh 2013
Mafe 1997
Mafe 2000
Magalhaes 2020
Magnussen 2001
Mahmoud 2021
Mazigo 2018
Midzi 2009
Mohammed 2022
Morenikeji 2014
Mott 1985 (Ghana)
Mott 1985 (Zambia)
Mtasiwa 1996
Murare 1987
Mwangi 2018
N'Goran 1989
Nausch 2014
Navaratnam 2012
Ndamukong 2001
Ndhlovu 1996
Nduka 1995
Ndyomugyenyi 2001

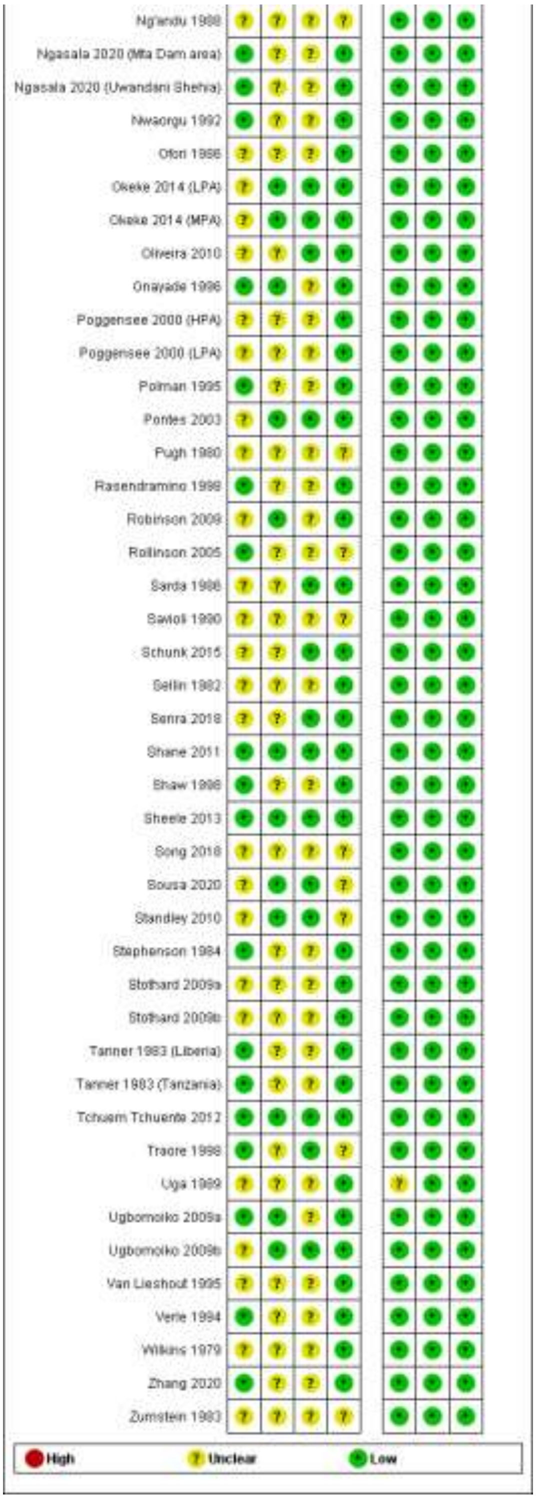

Abbreviations: LPA=low prevalence area, MPA=medium prevalence area, HPA=high prevalence area.

4: Additional figures and tables

4.1: Prevalence, sensitivity (Se) and specificity (Sp) for diagnostic tests investigated in one single study

Test comparison	Number of participants	Prevalence (%)	Sp (%) (95% CI)	Se (%) (95% CI)
Index test vs KK				
CCA1 cassette vs single KK	217	6.0	66 (59-73)	84 (52-98)
CCA1 cassette vs 16KK	217	14.0	71 (64-77)	81 (63-93)
CCA2 vs duplicate KK[49]	100	8.0	96 (89-99)	63 (24-91)
CCA2 vs quadruple KK[49]	100	11.0	96 (89-99)	45 (17-77)
FLOTAC (fresh) vs triplicate KK[50]	112	64.3	78 (62-89)	71 (59-81)
FLOTAC (stored for 10 days) vs triplicate KK[50]	112	64.3	70 (53-83)	96 (88-99)
FLOTAC (stored for 30 days) vs triplicate KK[50]	112	64.3	65 (48-79)	99 (93-100)
Sm DNA PCR vs duplicate KK[51]	89	50.6	23 (11-38)	100 (92-100)
IgM Elisa vs triplicate KK[52]	137	36.5	98 (92-1.00)	98 (89-100)
Helmintex vs duplicate KK[53]	461	11.9	67 (62-72)	98 (90-100)
COPT vs duplicate KK[54]	572	0.9	96 (94-97)	80 (28-99)
PCR-Elisa platform v1 vs KK[55]	206	18.4	85 (79-90)	97 (86-100)
PCR-Elisa platform v2 vs KK[55]	206	18.4	91 (86-95)	97 (86-100)
Index test vs urine filtration				
SmCTF-RDT vs urine filtration[56]	117	5.1	33 (25-43)	67 (22-96)
IHA vs urine filtration[57]	145	61.0	72 (58-83)	74 (64-83)
Anti IgG RDT Sh vs urine filtration[58]	160	51.3	10 (05-19)	46 (35-58)
Colorimetric test vs urine filtration[46]	1279	61.9	75 (79-78)	52 (49-56)
DDIA vs urine filtration[57]	146	61.0	61 (48-74)	60 (49-70)
SchistoScope vs urine filtration[59]	170	20.6	93 (88-97)	86 (70-95)
IMB based-Elisa vs urine filtration[60]	290	13.4	95 (91-97)	95 (83-99)
Index test vs index test				
Helmintex vs RT-PCR[61]	176	59.1	92 (83-97)	68 (48-77)
CCA1 vs RT-PCR[61]	196	55.1	73 (62-82)	65 (55-74)

CCA1 vs Helmintex[62]	214	24.8	71 (64-78)	57 (42-70)
IgG SEA Elisa vs CCA1[63]	258	57.0	53 (43-63)	97 (92-99)
PCR vs CCA1[63]	258	57.0	56 (46-65)	85 (78-90)

4.2: Forest plot - Sensitivity and specificity of CCA1 versus

A) Single, duplicate, quadruple, sextuple or 16 KK

CCA1 cassette vs single KK

Study	TP	FP	FN	TN	Sensitivity (95% CI)	Specificity (95% CI)
Sousa 2020	10	69	2	136	0.83 [0.52, 0.98]	0.66 [0.59, 0.73]

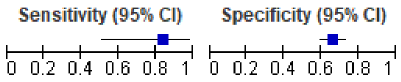

CCA1 cassette vs duplicate KK

Study	TP	FP	FN	TN	Sensitivity (95% CI)	Specificity (95% CI)
Adriko 2014	6	42	2	49	0.75 [0.35, 0.97]	0.54 [0.43, 0.64]
Al-Shehri 2018	113	34	1	110	0.99 [0.95, 1.00]	0.76 [0.69, 0.83]
Elbasheir 2020	168	46	7	268	0.96 [0.92, 0.98]	0.85 [0.81, 0.89]
Fereira 2017	11	71	7	211	0.61 [0.36, 0.83]	0.75 [0.69, 0.80]
Fuss 2018	249	34	4	10	0.98 [0.96, 1.00]	0.23 [0.11, 0.38]
Legesse 2008	60	60	18	46	0.77 [0.66, 0.86]	0.43 [0.34, 0.53]
Lindholz 2018	47	283	8	123	0.85 [0.73, 0.94]	0.30 [0.26, 0.35]
Lodh 2013	45	8	26	10	0.63 [0.51, 0.75]	0.56 [0.31, 0.78]
Navaratnam 2012	149	193	34	220	0.81 [0.75, 0.87]	0.53 [0.48, 0.58]
Polman 1995	341	24	43	14	0.89 [0.85, 0.92]	0.37 [0.22, 0.54]
Polman 1995	327	29	57	9	0.85 [0.81, 0.89]	0.24 [0.11, 0.40]
Sousa 2020	15	64	2	136	0.88 [0.64, 0.99]	0.68 [0.61, 0.74]
Standley 2010	103	17	14	37	0.88 [0.81, 0.93]	0.69 [0.54, 0.80]
Standley 2010	116	44	1	10	0.99 [0.95, 1.00]	0.19 [0.09, 0.31]
Tchuem Tchuente 2012	11	71	7	211	0.61 [0.36, 0.83]	0.75 [0.69, 0.80]
Van Lieshout 1995	21	10	37	136	0.36 [0.24, 0.50]	0.93 [0.88, 0.97]
Van Lieshout 1995	36	23	22	123	0.62 [0.48, 0.74]	0.84 [0.77, 0.90]

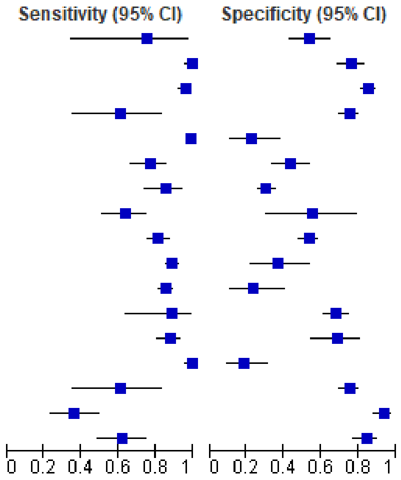

CCA1 cassette vs quadruple KK

Study	TP	FP	FN	TN	Sensitivity (95% CI)	Specificity (95% CI)
Adriko 2014	8	40	3	49	0.73 [0.39, 0.94]	0.55 [0.44, 0.66]
Chernet 2017	21	22	2	62	0.91 [0.72, 0.99]	0.74 [0.63, 0.83]
Colley 2013 (Cameroon)	247	208	27	231	0.90 [0.86, 0.93]	0.53 [0.48, 0.57]
Colley 2013 (Ivory Coast)	278	42	38	249	0.88 [0.84, 0.91]	0.86 [0.81, 0.89]
Coulibaly 2013	18	43	6	49	0.75 [0.53, 0.90]	0.53 [0.43, 0.64]
Dawson 2013	9	11	4	18	0.69 [0.39, 0.91]	0.62 [0.42, 0.79]
Dawson 2013	23	8	1	8	0.96 [0.79, 1.00]	0.50 [0.25, 0.75]
Fereira 2017	11	71	10	216	0.52 [0.30, 0.74]	0.75 [0.70, 0.80]
Mazigo 2018	233	132	9	45	0.96 [0.93, 0.98]	0.25 [0.19, 0.33]
Shane 2011	231	664	35	833	0.87 [0.82, 0.91]	0.56 [0.53, 0.58]

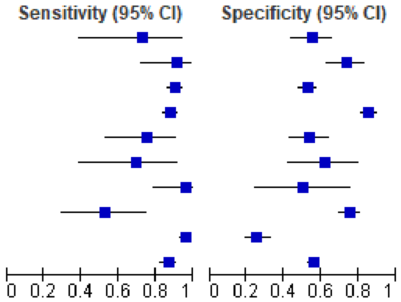

CCA1 cassette vs sextuple KK

Study	TP	FP	FN	TN	Sensitivity (95% CI)	Specificity (95% CI)
Adriko 2014	10	38	3	47	0.77 [0.46, 0.95]	0.55 [0.44, 0.66]
Assare 2018	38	187	7	449	0.84 [0.71, 0.94]	0.71 [0.67, 0.74]
Bezerra 2020	46	11	16	54	0.74 [0.62, 0.84]	0.83 [0.72, 0.91]
Fereira 2017	10	72	8	224	0.56 [0.31, 0.78]	0.76 [0.70, 0.80]
Lamberton 2014	66	1	6	3	0.92 [0.83, 0.97]	0.75 [0.19, 0.99]
Shane 2011	176	88	11	129	0.94 [0.90, 0.97]	0.59 [0.53, 0.66]
Tchuem Tchuente 2012	322	94	59	150	0.85 [0.80, 0.88]	0.61 [0.55, 0.68]

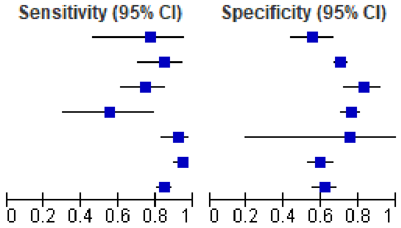

CCA1 cassette vs 16KK

Study	TP	FP	FN	TN	Sensitivity (95% CI)	Specificity (95% CI)
Sousa 2020	25	54	6	132	0.81 [0.63, 0.93]	0.71 [0.64, 0.77]

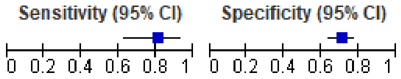

B) Urine microscopy

Study	TP	FP	FN	TN	Sensitivity (95% CI)	Specificity (95% CI)
Al-Sherbiny 1999	52	90	15	213	0.78 [0.66, 0.87]	0.70 [0.65, 0.75]
Ayele 2008	51	39	47	69	0.52 [0.42, 0.62]	0.64 [0.54, 0.73]
Midzi 2009	84	88	23	70	0.79 [0.70, 0.86]	0.44 [0.36, 0.52]
Stothard 2009a	4	2	42	102	0.09 [0.02, 0.21]	0.98 [0.93, 1.00]

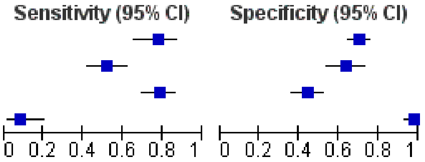

Abbreviations: TP=true positive, FP=false positive, FN=false negative, TN=true negative, CI=confidence interval, KK=Kato-Katz thick smear, CCA1=Circulating cathodic antigen urine cassette assay (version 1)

4.3: Pooled Se and Sp in models incorporating conditional dependence

Test comparison	# comp.	Proportion of maximum conditional dependence							
		0.05		0.10		0.20		0.30	
		Sp (95% CrI)	Se (95% CrI)	Sp (95% CrI)	Se (95% CrI)	Sp (95% CrI)	Se (95% CrI)	Sp (95% CrI)	Se (95% CrI)
CCA1 vs duplicate KK	17	0.75 (0.55-0.90)	0.94 (0.85-0.99)	0.72 (0.55-0.87)	0.95 (0.87-0.99)	0.71 (0.54-0.86)	0.94 (0.84-0.99)	0.70 (0.53-0.85)	0.93 (0.84-0.99)
CCA1 vs quadruple KK	10	0.72 (0.56-0.87)	0.96 (0.89-1.00)	0.71 (0.57-0.85)	0.95 (0.89-0.99)	0.70 (0.55-0.84)	0.95 (0.87-0.99)	0.68 (0.54-0.83)	0.93 (0.84-0.99)
CCA1 vs KK (all)	36	0.74 (0.64-0.83)	0.95 (0.90-0.98)	0.74 (0.64-0.84)	0.94 (0.90-0.98)	0.72 (0.62-0.82)	0.93 (0.87-0.98)	0.70 (0.59-0.80)	0.92 (0.84-0.97)
Proteinuria vs urine microscopy	42	0.93 (0.87-0.97)	0.71 (0.59-0.80)	0.91 (0.82-0.98)	0.66 (0.53-0.77)	0.92 (0.83-0.98)	0.68 (0.55-0.80)	0.88 (0.79-0.94)	0.61 (0.48-0.73)
Haematuria vs urine microscopy	75	0.95 (0.92-0.98)	0.84 (0.77-0.89)	0.96 (0.92-0.98)	0.81 (0.72-0.87)	0.95 (0.92-0.98)	0.82 (0.76-0.88)	0.94 (0.90-0.97)	0.77 (0.70-0.83)

Abbreviations: Se=sensitivity, Sp=specificity, CrI=credible interval, #comp=number of comparisons, KK=Kato-Katz thick smear, CCA1= Circulating cathodic antigen urine cassette assay (version 1)

4.4: Forest plot – Sensitivity and specificity of CAA versus

A) <u>Duplicate or quadruple KK</u>

CAA vs duplicate KK

Study	TP	FP	FN	TN	Sensitivity (95% CI)	Specificity (95% CI)
Polman 1995	344	24	40	14	0.90 [0.86, 0.92]	0.37 [0.22, 0.54]
Van Lieshout 1995	6	1	52	145	0.10 [0.04, 0.21]	0.99 [0.96, 1.00]
Van Lieshout 1995	27	20	31	126	0.47 [0.33, 0.60]	0.86 [0.80, 0.91]

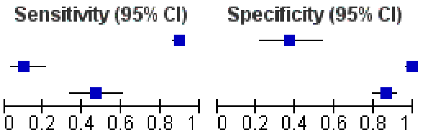

B) <u>Urine microscopy</u>

Study	TP	FP	FN	TN	Sensitivity (95% CI)	Specificity (95% CI)
Al-Sherbiny 1999	11	18	56	285	0.16 [0.08, 0.27]	0.94 [0.91, 0.96]
De Clercq 1995	199	82	55	105	0.78 [0.73, 0.83]	0.56 [0.49, 0.63]
El-Morshedy 1996	117	0	30	110	0.80 [0.72, 0.86]	1.00 [0.97, 1.00]
Ndhlovu 1996	93	63	3	20	0.97 [0.91, 0.99]	0.24 [0.15, 0.35]

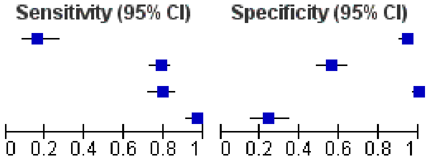

<u>Abbreviations:</u> TP=true positive, FP=false positive, FN=false negative, TN=true negative, CI=confidence interval, KK=Kato-Katz smear, CAA= Circulating anodic antigen urine serum/urine assay

4.5: Hierarchical summary Receiver Operating Characteristic Plot with summary points

A) <u>CAA vs duplicate KK</u>

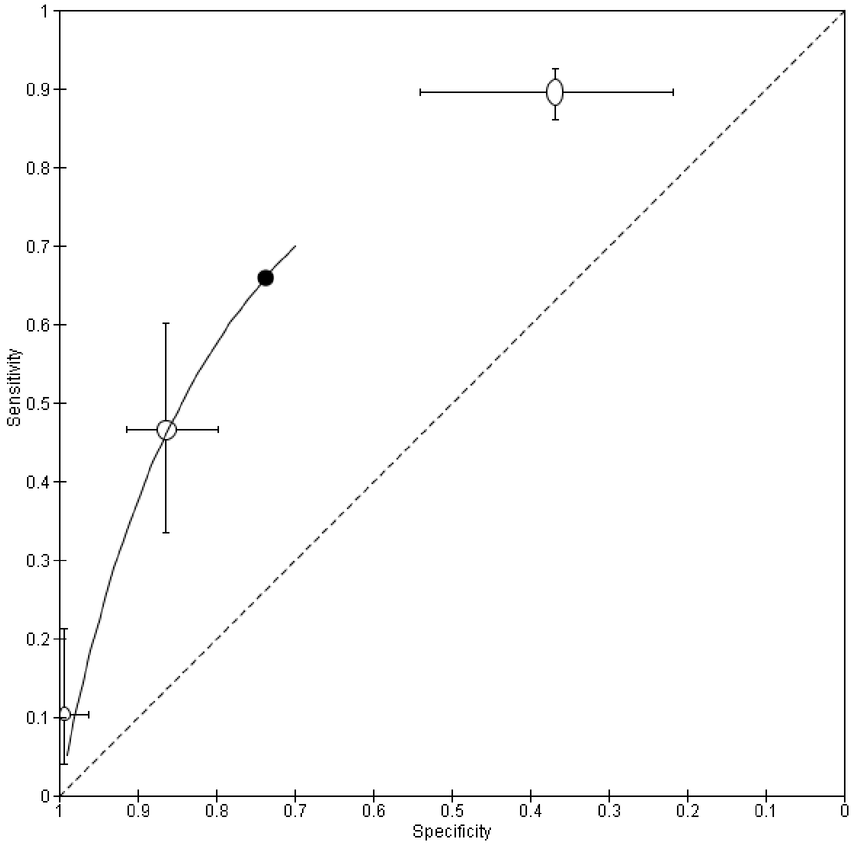

B) <u>CAA vs urine microscopy</u>

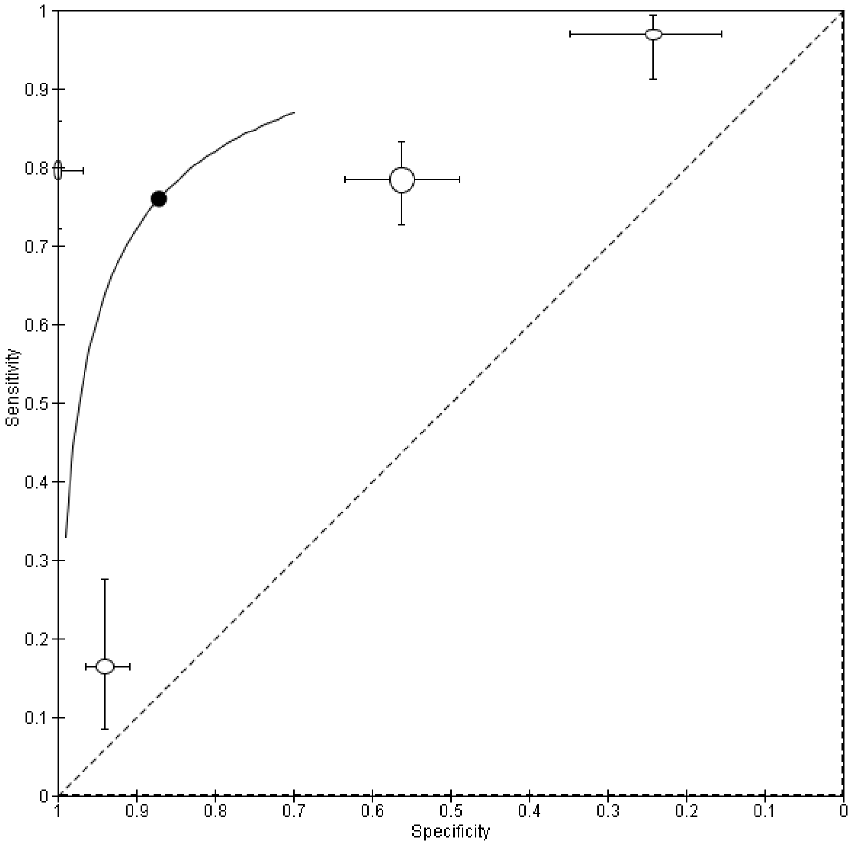

<u>Abbreviations</u>: KK=Kato-Katz, CAA= Circulating anodic antigen urine cassette assay

<u>Legend</u>: Each unfilled symbol represents the individual study estimates pair (Se, Sp), larger symbols reflect a higher sample size. The solid circles are the meta-analytic estimates across the studies included in the meta-analysis and the solid lines the summary HSROC curves.

4.6: Forest plot – Sensitivity and specificity

A) <u>Proteinuria reagent strip vs urine microscopy</u>

Study	TP	FP	FN	TN	Sensitivity (95% CI)	Specificity (95% CI)
Abdel-Wahab 1992	32	31	110	249	0.23 [0.16, 0.30]	0.89 [0.85, 0.92]
Abdel-Wahab 2000	97	81	602	4338	0.14 [0.11, 0.17]	0.98 [0.98, 0.99]
Aryeetey 2000	610	842	1003	107	0.38 [0.35, 0.40]	0.11 [0.09, 0.13]
Bogoch 2012	8	53	11	208	0.42 [0.20, 0.67]	0.80 [0.74, 0.84]
Bosompem 1996	44	10	65	110	0.40 [0.31, 0.50]	0.92 [0.85, 0.96]
Bosompem 2004	26	17	59	39	0.31 [0.21, 0.42]	0.70 [0.56, 0.81]
Cooppan 1987	616	112	145	68	0.81 [0.78, 0.84]	0.38 [0.31, 0.45]
Gabr 2000	185	293	889	10540	0.17 [0.15, 0.20]	0.97 [0.97, 0.98]
Gundersen 1996	43	163	8	46	0.84 [0.71, 0.93]	0.22 [0.17, 0.28]
Hammad 1997	662	3081	410	7817	0.62 [0.59, 0.65]	0.72 [0.71, 0.73]
Hammam 2000a	297	1174	369	10487	0.45 [0.41, 0.48]	0.90 [0.89, 0.90]
Hammam 2000b	155	605	433	8362	0.26 [0.23, 0.30]	0.93 [0.93, 0.94]
Kassim 1989	96	98	24	704	0.80 [0.72, 0.87]	0.88 [0.85, 0.90]
Kiliku 1991	197	67	56	106	0.78 [0.72, 0.83]	0.61 [0.54, 0.69]
King 1988a	1343	118	478	670	0.74 [0.72, 0.76]	0.85 [0.82, 0.87]
Kitange 1993	27	4	56	166	0.33 [0.23, 0.44]	0.98 [0.94, 0.99]
Mahmoud 2021	29	129	10	122	0.74 [0.58, 0.87]	0.49 [0.42, 0.55]
Mott 1985 (Ghana)	334	99	38	47	0.90 [0.86, 0.93]	0.32 [0.25, 0.40]
Mott 1985 (Zambia)	428	25	75	123	0.85 [0.82, 0.88]	0.83 [0.76, 0.89]
Murare 1987	140	25	22	45	0.86 [0.80, 0.91]	0.64 [0.52, 0.75]
Ndamukong 2001	155	17	31	144	0.83 [0.77, 0.88]	0.89 [0.84, 0.94]
Ng'andu 1988	90	58	79	185	0.53 [0.45, 0.61]	0.76 [0.70, 0.81]
Nwaorgu 1992	537	85	43	352	0.93 [0.90, 0.95]	0.81 [0.77, 0.84]
Ofori 1986	42	13	22	41	0.66 [0.53, 0.77]	0.76 [0.62, 0.87]
Okeke 2014 (LPA)	8	9	7	272	0.53 [0.27, 0.79]	0.97 [0.94, 0.99]
Okeke 2014 (MPA)	15	18	34	117	0.31 [0.18, 0.45]	0.87 [0.80, 0.92]
Onayade 1996	53	1	40	11	0.57 [0.46, 0.67]	0.92 [0.62, 1.00]
Poggensee 2000 (HPA)	8	6	59	55	0.12 [0.05, 0.22]	0.90 [0.80, 0.96]
Poggensee 2000 (LPA)	1	14	6	154	0.14 [0.00, 0.58]	0.92 [0.86, 0.95]
Pugh 1980	508	887	422	3550	0.55 [0.51, 0.58]	0.80 [0.79, 0.81]
Rasendramino 1998	316	20	104	107	0.75 [0.71, 0.79]	0.84 [0.77, 0.90]
Sarda 1986	234	173	94	799	0.71 [0.66, 0.76]	0.82 [0.80, 0.85]
Sellin 1982	376	227	162	397	0.70 [0.66, 0.74]	0.64 [0.60, 0.67]
Stephenson 1984	113	11	58	177	0.66 [0.58, 0.73]	0.94 [0.90, 0.97]
Tanner 1983 (Liberia)	108	10	81	68	0.57 [0.50, 0.64]	0.87 [0.78, 0.94]
Tanner 1983 (Tanzania)	136	68	26	318	0.84 [0.77, 0.89]	0.82 [0.78, 0.86]
Traore 1998	340	84	235	382	0.59 [0.55, 0.63]	0.82 [0.78, 0.85]
Ugbomoiko 2009a	207	12	149	202	0.58 [0.53, 0.63]	0.94 [0.90, 0.97]
Ugbomoiko 2009a	602	9	147	699	0.80 [0.77, 0.83]	0.99 [0.98, 0.99]
Ugbomoiko 2009b	121	45	106	175	0.53 [0.47, 0.60]	0.80 [0.74, 0.85]
Verle 1994	168	21	138	25	0.55 [0.49, 0.61]	0.54 [0.39, 0.69]
Wilkins 1979	701	251	377	615	0.65 [0.62, 0.68]	0.71 [0.68, 0.74]

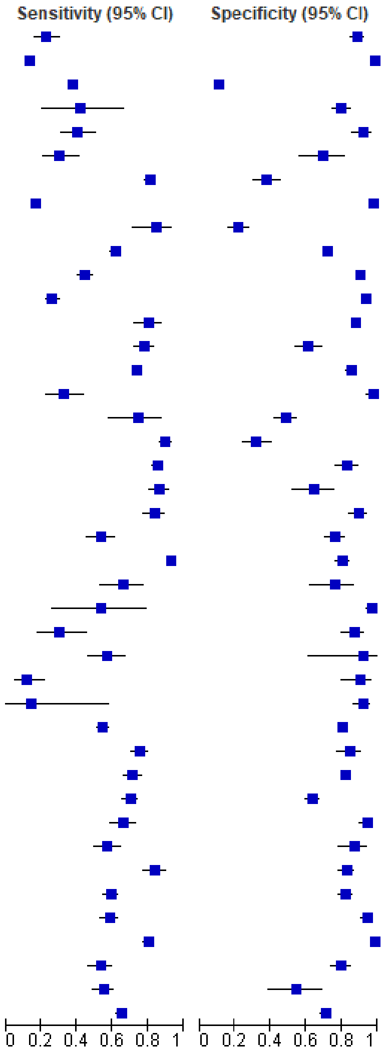

B) Haematuria reagent strip vs urine microscopy

Study	TP	FP	FN	TN	Sensitivity (95% CI)	Specificity (95% CI)
Abdel-Wahab 1992	80	102	62	178	0.56 [0.48, 0.65]	0.64 [0.58, 0.69]
Abdel-Wahab 2000	502	1032	196	3388	0.72 [0.68, 0.75]	0.77 [0.75, 0.78]
Anosike 2001	240	106	345	482	0.41 [0.37, 0.45]	0.82 [0.79, 0.85]
Aryeetey 2000	1117	335	919	191	0.55 [0.53, 0.57]	0.36 [0.32, 0.41]
Bassiouny 2014	78	34	48	536	0.62 [0.53, 0.70]	0.94 [0.92, 0.96]
Birrie 1995 (HPA)	44	22	25	135	0.64 [0.51, 0.75]	0.86 [0.80, 0.91]
Birrie 1995 (LPA)	4	6	1	144	0.80 [0.28, 0.99]	0.96 [0.91, 0.99]
Birrie 1995 (MPA)	20	17	6	81	0.77 [0.56, 0.91]	0.83 [0.74, 0.90]
Bocanegra 2015	749	95	31	408	0.96 [0.94, 0.97]	0.81 [0.77, 0.84]
Bogoch 2012	19	18	0	243	1.00 [0.82, 1.00]	0.93 [0.89, 0.96]
Bosompem 1996	83	8	26	112	0.76 [0.67, 0.84]	0.93 [0.87, 0.97]
Bosompem 2004	33	5	52	51	0.39 [0.28, 0.50]	0.91 [0.80, 0.97]
Cooppan 1987	632	21	129	159	0.83 [0.80, 0.86]	0.88 [0.83, 0.93]
Deribew 2022	142	122	1	906	0.99 [0.96, 1.00]	0.88 [0.86, 0.90]
El-Sayed 1995	9	176	12	440	0.43 [0.22, 0.66]	0.71 [0.68, 0.75]
Eltoum 1992	145	123	34	123	0.81 [0.74, 0.86]	0.50 [0.44, 0.56]
Fatiregun 2005	49	49	23	471	0.68 [0.56, 0.79]	0.91 [0.88, 0.93]
French 2007	219	45	41	1671	0.84 [0.79, 0.88]	0.97 [0.97, 0.98]
Gabr 2000	648	1829	426	9007	0.60 [0.57, 0.63]	0.83 [0.82, 0.84]
Gandasegui 2018a	62	21	25	64	0.71 [0.61, 0.80]	0.75 [0.65, 0.84]
Gundersen 1996	50	158	1	51	0.98 [0.90, 1.00]	0.24 [0.19, 0.31]
Hammad 1997	712	2408	360	8490	0.66 [0.64, 0.69]	0.78 [0.77, 0.79]
Hammam 2000a	409	2526	257	9134	0.61 [0.58, 0.65]	0.78 [0.78, 0.79]
Hammam 2000b	245	1464	343	7503	0.42 [0.38, 0.46]	0.84 [0.83, 0.84]
Kassim 1989	99	11	21	791	0.82 [0.75, 0.89]	0.99 [0.98, 0.99]
Kiliku 1991	159	14	94	159	0.63 [0.57, 0.69]	0.92 [0.87, 0.96]
King 1988a	1362	47	459	741	0.75 [0.73, 0.77]	0.94 [0.92, 0.96]
King 1988b	199	38	215	187	0.48 [0.43, 0.53]	0.83 [0.78, 0.88]
Kitange 1993	80	17	3	153	0.96 [0.90, 0.99]	0.90 [0.84, 0.94]
Knopp 2015	31	18	26	1125	0.54 [0.41, 0.68]	0.98 [0.98, 0.99]
Knopp 2018	1861	2695	751	52660	0.71 [0.69, 0.73]	0.95 [0.95, 0.95]
Kosinski 2011	52	13	70	120	0.43 [0.34, 0.52]	0.90 [0.84, 0.95]
Lengeler 1993	228	117	66	797	0.78 [0.72, 0.82]	0.87 [0.85, 0.89]
Mafe 1997	416	91	190	359	0.69 [0.65, 0.72]	0.80 [0.76, 0.83]
Mafe 2000	134	61	38	296	0.78 [0.71, 0.84]	0.83 [0.79, 0.87]
Magnussen 2001	107	3	33	27	0.76 [0.69, 0.83]	0.90 [0.73, 0.98]
Mahmoud 2021	18	44	21	207	0.46 [0.30, 0.63]	0.82 [0.77, 0.87]
Mohammed 2022	23	434	0	16002	1.00 [0.85, 1.00]	0.97 [0.97, 0.98]
Morenikeji 2014	69	11	209	198	0.25 [0.20, 0.30]	0.95 [0.91, 0.97]
Mott 1985 (Ghana)	267	20	121	154	0.69 [0.64, 0.73]	0.89 [0.83, 0.93]
Mott 1985 (Zambia)	382	9	74	191	0.84 [0.80, 0.87]	0.95 [0.92, 0.98]
Mtasiwa 1996	253	18	20	113	0.93 [0.89, 0.95]	0.86 [0.79, 0.92]
Murare 1987	126	12	36	58	0.78 [0.71, 0.84]	0.83 [0.72, 0.91]
N'Goran 1989	160	111	19	256	0.89 [0.84, 0.93]	0.70 [0.65, 0.74]
Ndamukong 2001	169	4	17	157	0.91 [0.86, 0.95]	0.98 [0.94, 0.99]
Nduka 1995	38	3	207	917	0.16 [0.11, 0.21]	1.00 [0.99, 1.00]
Ndyomugyenyi 2001	194	36	58	195	0.77 [0.71, 0.82]	0.84 [0.79, 0.89]
Ng'andu 1988	130	43	39	200	0.77 [0.70, 0.83]	0.82 [0.77, 0.87]
Ngasala 2020 (Mta Dam area)	18	80	6	249	0.75 [0.53, 0.90]	0.76 [0.71, 0.80]
Ngasala 2020 (Uwandani Shehia)	56	16	2	76	0.97 [0.88, 1.00]	0.83 [0.73, 0.90]
Nwaorgu 1992	427	149	53	388	0.89 [0.86, 0.92]	0.72 [0.68, 0.76]
Ofori 1986	45	0	19	54	0.70 [0.58, 0.81]	1.00 [0.93, 1.00]
Okeke 2014 (LPA)	11	8	4	273	0.73 [0.45, 0.92]	0.97 [0.94, 0.99]
Okeke 2014 (MPA)	21	17	28	118	0.43 [0.29, 0.58]	0.87 [0.81, 0.92]
Poggensee 2000 (HPA)	44	26	23	35	0.66 [0.53, 0.77]	0.57 [0.44, 0.70]
Poggensee 2000 (LPA)	4	48	3	120	0.57 [0.18, 0.90]	0.71 [0.64, 0.78]
Pugh 1980	415	444	515	3993	0.45 [0.41, 0.48]	0.90 [0.89, 0.91]
Rasendramino 1998	352	32	68	95	0.84 [0.80, 0.87]	0.75 [0.66, 0.82]
Robinson 2009	135	222	3	317	0.98 [0.94, 1.00]	0.59 [0.55, 0.63]
Rollinson 2005	125	16	26	113	0.83 [0.76, 0.88]	0.88 [0.81, 0.93]
Sarda 1986	275	54	53	918	0.84 [0.79, 0.88]	0.94 [0.93, 0.96]
Savioli 1990	113	38	64	305	0.64 [0.56, 0.71]	0.89 [0.85, 0.92]
Sellin 1982	463	356	75	268	0.86 [0.83, 0.89]	0.43 [0.39, 0.47]
Shaw 1998	216	105	121	415	0.64 [0.59, 0.69]	0.80 [0.76, 0.83]
Stephenson 1984	150	6	21	182	0.88 [0.82, 0.92]	0.97 [0.93, 0.99]
Stothard 2009b	42	9	1	14	0.98 [0.88, 1.00]	0.61 [0.39, 0.80]
Tanner 1983 (Liberia)	129	10	60	68	0.68 [0.61, 0.75]	0.87 [0.78, 0.94]
Tanner 1983 (Tanzania)	139	42	23	344	0.86 [0.79, 0.91]	0.89 [0.86, 0.92]
Traore 1998	420	74	155	392	0.73 [0.69, 0.77]	0.84 [0.80, 0.87]
Ugbomoiko 2009a	598	78	151	630	0.80 [0.77, 0.83]	0.89 [0.86, 0.91]
Ugbomoiko 2009a	333	25	23	189	0.94 [0.90, 0.96]	0.88 [0.83, 0.92]
Ugbomoiko 2009b	155	37	72	183	0.68 [0.62, 0.74]	0.83 [0.78, 0.88]
Verle 1994	205	15	101	31	0.67 [0.61, 0.72]	0.67 [0.52, 0.80]
Wilkins 1979	585	95	493	771	0.54 [0.51, 0.57]	0.89 [0.87, 0.91]
Zumstein 1983	134	15	48	199	0.74 [0.67, 0.80]	0.93 [0.89, 0.96]

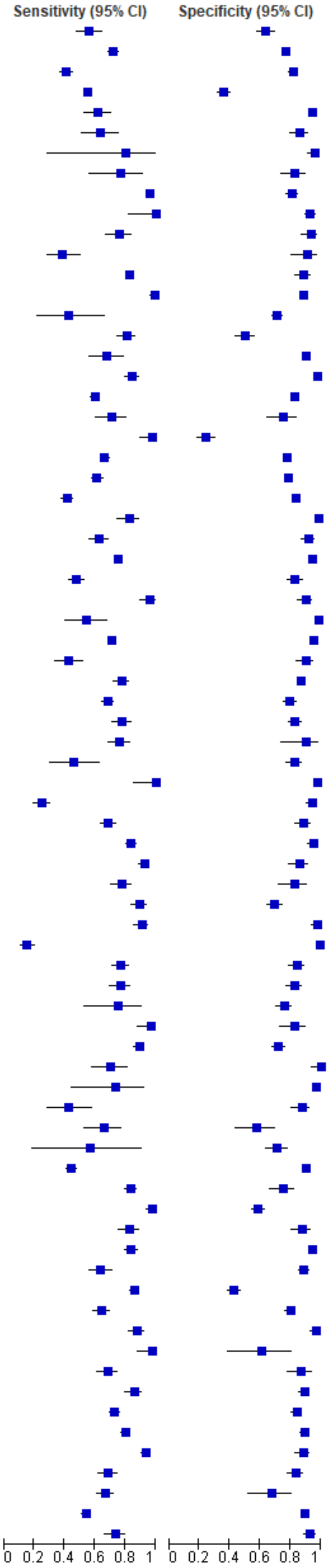

Abbreviations: TP=true positive, FP=false positive, FN=false negative, TN=true negative, CI=confidence interval, LPA=low prevalence area, MPA=medium prevalence area, HPA=high prevalence area

4.7: Leukocyturia vs urine microscopy

A) Forest plot - Sensitivity and specificity

Study	TP	FP	FN	TN	Sensitivity (95% CI)	Specificity (95% CI)
Abdel-Wahab 1992	46	20	96	260	0.32 [0.25, 0.41]	0.93 [0.89, 0.96]
Gundersen 1996	37	160	14	49	0.73 [0.58, 0.84]	0.23 [0.18, 0.30]
Poggensee 2000 (HPA)	38	32	29	29	0.57 [0.44, 0.69]	0.48 [0.35, 0.61]
Poggensee 2000 (LPA)	4	92	3	76	0.57 [0.18, 0.90]	0.45 [0.38, 0.53]
Rasendramino 1998	238	30	182	97	0.57 [0.52, 0.61]	0.76 [0.68, 0.83]

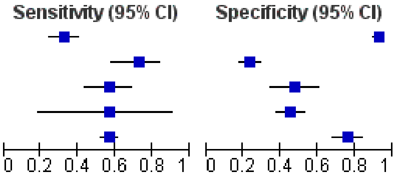

Abbreviations: TP=true positive, FP=false positive, FN=false negative, TN=true negative, CI=confidence interval, LPA=low prevalence area, HPA=high prevalence area

B) Hierarchical summary Receiver Operating Characteristic Plot with summary point

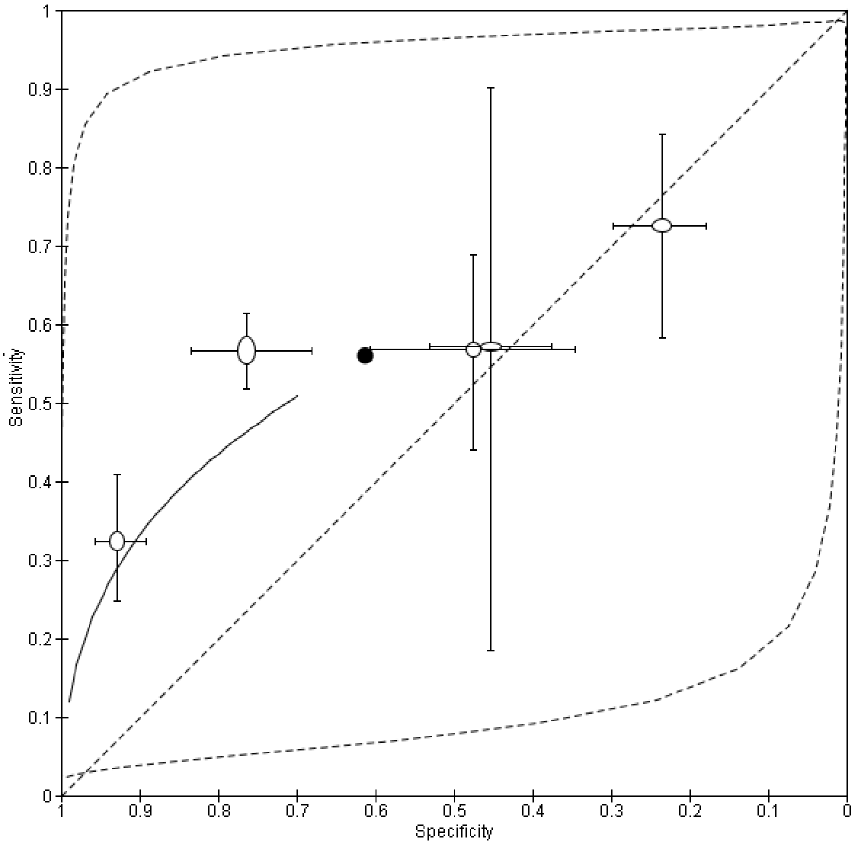

Legend: Each unfilled circle represents the individual study estimates pair (Se, Sp), larger circles reflect a higher sample size. The solid circle is the meta-analytic estimate across the studies included in the meta-analysis, the solid line the summary HSROC curve and the dotted-curve the 95% prediction region for a future study

4.8: SmCTF-RDT vs quadruple Kato-Katz smears

A) Forest plot - Sensitivity and specificity

Study	TP	FP	FN	TN	Sensitivity (95% CI)	Specificity (95% CI)
Coulibaly 2013	18	62	6	32	0.75 [0.53, 0.90]	0.34 [0.25, 0.45]
Dawson 2013	21	14	3	2	0.88 [0.68, 0.97]	0.13 [0.02, 0.38]
Dawson 2013	7	17	6	12	0.54 [0.25, 0.81]	0.41 [0.24, 0.61]
Nausch 2014	53	23	0	15	1.00 [0.93, 1.00]	0.39 [0.24, 0.57]

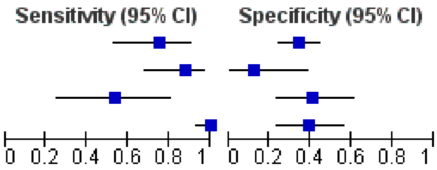

Abbreviations: TP=true positive, FP=false positive, FN=false negative, TN=true negative, CI=confidence interval

B) Hierarchical summary Receiver Operating Characteristic Plot with summary point

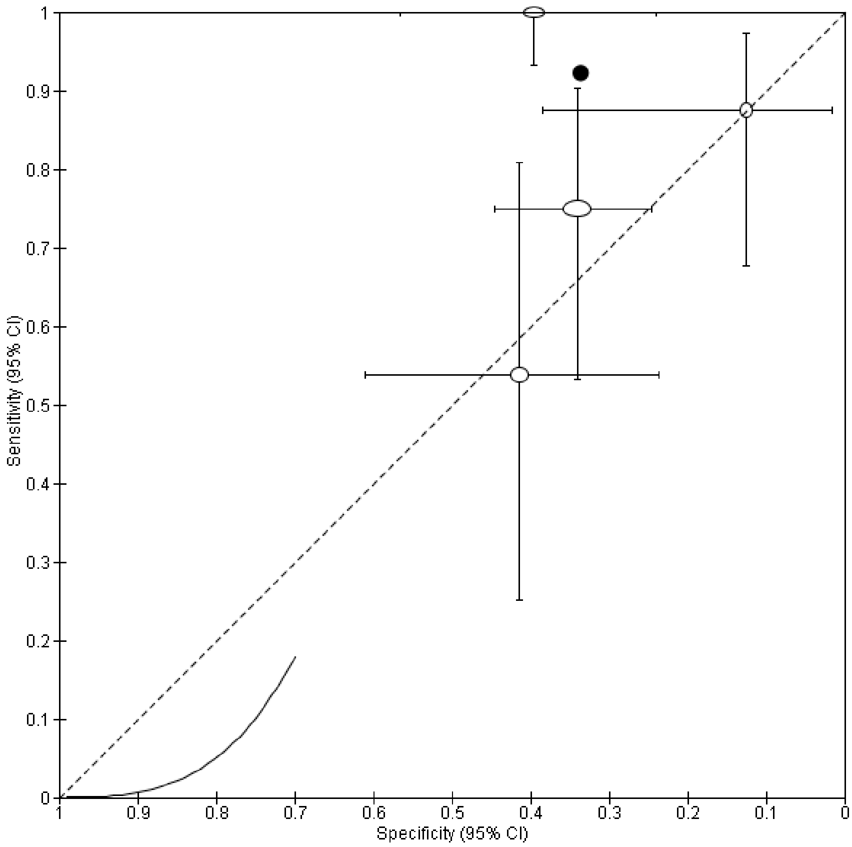

Legend: Each unfilled circle represents the individual study estimates pair (Se, Sp), larger circles reflect a higher sample size. The solid circle is the meta-analytic estimate across the studies included in the meta-analysis and the solid line the summary HSROC curve.

4.9: Forest plots - Sensitivity and specificity

A) IgG Elisa vs triplicate KK

Study	TP	FP	FN	TN	Sensitivity (95% CI)	Specificity (95% CI)
Barakat 1983	177	25	15	290	0.92 [0.87, 0.96]	0.92 [0.89, 0.95]
De Oliveira 2005	48	1	2	86	0.96 [0.86, 1.00]	0.99 [0.94, 1.00]
Eltiro 1992	163	23	4	10	0.98 [0.94, 0.99]	0.30 [0.16, 0.49]

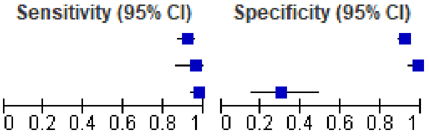

B) AWE-SEA Elisa vs KK

Study	TP	FP	FN	TN	Sensitivity (95% CI)	Specificity (95% CI)
Chernet 2017	22	32	1	52	0.96 [0.78, 1.00]	0.62 [0.51, 0.72]
Grenfell 2013	17	4	3	16	0.85 [0.62, 0.97]	0.80 [0.56, 0.94]

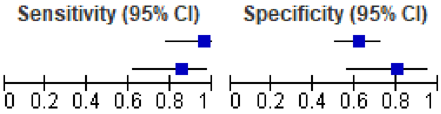

C) SWAP Elisa vs KK

Study	TP	FP	FN	TN	Sensitivity (95% CI)	Specificity (95% CI)
Grenfell 2013	18	2	2	18	0.90 [0.68, 0.99]	0.90 [0.68, 0.99]
Shane 2011	172	126	15	169	0.92 [0.87, 0.95]	0.57 [0.51, 0.63]

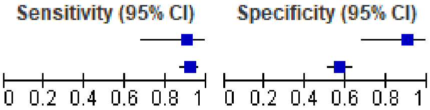

D) IgG SEA Elisa vs urine microscopy

Study	TP	FP	FN	TN	Sensitivity (95% CI)	Specificity (95% CI)
Bouilhac 1981	42	5	12	95	0.78 [0.64, 0.88]	0.95 [0.89, 0.98]
Mahmoud 2021	34	19	5	232	0.87 [0.73, 0.96]	0.92 [0.88, 0.95]
Song 2018	55	64	3	27	0.95 [0.86, 0.99]	0.30 [0.21, 0.40]
Stothard 2009a	41	31	5	73	0.89 [0.76, 0.96]	0.70 [0.60, 0.79]
Uga 1989	36	2	3	9	0.92 [0.79, 0.98]	0.82 [0.48, 0.98]

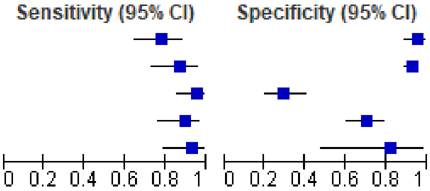

E) PCR vs KK

Study	TP	FP	FN	TN	Sensitivity (95% CI)	Specificity (95% CI)
Anyan 2020	21	58	0	84	1.00 [0.84, 1.00]	0.59 [0.51, 0.67]
Anyan 2020	45	8	0	38	1.00 [0.92, 1.00]	0.83 [0.69, 0.92]
Oliveira 2010	25	7	9	61	0.74 [0.56, 0.87]	0.90 [0.80, 0.96]
Oliveira 2010	28	12	6	56	0.82 [0.65, 0.93]	0.82 [0.71, 0.91]
Pontes 2003	58	16	2	118	0.97 [0.88, 1.00]	0.88 [0.81, 0.93]

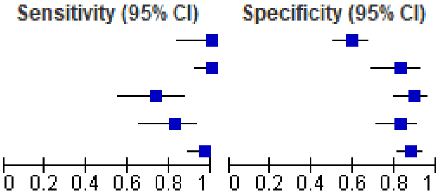

F) RT-PCR vs KK

Study	TP	FP	FN	TN	Sensitivity (95% CI)	Specificity (95% CI)
Fuss 2018	245	31	8	13	0.97 [0.94, 0.99]	0.30 [0.17, 0.45]
Magalhaes 2020	61	42	3	92	0.95 [0.87, 0.99]	0.69 [0.60, 0.76]
Magalhaes 2020	41	73	0	92	1.00 [0.91, 1.00]	0.56 [0.48, 0.63]
Schunk 2015	32	0	3	20	0.91 [0.77, 0.98]	1.00 [0.83, 1.00]
Schunk 2015	33	0	2	20	0.94 [0.81, 0.99]	1.00 [0.83, 1.00]

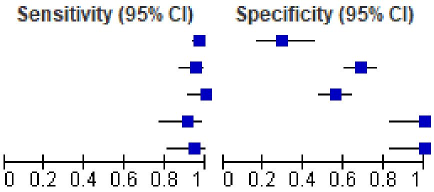

G) LAMP vs KK or urine microscopy

LAMP vs Urine Microscopy

Study	TP	FP	FN	TN	Sensitivity (95% CI)	Specificity (95% CI)
Bayoumi 2016	31	14	0	24	1.00 [0.89, 1.00]	0.63 [0.46, 0.78]
Gandasegui 2015	18	8	7	61	0.72 [0.51, 0.88]	0.88 [0.78, 0.95]
Gandasegui 2018a	75	52	12	33	0.86 [0.77, 0.93]	0.39 [0.28, 0.50]

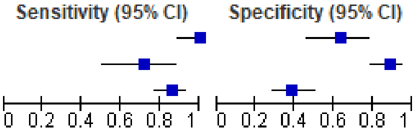

LAMP vs KK

Study	TP	FP	FN	TN	Sensitivity (95% CI)	Specificity (95% CI)
Gandasegui 2018b	12	37	1	112	0.92 [0.64, 1.00]	0.75 [0.67, 0.82]
Mwangi 2018	171	0	5	207	0.97 [0.93, 0.99]	1.00 [0.98, 1.00]

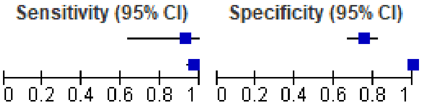

Abbreviations: TP=true positive, FP=false positive, FN=false negative, TN=true negative, CI=confidence interval, AWE=adult worm extract, KK=Kato-Katz thick smear, LAMP=loop-mediated isothermal amplification, PCR=polymerase chain reaction, RT=real-time, SEA=soluble egg antigen, SWAP=soluble adult worm antigen preparation.

4.10: Hierarchical summary Receiver Operating Characteristic Plots with summary point

A) IgG Elisa vs triplicate KK

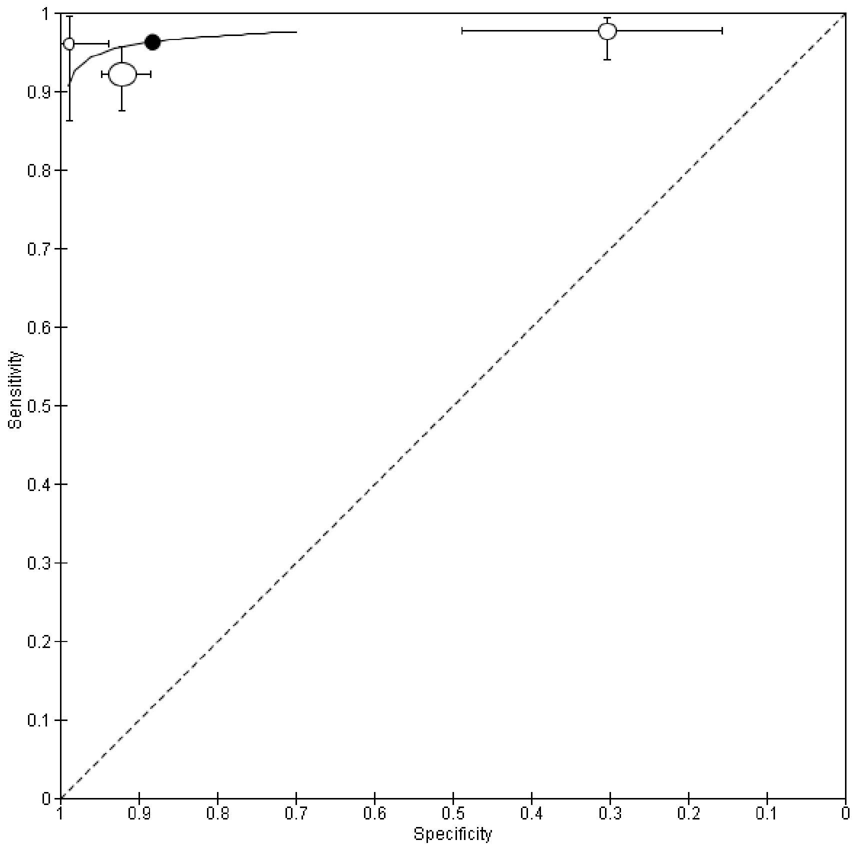

B) AWE-SEA Elisa vs quadruple KK

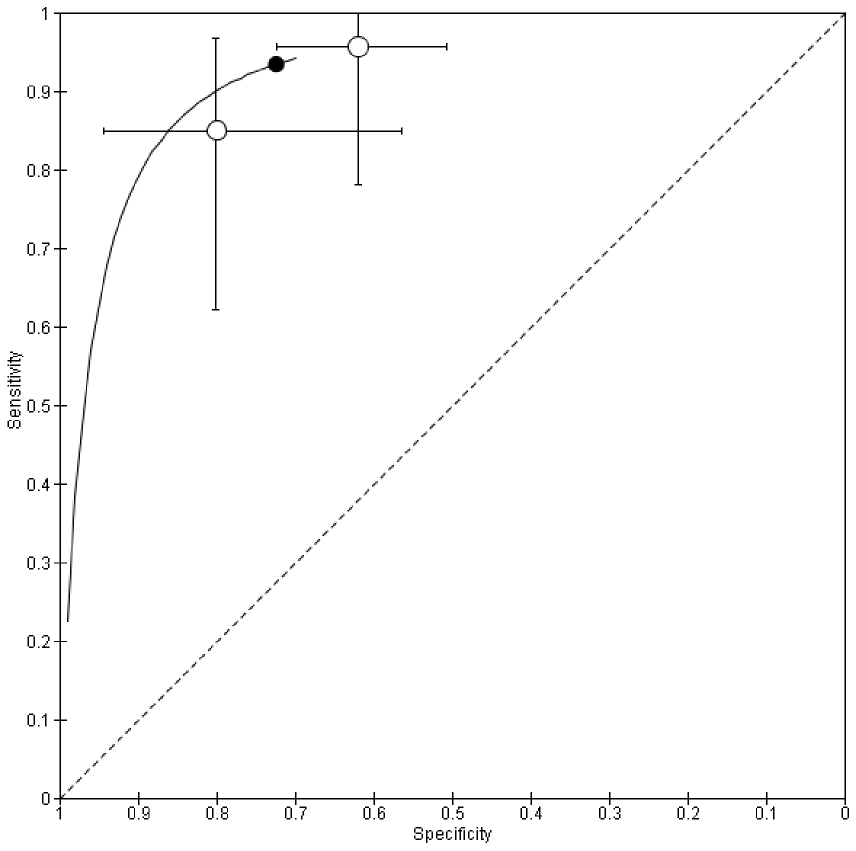

C) SWAP Elisa vs KK

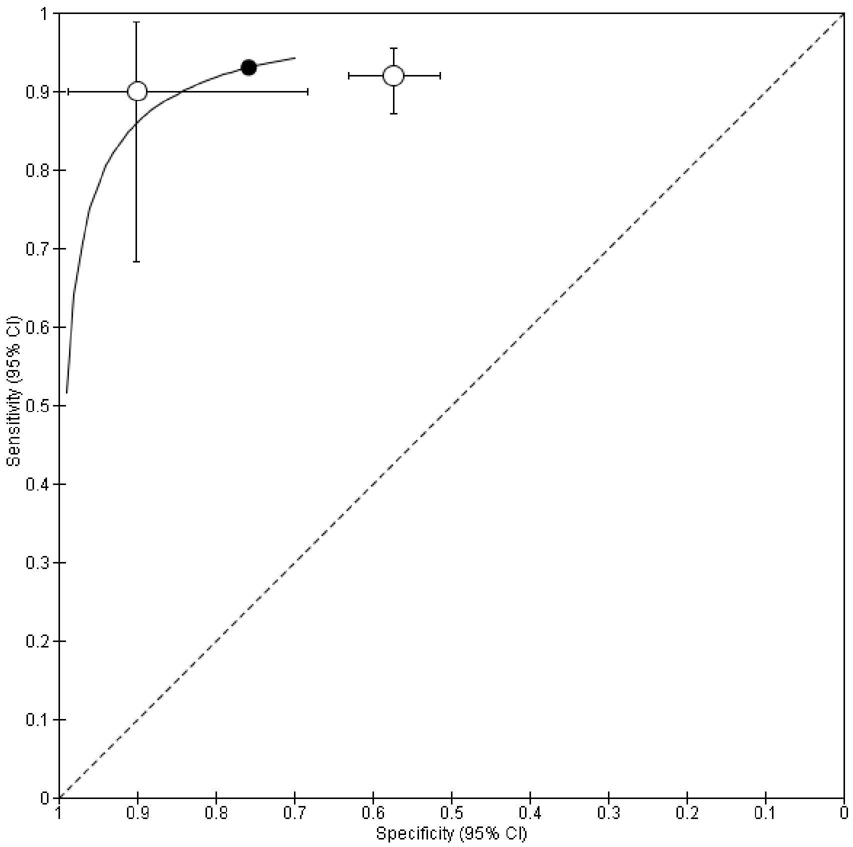

D) IgG SEA Elisa vs urine microscopy

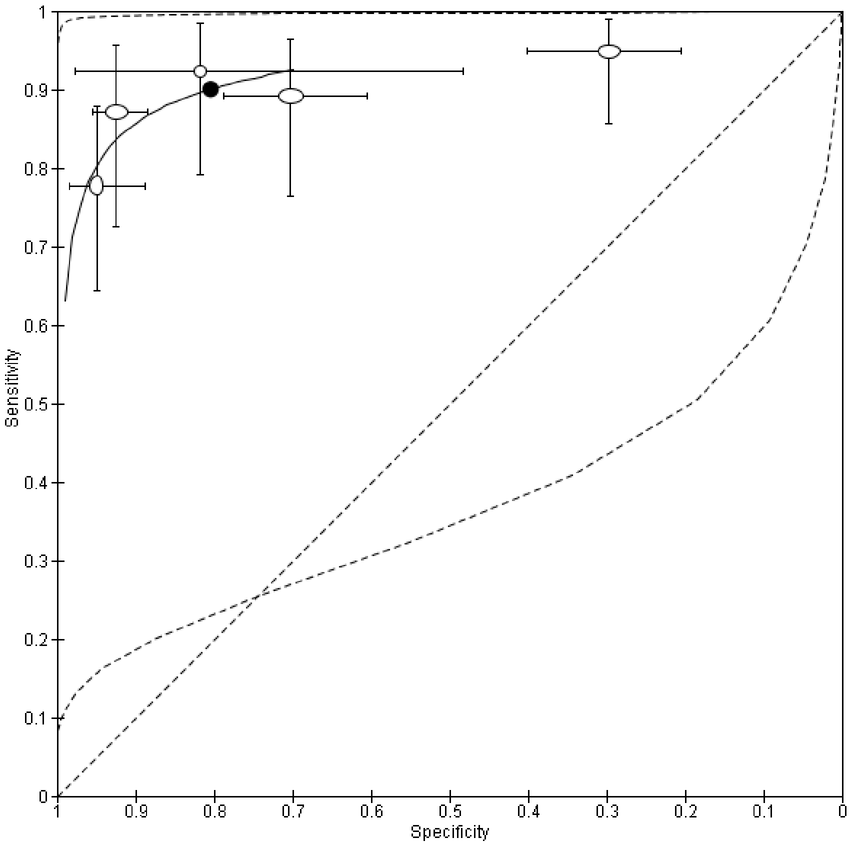

E) PCR vs KK

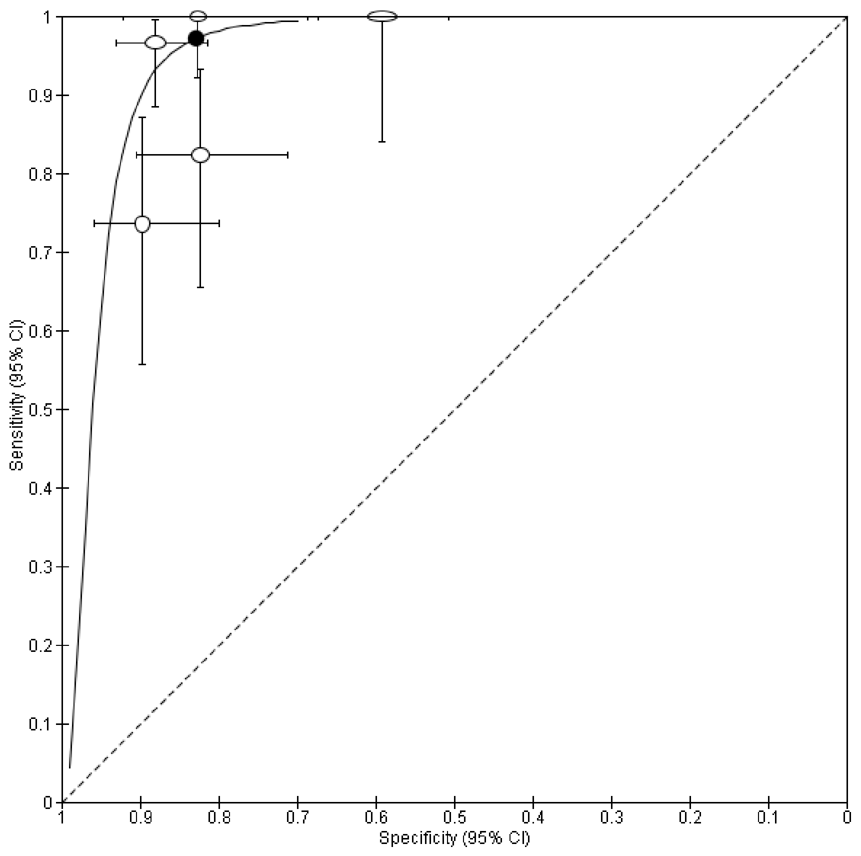

F) RT-PCR vs KK

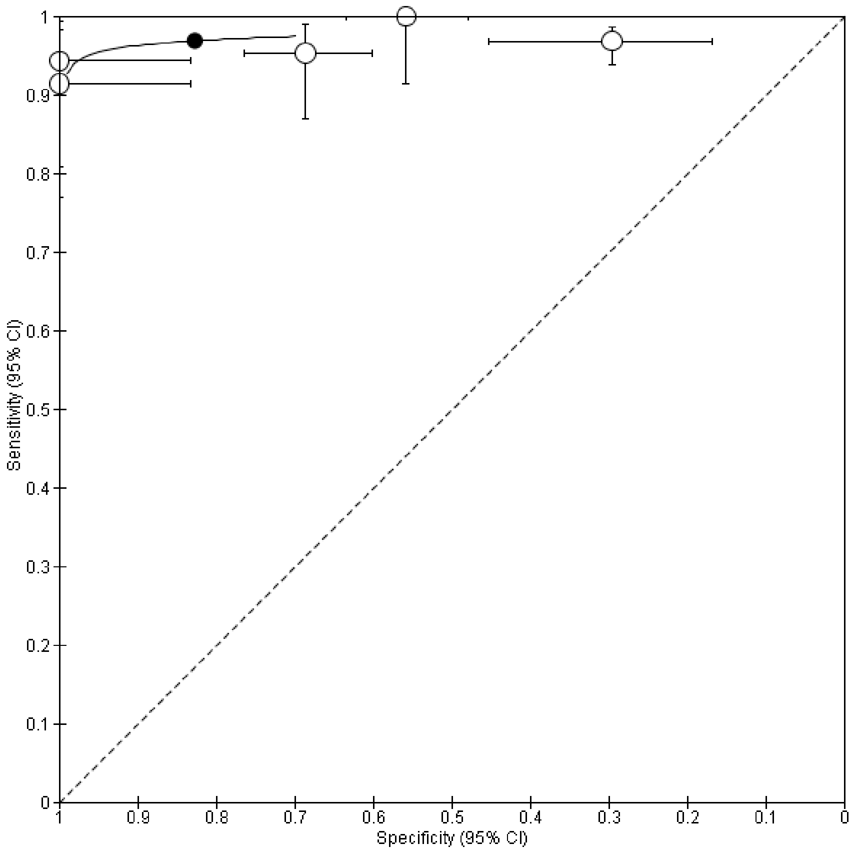

G) LAMP vs KK or urine microscopy

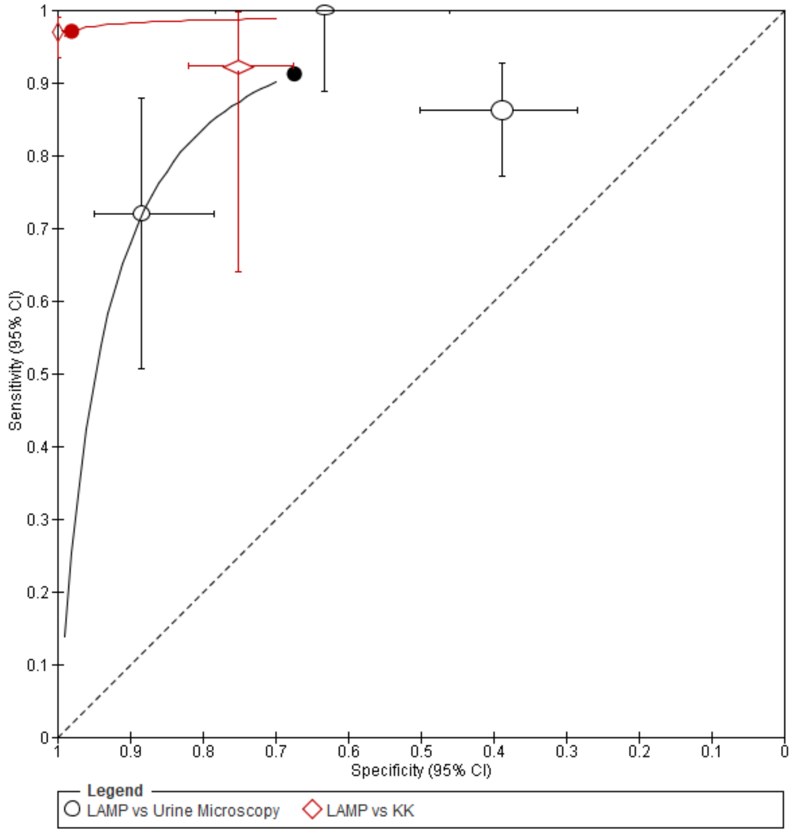

Abbreviation: AWE=adult worm extract, KK=Kato-Katz thick smear, LAMP=loop-mediated isothermal amplification, PCR=polymerase chain reaction, RT=real-time, SEA=soluble egg antigen, SWAP=soluble adult worm antigen preparation.

Legend: Each unfilled symbol represents the individual study estimates pair (Se, Sp), larger symbols reflect a higher sample size. The solid circles are the meta-analytic estimates across the studies included in the meta-analysis, the solid lines the summary HSROC curves and the dashed-curves the 95% prediction regions for a future study

4.11: Deek's funnel plots whenever data from more than four studies were available

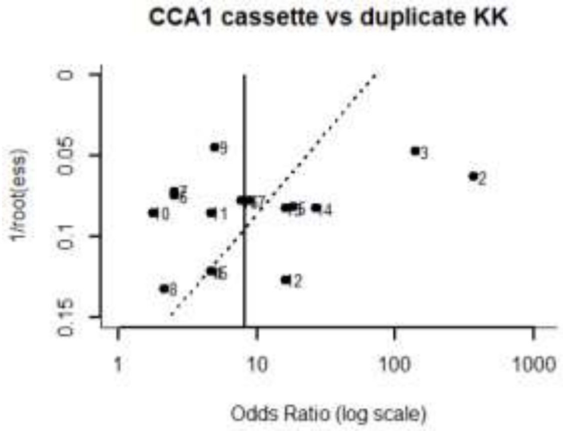

p-value= 0.172

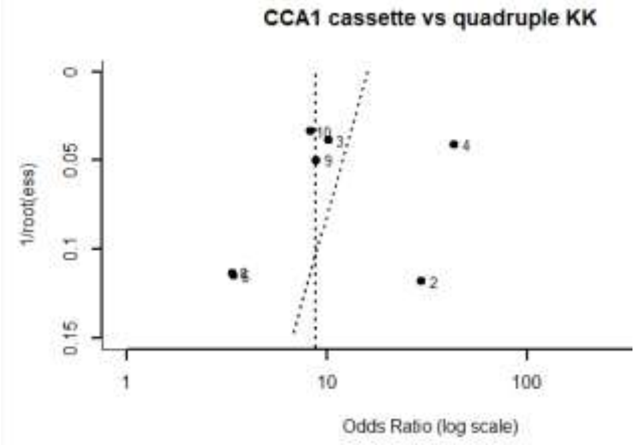

p-value=0.504

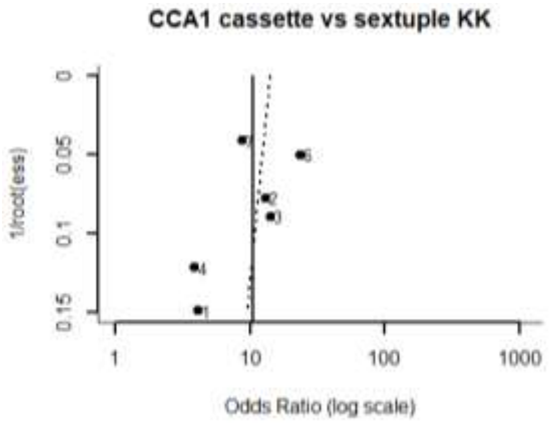

p-value=0.720

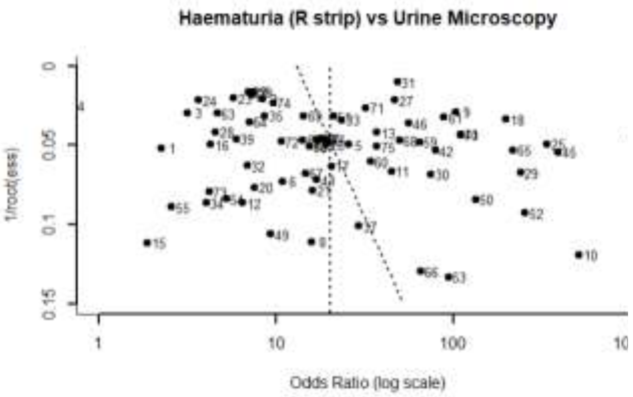

p-value=0.247

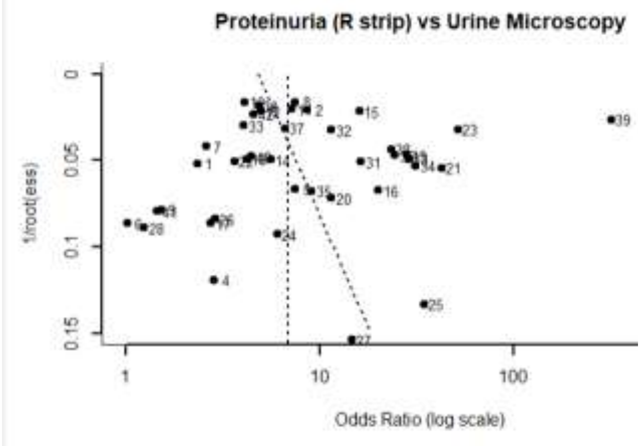

p-value=0.517

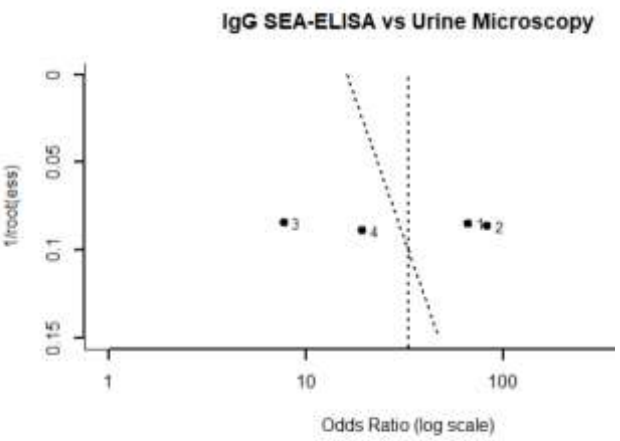

p-value=0.808

Abbreviations : CCA1= circulating cathodic antigen urine cassette assay v1, KK=Kato-Katz thick smear, SEA=soluble egg antigen

Legend: For the p-value, a regression test for funnel plot asymmetry has been used

4.12: Assessment of heterogeneity for Se, Sp and DOR whenever data from more than four studies were available

Test comparison	Number of test comparisons	Se		Sp		DOR						DOR					
		Heterogeneity test Se		Heterogeneity test Sp		Heterogeneity test DOR			DOR	DOR 95%CI		Funnel Plot Asymmetry		Rank Correlation Asymetry		Deek's plot Asymetry	
		I2 (%)	p(I2)	I2 (%)	p(I2)	I2 (%)	H2	p(Q)		LL	UL	Z	p-value	Kendall's Tau	p-value	t	P-value
**S. mansoni**																	
CCA1 vs duplicate KK	17	94.53	<0.0001	96.99	<0.0001	88.66	8.82	<0.0001	2.10	1.47	2.74	1.97	0.0494	0.03	0.0907	1.44	0.1716
CCA1 vs quadruple KK	10	84.35	<0.0001	95.95	<0.0001	83.71	6.14	<0.0001	2.18	1.58	2.78	-0.36	0.7175	0.16	0.6007	-0.70	0.5039
CCA1 vs sextuple KK	7	82.64	<0.0001	83.03	<0.0001	59.48	2.47	0.03	2.36	1.85	2.86	0.05	0.9632	-0.05	1	-0.38	0.7201
CCA1 vs KK (all)	36	96.14	<0.0001	95.69	<0.0001	89.15	9.22	<0.0001	1.98	1.57	2.39	1.92	0.0554	0.10	0.3759	-0.95	0.3491
CAA vs duplicate KK	3																
SmCTF-RDT vs quadruple KK	4	86.56	<0.0001	26.52	0.252648	70.49	3.39	0.0472	0.75	-0.73	2.23	1.78	0.075	0.33	0.75	-1.40	0.297
IgG Elisa vs triplicate KK	3																
AWE-SEA Elisa vs KK	2																
SWAP Elisa vs KK	2																
PCR vs KK	5	82.27	0.00015	90.65	<0.0001	60.25	2.52	0.0522	4.07	2.92	5.21	1.56	0.1195	0.60	0.2333	-0.98	0.4002
RT-PCR vs KK	5	19.14	0.292766	91.53	<0.0001	64.33	2.8	0.0354	4.16	2.76	5.56	2.93	0.0033	1.00	0.0167	2.40	0.0959
LAMP vs KK	2																
**S. haematobium**																	

CCA1 vs urine filtration	4	96.1	<0.0001	96.46	<0.0001	74.79	3.97	0.007	1.30	0.58	2.01	0.37	0.7092	0.67	0.3333	0.26	0.8191
CAA vs urine filtration	4	97.95	<0.0001	98.8	<0.0001	95.04	20.17	0.0015	2.61	0.48	4.73	3.00	0.0027	0.67	0.3333	0.32	0.7819
Proteinuria vs urine filtration	42	98.85	0	99.62	0	98.41	62.77	<0.0001	1.93	1.51	2.35	0.31	0.7546	-0.04	0.7466	0.65	0.5165
Haematuria vs urine filtration	75	97.65	0	99.45	0	98.29	58.57	<0.0001	3.01	2.69	3.33	4.85	<0.0001	0.21	0.0077	1.17	0.2474
Leukocyturia vs urine filtration	5	88.47	<0.0001	98.55	<0.0001	86.66	7.5	<0.0001	0.73	-0.10	1.57	-1.03	0.3009	-0.20	0.8167	-1.63	0.2019
IgG SEA Elisa vs urine filtration	5	54.29	<0.0001	97.73	<0.0001	64.02	2.78	0.0262	3.50	2.60	4.41	0.09	0.9301	-0.20	0.8167	0.27	0.8078
LAMP vs urine filtration	3																
