# Supplementary material for: Diagnostic tests for human Schistosoma mansoni and Schistosoma haematobium infection: a systematic review and meta-analysis
Source: Lancet Microbe. 2024 Apr;5(4):e366–78. doi: 10.1016/S2666-5247(23)00377-4 (PMC10990967; doi:10.1016/S2666-5247(23)00377-4)
Supplement: Supplementary appendix 2 [file mmc2.pdf]

THE LANCET
Microbe

Supplementary appendix 2

This appendix formed part of the original submission and has been peer reviewed. We post it as supplied by the authors.

Supplement to: Vaillant MT, Philippy F, Neven A, et al. Diagnostic tests for human *Schistosoma mansoni* and *Schistosoma haematobium* infection: a systematic review and meta-analysis. *Lancet Microbe* 2024. https://doi.org/10.1016/S2666-5247(23)00377-4

Author et al	Study design	Age cat.	location	Study type	Inclusion	comments
Abdel-Wahab 1992	cross-sectional	SAC	Egypt	field-study	Included	Praziquantel trt: half of children have received trt
Adbel-Wahab 200	cross-sectional	5-25y	Egypt		Included	
Adriko 2014	cross-sectional	SAC	Uganda	field-study	Included	Praziquantel trt: Annual mass trt with praziquantel had reduced endemicity from high to medium level
Al-Shehri 2018	cross-sectional	SAC	Uganda	field-study	Included	
Al-Sherbiny 1999	cross-sectional	not available	Nigeria	field-study	Included	
Anosike 2001	cross-sectional	not available	Nigeria	field-study	Included	
Anyan 2020	cross-sectional	not available	Brazil	field-study	Included	
Aryeetey 2000	cross-sectional	not available	Ghana	field-study	Included	
Assaré 2018	cross-sectional	SAC	Ivory Coast	field-study	Included	
Ayele 2008	cross-sectional	SAC	Ethiopia	field-study	Included	
Barakat 1983	cross-sectional	SAC	Egypt	field-study	Included	
Bassiouny 2014	cross-sectional	SAC	Yemen		Included	
Bezerra 2021	cross-sectional	not available	Brazil	field-study	Included	
Birrie 1995 HPA (high prevalence area)	cross-sectional	not available	Ethiopia	field-study	Included	
Birrie 1995 LPA (low prevalence area)	cross-sectional	not available	Ethiopia	field-study	Included	
Birrie 1995 MPA	cross-sectional	not available	Ethiopia	field-study	Included	
Bocanegra 2015	cross-sectional	SAC	Angola		Included	Drug before the study: 65 children (4.5%) reported receiving treatment with praziquantel in the two previous months and 22 (1.5%) with mebendazole or albendazole
Bogoch 2021	cross-sectional	all	Ghana	field-study	Included	Praziquantel before the study: Empiric therapy with albendazole and ivermectin 4 months prior to the study.
Bosompem 1996	cross-sectional	all	Ghana	field-study	Included	
Bosompem 2004	cross-sectional	SAC	Ghana	field-study	Included	
Bouilhac 1981	not available	not available	unknown		Included	sample analysis
Chernet 2017	cross-sectional	migrants	Eritrea	field-study	Included	
Colley 2013 (Cameroon)	cross-sectional	SAC	Cameroon	field-study	Included	
Colley 2013 (Ivory Coast)	cross-sectional	SAC	Ivory Coast	field-study	Included	
Cooppan 1987	cross-sectional	SAC	South Africa	field-study	Included	
Coulibaly 2013	cross-sectional	<6y	Ivory Coast	field-study	Included	No trt within the past 6 months
Dawson 2013	cross-sectional	PSAC	Uganda	field-study	Included	No previous trt
De Clercq 1995	cross-sectional	not available	Mali	field-study	Included	No previous trt
De Oliveira 1995	cross-sectional	not available	Brazil		Included	
Elbascheir 2020	longitudinal survey	SAC	Central Soudan	field-study	Included	
El Morshedy 1996	cross-sectional	men from military camp	Egypt	military camp	Included	
El Sayed 1995	cross-sectional	permanent settlers	Egypt	field-study	Included	
Eltiro 1992	cross-sectional	SAC	Ethiopia	field-study	Included	
Eltoum 1992	cross-sectional	3-39y	Sudan	field-study	Included	
Espirito-Santo 2015	cross-sectional	>5y	Brazil	field-study	Included	No trt within 1 year
Fatiregun 2005	cross-sectional	SAC	Nigeria	field-study	Included	
Fereira 2017	cross-sectional	7-76y	Brazil	field-study	Included	
French 2007	cross-sectional	SAC	Tanzania	field-study	Included	
Fuss 2018	cross-sectional	SAC	Tanzania	field-study	Included	
Gabr 2000	cross-sectional	not available	Egypt	field-study	Included	
Gandasegui 2015	sample analysis	Sub-saharan immigrants	Spain	health diagnostics in hospita	Included	
Gandasegui 2018	cross-sectional	SAC	Angola	field-study	Included	
Glintz 2010	cross-sectional	SAC	Ivory Coast	field-study	Included	
Gunderson 1996	cross-sectional	women of childbearing ag	Malawi	outpatient department of h	Included	
Hammad 1997	cross-sectional	not available	Egypt	field-study	Included	
Hammam 2000a	cross-sectional	not available	Egypt	field-study	Included	
Hammam 2000b	cross-sectional	not available	Egypt	field-study	Included	
Kassim 1989	cross-sectional	SAC	Nigeria	field-study	Included	
Kiliku 1991	cross-sectional	not available	Kenya	field-study	Included	
King 1988a	cross-sectional	SAC	Kenya	field-study	Included	
King 1988b	cross-sectional	not available	Kenya	field-study	Included	
Kitange 1993	cohort study	SAC	Tanzania	field-study	Included	Praziquantal trt : 1 year prior to study

Author et al	Study design	Age cat.	location	Study type	Inclusion	comments
Knopp 2015	cross-sectional	SAC	Tanzania	field-study	Included	Praziquantal trt within study year
Knopp 2018	cross-sectional	adults+children	Zanzibar		Included	
Kosinki 2011	cross-sectional	SAC	Ghana	field-study	Included	
Lamberton 2014	cross-sectional	SAC	Uganda	field-study	Included	
Legesse 2008	cross-sectional	SAC	Ethiopia		Included	
Lengeler 1993	cross-sectional	SAC	Tanzania	field-study	Included	
Lindholz 2018	cross-sectional	1-17y	Brazil	field-study	Included	
Lodh 2013	cross-sectional	18-50y	Zambia	field-study	Included	
Mafe 1997	cross-sectional	>5y	Nigeria		Included	No previous trt
Mafe 2000	cross-sectional	SAC	Nigeria	field-study	Included	
Magalhaes 2020	cross-sectional	2-88y	Brazil	field-study	Included	
Magnussen 2001	cohort study	SAC	Tanzania	field-study	Included	
Mazigo 2018	cross-sectional	5-55y	Tanzania	field-study	Included	No trt within the past 6 months
Midzi 2009	national survey	2-19y	Zimbabwe	field-study	Included	is it not cross-sectional?
Morenikeji 2014	cross-sectional	children of all ages	Nigeria	field-study	Included	
Mott 1985 Ghana	cohort study	children and adults	Ghana	field-study	Included	
Mott 1985 Zambia	cohort study	children and adults	Zambia	field-study	Included	
Mtasiwa 1996	cross-sectional	SAC	Tanzania	field-study	Included	
Murare 1987	cohort study	SAC	Zimbabwe	field-study	Included	
Mwangi 2018	cross-sectional	SAC	Kenya	field-study	Included	
N'Goran 1989	cross-sectional	not available	ivory Coast	field-study	Included	
Nausch 2014	cross-sectional	1-12y	Zimbabwe	field-study	Included	
Navaratnam 2012	cross-sectional	PSAC	Uganda	field-study	Included	
Ndamukong 2001	cross-sectional	SAC	Cameroon	field-study	Included	
Ndhlovu 1996	nested case-control	not available	Zimbabwe	field-study	Included	
Nduka 1995	cross-sectional	SAC	Nigeria	field-study	Included	
Ndyomugyenyi 2001	cross-sectional	SAC	Tanzania	field-study	Included	
Ng'andu 1988	cross-sectional	SAC	Zambia	field-study	Included	
Ngasala 2020 (Mta Dam area)	cross-sectional	SAC	Tanzania	field-study	Included	
Ngasala 2020 (Uwandani)	cross-sectional	SAC	Tanzania	field-study	Included	
Nwaorgu 1992	cross-sectional	all	Nigeria	field-study	Included	
Ofori 1986	cross-sectional	SAC	Ghana	field-study	Included	
Okeke 2014 (HPA)	cross-sectional	SAC	Nigeria		Included	
Okeke 2014 (LPA)	cross-sectional	SAC	Nigeria		Included	
Onayada 1996	cross-sectional	SAC	Nigeria	field-study	Included	
Poggensee 2000 HPA	cross-sectional	childbearing women	Tanzania		Included	
Poggensee 2000 LPA	cross-sectional	childbearing women	Tanzania		Included	
Polman 1995	cross-sectional	0-77y	Senegal	field-study	Included	
Pugh 1980	cross-sectional	males between 5-25y	Nigeria	field-study	Included	
Rasendramino 1998	cross-sectional	>5y	Madagascar	field-study	Included	
Robinson 2009	quasi-random two-stage, nested case-control	children SAC	South Soudan		Included	part of a large scale survey
Rollinson 2005	cross-sectional	SAC	Tanzania	field-study	Included	
Sarda 1986	cross-sectional	SAC	Tanzania	field-study	Included	
Savioli 1990	cross-sectional	SAC	Tanzania	field-study	Included	
Sellin 1982	cross-sectional	not available	Burkina Faso	field-study	Included	
Shane 2011	cross-sectional	children	Kenya	field-study	Included	No mass drug administration
Shaw 1998	cohort study	not available	Senegal	field-study	Included	
Sheele 2013	cross-sectional	SAC	Kenya	field-study	Included	
Song 2018	cross-sectional	not available	Sudan	field-study	Included	
Sousa 2020	cross-sectional	not available	Brazil	field-study	Included	
Standley 2010	cross-sectional	SAC	Tanzania+Kenya		Included	
Stephenson 1984	cross-sectional	SAC	Kenya	field-study	Included	
Stothard 2009a	cross-sectional	SAC	Tanzania	field-study	Included	
Stothard 2009b	cross-sectional	SAC	Tanzania	field-study	Included	
Tanner 1983 (Liberia)	cross-sectional	SAC	Liberia	field-study	Included	
Tanner 1983 (Tanzania)	cross-sectional	SAC	Tanzania	field-study	Included	

Author et al	Study design	Age cat.	location	Study type	Inclusion	comments
Tchuem Tchuente 2012	cross-sectional	SAC	Cameroon	field-study	Included	
Traore 1998	cross-sectional	>2y	Mali	field-study	Included	
Uga 1989	not available	not available	Kenya		Included	sample analysis
Ugbomoiko 2009a	cross-sectional	not available	Nigeria	field-study	Included	
Ugbomoiko 2009b	cross-sectional	SAC	Nigeria		Included	
Van Lieshout 1995	cross-sectional	not available	Surinam	field-study	Included	
Verle 1994	cross-sectional	not available	Senegal	field-study	Included	
Vonghachack 2017	cross-sectional	>6y	Laos/Cambodia	field-study	Included	
Wilkins 1979	cross-sectional	not available	Gambia	field-study	Included	
Xu 2014	cross-sectional	5-78y	China	field-study	Included	
Xu 2015	cross-sectional	not available	China	field-study	Included	
Zhang 2020	not available	SAC	Zambia	field-study	Included	Sample analysis
Zumstein 1983	cross-sectional	SAC	Tanzania	field-study	Included	
Deribew 2021	cross-sectional	children 5-15y	Ethiopia	field-study	Excluded	
Mahmoud 2021	cross-sectional	schoolchildren	Egypt	field-study	Excluded	
Mohammed 2022	longitudinal survey	adults+children	Ethiopia	field-study	Excluded	
Gandasegui 2018	cross-sectional	adults+children	Brazil	field-study	Excluded	
Bayoumi 2016	not available	not available	Egypt	outpatient clinic	Excluded	
Grenfell 2013	cross-sectional	adults	Brazil	field-study	Excluded	
Senra 2018	cross-sectional	adults+children	Brazil		Excluded	
Schunk 2015	cross-sectional	adults+children	Ethiopia	field-study	Excluded	
Oliveira 2010	cross-sectional	adults+children	Brazil	field-study	Excluded	
Pontes 2003	cross-sectional	adults+children	Brazil		Excluded	
Coulibaly 2022	cross-sectional	schoolchildren	Ivory Coast		Excluded	

ID	author et al	reference number	the study was publi	year the study was done	study location	diagnostic assay investigated	reference diagnostic assay	parasite specie considered	any other relevant information
1	Sousa 2020	62	2020		Brazil	CCA1 cassette	single KK		
2	Adriko 2014	49	2014		Uganda	CCA1 cassette	duplicate KK	S. mansoni	
3	Al-Shehri 2018	63	2018		Uganda	CCA1 cassette	duplicate KK	S. mansoni	
4	Elbasheir 2020	33	2020		Central Soudan	CCA1 cassette	duplicate KK	S. mansoni	
5	Ferreira 2017	64	2017		Brazil	CCA1 cassette	duplicate KK	S. mansoni	
6	Fuss 2018	65	2018		Tanzania	CCA1 cassette	duplicate KK	S. mansoni	
7	Legesse 2008	66	2008		Ethiopia	CCA1 cassette	duplicate KK	S. mansoni	
8	Lindholz 2018	53	2018		Brazil	CCA1 cassette	duplicate KK	S. mansoni	
9	Lodh 2013	51	2013		Zambia	CCA1 cassette	duplicate KK	S. mansoni	
10	Navaratnam 2012	67	2012		Uganda	CCA1 cassette	duplicate KK	S. mansoni	
11	Polman 1995	68	1995		Senegal	CCA1 cassette	duplicate KK	S. mansoni	
12	Polman 1995	68	1995		Senegal	CCA1 cassette	duplicate KK	S. mansoni	
13	Sousa 2020	62	2020		Brazil	CCA1 cassette	duplicate KK	S. mansoni	
14	Standley 2010	69	2010		Tanzania+Keny	CCA1 cassette	duplicate KK	S. mansoni	
15	Standley 2010	69	2010		Tanzania+Keny	CCA1 cassette	duplicate KK	S. mansoni	
16	Tchuem Tchuente 2012	70	2012		Cameroon	CCA1 cassette	duplicate KK	S. mansoni	
17	Van Lieshout 1995	71	1995		Surinam	CCA1 cassette	duplicate KK	S. mansoni	
18	Van Lieshout 1995	71	1995		Surinam	CCA1 cassette	duplicate KK	S. mansoni	
19	Adriko 2014	49	2014		Uganda	CCA1 cassette	quadruple KK	S. mansoni	
20	Chernet 2017	43	2017		Eritrea	CCA1 cassette	quadruple KK	S. mansoni	
21	Colley 2013 cameroon	72	2013		Cameroon	CCA1 cassette	quadruple KK	S. mansoni	
22	Colley 2013 Côte d'ivoire	72	2013		Ivory Coast	CCA1 cassette	quadruple KK	S. mansoni	
23	Coulibaly 2013	56	2013		Ivory Coast	CCA1 cassette	quadruple KK	S. mansoni	
24	Dawson 2013	73	2013		Uganda	CCA1 cassette	quadruple KK	S. mansoni	
25	Dawson 2013	73	2013		Uganda	CCA1 cassette	quadruple KK	S. mansoni	
26	Ferreira 2017	64	2017		Brazil	CCA1 cassette	quadruple KK	S. mansoni	
27	Mazigo 2018	74	2018		Tanzania	CCA1 cassette	quadruple KK	S. mansoni	
28	Shane 2011	75	2011		Kenya	CCA1 cassette	quadruple KK	S. mansoni	
29	Adriko 2014	49	2014		Uganda	CCA1 cassette	sextuple KK	S. mansoni	
30	Assaré, 2018	76	2018		Ivory Coast	CCA1 cassette	sextuple KK	S. mansoni	
31	Bezerra 2020	77	2020		Brazil	CCA1 cassette	sextuple KK	S. mansoni	
32	Fereira 2017	64	2017		Brazil	CCA1 cassette	sextuple KK	S. mansoni	
33	Lamberton 2014	78	2014		Uganda	CCA1 cassette	sextuple KK	S. mansoni	
34	Shane 2011	75	2011		Kenya	CCA1 cassette	sextuple KK	S. mansoni	
35	Tchuem Tchuente 2012	70	2012		Cameroon	CCA1 cassette	sextuple KK	S. mansoni	
36	Sousa 2020	62	2020		Brazil	CCA1 cassette	16KK	S. mansoni	
37	Adriko 2014	49	2014		Uganda	CCA2 cassette	double KK	S. mansoni	
38	Adriko 2014	49	2014		Uganda	CCA2 cassette	quadruple KK	S. mansoni	
39	Polman 1995	68	1995		Senegal	CAA	duplicate KK	S. mansoni	
40	Van Lieshout 1995	71	1995		Surinam	CAA	duplicate KK	S. mansoni	
41	Van Lieshout 1995	71	1995		Surinam	CAA	duplicate KK	S. mansoni	
42	Glinz 2010	50	2010		Ivory Coast	FLOTAC (fresh)	triplicate KK	S. mansoni	
43	Glinz 2010	50	2010		Ivory Coast	FLOTAC (10 days)	triplicate KK	S. mansoni	
44	Glinz 2010	50	2010		Ivory Coast	FLOTAC (30 days)	triplicate KK	S. mansoni	
45	Coulibaly 2013	56	2013		Ivory Coast	SmCTF-RDT	quadruple KK	S. mansoni	
46	Dawson 2013	73	2013		Uganda	SmCTF-RDT	quadruple KK	S. mansoni	
47	Dawson 2013	73	2013		Uganda	SmCTF-RDT	quadruple KK	S. mansoni	
48	Nausch 2014	140	2014		Zimbabwe	SmCTF-RDT	quadruple KK	S. mansoni	
49	Coulibaly 2013	56	2013		Ivory Coast	SmCTF-RDT	Urine Microscopy	S. haematobium	

ID	author et al	reference number	the study was publi	year the study was done	study location	diagnostic assay investigated	reference diagnostic assay	parasite specie considered	any other relevant information
50	Lodh 2013	51	2013		Zambia	Sm DNA PCR	Sm DNA duplicate KK	S. mansoni	
51	Shane 2011	75	2011		Kenya	SWAP ELISA	Sextuple KK	S. mansoni	
52	Grenfell 2013	143	2013		Brazil	SWAP ELISA	18 KK	S. mansoni	
53	De Oliveira 2005	52	2005		Brazil	IgM ELISA	triplicate KK	S. mansoni	
54	Barakat 1983	141	1983		Egypt	IgG ELISA	triplicate KK	S. mansoni	
55	De Oliveira 2005	52	2005		Brazil	IgG ELISA	triplicate KK	S. mansoni	
56	Eltiro 1992	142	1992		Ethiopia	IgG ELISA	triplicate KK	S. mansoni	
57	Chernet 2017	43	2017		Eritrea	AWE-SEA ELISA	quadruple KK	S. mansoni	
58	Grenfell 2013	143	2013		Brazil	AWE-SEA ELISA	18 KK	S. mansoni	
59	Al-Shehri 2018	63	2018		Uganda	IgG SEA-ELISA	CCA1		
60	Bouilhac 1981	42	1981			IgG SEA-ELISA	Urine Microscopy	S. haematobium	
61	Mahmoud 2021	60	2021			IgG SEA-ELISA	Urine Microscopy	S. haematobium	
62	Song 2018	144	2018		Sudan	IgG SEA-ELISA	Urine Microscopy	S. haematobium	
63	Stothard 2009a	82	2009		Tanzania	IgG SEA-ELISA	Urine Microscopy	S. haematobium	
64	Uga 1989	145	1989		Kenya	IgG SEA-ELISA	Urine Microscopy	S. haematobium	
65	Mahmoud 2021	60	2021			IMB-based ELISA	Urine Microscopy	S. haematobium	
66	Sheele 2013	58	2013		Kenya	Anti IGg RDT-Sh	Urine Microscopy	S. haematobium	
67	Abdel-Wahab 1992	45	1992		Egypt	Haematuria (R strip)	Urine Microscopy	S. haematobium	
68	Abdel-Wahab 2000	85	2000		Egypt	Haematuria (R strip)	Urine Microscopy	S. haematobium	
69	Anosike 2001	116	2001		Nigeria	Haematuria (R strip)	Urine Microscopy	S. haematobium	
70	Aryeetey 2000	86	2000		Ghana	Haematuria (R strip)	Urine Microscopy	S. haematobium	
71	Bassiouny 2014	117	2014		Yemen	Haematuria (R strip)	Urine Microscopy	S. haematobium	
72	Birrie 1995 (HPA)	118	1995		Ethiopia	Haematuria (R strip)	Urine Microscopy	S. haematobium	
73	Birrie 1995 (LPA)	118	1995		Ethiopia	Haematuria (R strip)	Urine Microscopy	S. haematobium	
74	Birrie 1995 (MPA)	118	1995		Ethiopia	Haematuria (R strip)	Urine Microscopy	S. haematobium	
75	Bocanegra 2015	46	2015		Angola	Haematuria (R strip)	Urine Microscopy	S. haematobium	
76	Bogoch 2012	47	2012		Ghana	Haematuria (R strip)	Urine Microscopy	S. haematobium	
77	Bosompem 1996	87	1996		Ghana	Haematuria (R strip)	Urine Microscopy	S. haematobium	
78	Bosompem 2004	88	2004		Ghana	Haematuria (R strip)	Urine Microscopy	S. haematobium	
79	Cooppan 1987	89	1987		South Africa	Haematuria (R strip)	Urine Microscopy	S. haematobium	
80	Deribew 2022	139	2022			Haematuria (R strip)	Urine Microscopy	S. haematobium	
81	El-Sayed 1995	119	1995		Egypt	Haematuria (R strip)	Urine Microscopy	S. haematobium	
82	Eltoum 1992	120	1992		Sudan	Haematuria (R strip)	Urine Microscopy	S. haematobium	
83	Fatiregun 2005	121	2005		Nigeria	Haematuria (R strip)	Urine Microscopy	S. haematobium	
84	French 2007	122	2007		Tanzania	Haematuria (R strip)	Urine Microscopy	S. haematobium	
85	Gabr 2000	90	2000		Egypt	Haematuria (R strip)	Urine Microscopy	S. haematobium	
86	Gandasegui 2018	152	2018		Angola	Haematuria (R strip)	Urine Microscopy	S. haematobium	
87	Gundersen 1996	91	1996		Malawi	Haematuria (R strip)	Urine Microscopy	S. haematobium	
88	Hammad 1997	92	1997		Egypt	Haematuria (R strip)	Urine Microscopy	S. haematobium	
89	Hammam 2000a	93	2000		Egypt	Haematuria (R strip)	Urine Microscopy	S. haematobium	
90	Hammam 2000b	94	2000		Egypt	Haematuria (R strip)	Urine Microscopy	S. haematobium	
91	Kassim 1989	95	1989		Nigeria	Haematuria (R strip)	Urine Microscopy	S. haematobium	
92	Kiliku 1991	96	1991		Kenya	Haematuria (R strip)	Urine Microscopy	S. haematobium	
93	King 1988a	97	1988		Kenya	Haematuria (R strip)	Urine Microscopy	S. haematobium	
94	King 1988b	123	1988		Kenya	Haematuria (R strip)	Urine Microscopy	S. haematobium	
95	Kitange 1993	37	1993		Tanzania	Haematuria (R strip)	Urine Microscopy	S. haematobium	
96	Knopp 2015	48	2015		Tanzania	Haematuria (R strip)	Urine Microscopy	S. haematobium	
97	Knopp 2018	124	2018		Zanzibar	Haematuria (R strip)	Urine Microscopy	S. haematobium	
98	Kosinski 2011	125	2011		Ghana	Haematuria (R strip)	Urine Microscopy	S. haematobium	

ID	author et al	reference number	the study was publi	year the study was done	study location	diagnostic assay investigated	reference diagnostic assay	parasite specie considered	any other relevant information
99	Lengeler 1993	126	1993		Tanzania	Haematuria (R strip)	Urine Microscopy	S. haematobium	
100	Mahmoud 2021	60	2021			Haematuria (R strip)	Urine Microscopy	S. haematobium	
101	Mafe 1997	127	1997		Nigeria	Haematuria (R strip)	Urine Microscopy	S. haematobium	
102	Mafe 2000	128	2000		Nigeria	Haematuria (R strip)	Urine Microscopy	S. haematobium	
103	Magnussen 2001	38	2001		Tanzania	Haematuria (R strip)	Urine Microscopy	S. haematobium	
104	Mohammed 2022	34	2022			Haematuria (R strip)	Urine Microscopy	S. haematobium	
105	Morenikeji 2014	129	2014		Nigeria	Haematuria (R strip)	Urine Microscopy	S. haematobium	
106	Mott 1985 Ghana	39	1985		Ghana	Haematuria (R strip)	Urine Microscopy	S. haematobium	
107	Mott 1985 Zambia	39	1985		Zambia	Haematuria (R strip)	Urine Microscopy	S. haematobium	
108	Mtasiwa 1996	130	1996		Tanzania	Haematuria (R strip)	Urine Microscopy	S. haematobium	
109	Murare 1987	40	1987		Zimbabwe	Haematuria (R strip)	Urine Microscopy	S. haematobium	
110	N'Goran 1989	131	1989		ivory Coast	Haematuria (R strip)	Urine Microscopy	S. haematobium	
111	Ndamukong 2001	98	2001		Cameroon	Haematuria (R strip)	Urine Microscopy	S. haematobium	
112	Nduka 1995	132	1995		Nigeria	Haematuria (R strip)	Urine Microscopy	S. haematobium	
113	Ndyomugyenyi 2001	133	2001		Tanzania	Haematuria (R strip)	Urine Microscopy	S. haematobium	
114	Ng'andu 1988	99	1988		Zambia	Haematuria (R strip)	Urine Microscopy	S. haematobium	
115	Ngasala 2020 Mta Dam area	134	2020		Tanzania	Haematuria (R strip)	Urine Microscopy	S. haematobium	
116	Ngasala 2020 Uwandani Shehia	134	2020		Tanzania	Haematuria (R strip)	Urine Microscopy	S. haematobium	
117	Nwaorgu 1992	100	1992		Nigeria	Haematuria (R strip)	Urine Microscopy	S. haematobium	
118	Ofori 1986	101	1986		Ghana	Haematuria (R strip)	Urine Microscopy	S. haematobium	
119	Okeke 2014 (LPA)	102	2014		Nigeria	Haematuria (R strip)	Urine Microscopy	S. haematobium	
120	Okeke 2014 (MPA)	102	2014		Nigeria	Haematuria (R strip)	Urine Microscopy	S. haematobium	
121	Poggensee 2000 (HPA)	104	2000		Tanzania	Haematuria (R strip)	Urine Microscopy	S. haematobium	
122	Poggensee 2000 (LPA)	104	2000		Tanzania	Haematuria (R strip)	Urine Microscopy	S. haematobium	
123	Pugh 1980	105	1980		Nigeria	Haematuria (R strip)	Urine Microscopy	S. haematobium	
124	Rasendramino 1998	106	1998		Madagascar	Haematuria (R strip)	Urine Microscopy	S. haematobium	
125	Robinson 2009	36	2009		South Soudan	Haematuria (R strip)	Urine Microscopy	S. haematobium	
126	Rollinson 2005	135	2005		Tanzania	Haematuria (R strip)	Urine Microscopy	S. haematobium	
127	Sarda 1986	107	1986		Tanzania	Haematuria (R strip)	Urine Microscopy	S. haematobium	
128	Savioli 1990	136	1990		Zanzibar	Haematuria (R strip)	Urine Microscopy	S. haematobium	
129	Sellin 1982	108	1982		Burkina Faso	Haematuria (R strip)	Urine Microscopy	S. haematobium	
130	Shaw 1998	41	1998		Senegal	Haematuria (R strip)	Urine Microscopy	S. haematobium	
131	Stephenson 1984	109	2010		Kenya	Haematuria (R strip)	Urine Microscopy	S. haematobium	
132	Stothard 2009b	137	2009		Tanzania	Haematuria (R strip)	Urine Microscopy	S. haematobium	
133	Tanner 1983 (Liberia)	110	1983		Liberia	Haematuria (R strip)	Urine Microscopy	S. haematobium	
134	Tanner 1983 (Tanzania)	110	1983		Tanzania	Haematuria (R strip)	Urine Microscopy	S. haematobium	
135	Traore 1998	111	1998		Mali	Haematuria (R strip)	Urine Microscopy	S. haematobium	
136	Ugbomoiko 2009a	112	2009		Nigeria	Haematuria (R strip)	Urine Microscopy	S. haematobium	
137	Ugbomoiko 2009a	112	2009		Nigeria	Haematuria (R strip)	Urine Microscopy	S. haematobium	
138	Ugbomoiko 2009b	113	2009		Nigeria	Haematuria (R strip)	Urine Microscopy	S. haematobium	
139	Verle, 1994	114	1994		Senegal	Haematuria (R strip)	Urine Microscopy	S. haematobium	
140	Wilkins 1979	115	1979		Gambia	Haematuria (R strip)	Urine Microscopy	S. haematobium	
141	Zumstein 1983	138	1983		Tanzania	Haematuria (R strip)	Urine Microscopy	S. haematobium	
142	Abdel-Wahab 1992	45	1992		Egypt	Proteinuria (R strip)	Urine Microscopy	S. haematobium	
143	Abdel-Wahab 2000	85	2000		Egypt	Proteinuria (R strip)	Urine Microscopy	S. haematobium	
144	Aryeetey 2000	86	2000		Ghana	Proteinuria (R strip)	Urine Microscopy	S. haematobium	
145	Bogoch 2012	47	2012		Ghana	Proteinuria (R strip)	Urine Microscopy	S. haematobium	
146	Bosompem 1996	87	1996		Ghana	Proteinuria (R strip)	Urine Microscopy	S. haematobium	
147	Bosompem 2004	88	2004		Ghana	Proteinuria (R strip)	Urine Microscopy	S. haematobium	

ID	author et al	reference number	the study was publi	year the study was done	study location	diagnostic assay investigated	reference diagnostic assay	parasite specie considered	any other relevant information
148	Cooppan 1987	89	1987		South Africa	Proteinuria (R strip)	Urine Microscopy	S. haematobium	
149	Gabr 2000	90	2000		Egypt	Proteinuria (R strip)	Urine Microscopy	S. haematobium	
150	Gundersen 1996	91	1996		Malawi	Proteinuria (R strip)	Urine Microscopy	S. haematobium	
151	Hammad 1997	92	1997		Egypt	Proteinuria (R strip)	Urine Microscopy	S. haematobium	
152	Hammam 2000a	93	2000		Egypt	Proteinuria (R strip)	Urine Microscopy	S. haematobium	
153	Hammam 2000b	94	2000		Egypt	Proteinuria (R strip)	Urine Microscopy	S. haematobium	
154	Kassim 1989	95	1989		Nigeria	Proteinuria (R strip)	Urine Microscopy	S. haematobium	
155	Kiliku 1991	96	1991		Kenya	Proteinuria (R strip)	Urine Microscopy	S. haematobium	
156	King 1988a	97	1988		Kenya	Proteinuria (R strip)	Urine Microscopy	S. haematobium	
157	Kitange 1993	37	1993		Tanzania	Proteinuria (R strip)	Urine Microscopy	S. haematobium	
158	Mahmoud 2021	60	2021			Proteinuria (R strip)	Urine Microscopy	S. haematobium	
159	Mott 1985 Ghana	39	1985		Ghana	Proteinuria (R strip)	Urine Microscopy	S. haematobium	
160	Mott 1985 Zambia	39	1985		Zambia	Proteinuria (R strip)	Urine Microscopy	S. haematobium	
161	Murare 1987	40	1987		Zimbabwe	Proteinuria (R strip)	Urine Microscopy	S. haematobium	
162	Ndamukong 2001	98	2001		Cameroon	Proteinuria (R strip)	Urine Microscopy	S. haematobium	
163	Ng'andu 1988	99	1988		Zambia	Proteinuria (R strip)	Urine Microscopy	S. haematobium	
164	Nwaorgu 1992	100	1992		Nigeria	Proteinuria (R strip)	Urine Microscopy	S. haematobium	
165	Ofori 1986	101	1986		Ghana	Proteinuria (R strip)	Urine Microscopy	S. haematobium	
166	Okeke 2014 (LPA)	102	2014		Nigeria	Proteinuria (R strip)	Urine Microscopy	S. haematobium	
167	Okeke 2014 (MPA)	102	2014		Nigeria	Proteinuria (R strip)	Urine Microscopy	S. haematobium	
168	Onayade 1996	103	1996		Nigeria	Proteinuria (R strip)	Urine Microscopy	S. haematobium	
169	Poggensee 2000 (HPA)	104	2000		Tanzania	Proteinuria (R strip)	Urine Microscopy	S. haematobium	
170	Poggensee 2000 (LPA)	104	2000		Tanzania	Proteinuria (R strip)	Urine Microscopy	S. haematobium	
171	Pugh 1980	105	1980		Nigeria	Proteinuria (R strip)	Urine Microscopy	S. haematobium	
172	Rasendramino 1998	106	1998		Madagascar	Proteinuria (R strip)	Urine Microscopy	S. haematobium	
173	Sarda 1986	107	1986		Tanzania	Proteinuria (R strip)	Urine Microscopy	S. haematobium	
174	Sellin 1982	108	1982		Burkina Faso	Proteinuria (R strip)	Urine Microscopy	S. haematobium	
175	Stephenson 1984	109	1984		Kenya	Proteinuria (R strip)	Urine Microscopy	S. haematobium	
176	Tanner 1983 (Liberia)	110	1983		Liberia	Proteinuria (R strip)	Urine Microscopy	S. haematobium	
177	Tanner 1983 (Tanzania)	110	1983		Tanzania	Proteinuria (R strip)	Urine Microscopy	S. haematobium	
178	Traore 1998	111	1998		Mali	Proteinuria (R strip)	Urine Microscopy	S. haematobium	
179	Ugbomoiko 2009a	112	2009		Nigeria	Proteinuria (R strip)	Urine Microscopy	S. haematobium	
180	Ugbomoiko 2009a	112	2009		Nigeria	Proteinuria (R strip)	Urine Microscopy	S. haematobium	
181	Ugbomoiko 2009b	113	2009		Nigeria	Proteinuria (R strip)	Urine Microscopy	S. haematobium	
182	Verle, 1994	114	1994		Senegal	Proteinuria (R strip)	Urine Microscopy	S. haematobium	
183	Wilkins 1979	115	1979		Gambia	Proteinuria (R strip)	Urine Microscopy	S. haematobium	
184	Abdel-Wahab 1992	45	1992		Egypt	Leukocyturia	Urine Microscopy	S. haematobium	
185	Gundersen 1996	91	1996		Malawi	Leukocyturia	Urine Microscopy	S. haematobium	
186	Poggensee 2000 (HPA)	104	2000		Tanzania	Leukocyturia	Urine Microscopy	S. haematobium	
187	Poggensee 2000 (LPA)	104	2000		Tanzania	Leukocyturia	Urine Microscopy	S. haematobium	
188	Rasendramino 1998	106	1998		Madagascar	Leukocyturia	Urine Microscopy	S. haematobium	
189	Mwangi 2018	150	2018		Kenya	LAMP	triplicate KK	S. mansoni	
190	Gandasegui 2018	151	2018		Angola	LAMP	duplicate KK	S. mansoni	
191	Gandasegui 2015	44	2015		Spain	LAMP	Urine Microscopy	S. haematobium	
192	Gandasegui 2018	152	2018		Angola	LAMP	Urine Microscopy	S. haematobium	
193	Bayoumi 2016	153	2016			LAMP	Urine Microscopy	S. haematobium	
194	Zhang 2020	57	2020		Zambia	IHA	Urine Microscopy	S. haematobium	
195	Coulibaly 2022	56	2022		Ivory Coast	SchistoScope	Urine Microscopy	S. haematobium	
196	Bocanegra 2015	46	2015		Angola	Colorimetric test	Urine Microscopy	S. haematobium	

ID	author et al	reference number	the study was publi	year the study was done	study location	diagnostic assay investigated	reference diagnostic assay	parasite specie considered	any other relevant information
197	Espirito-Santo 2015	54	2015		Brazil	COPT vs double KK	double KK	S. mansoni	
198	Anyan 2020	146	2020		Brazil	PCR	KK	S. mansoni	
199	Anyan 2020	146	2020		Brazil	PCR	KK	S. mansoni	
200	Pontes 2003	148	2003			PCR	KK	S. mansoni	
201	Oliveira 2010	147	2010			PCR	KK	S. mansoni	
202	Oliveira 2010	147	2010			PCR	KK	S. mansoni	
203	Al-Shehri 2018	63	2018		Uganda	PCR	CCA1		
204	Lindholz 2018	53	2018		Brazil	Helmintex	duplicate KK	S. mansoni	
205	Magalhaes 2020	61	2020		Brazil	Helmintex vs RT-PCR	PCR		
206	Zhang 2020	57	2020		Zambia	DDIA	Urine Microscopy	S. haematobium	
207	Al-Sherbiny 1999	79	1999		Nigeria	CCA1	Urine Microscopy	S. haematobium	
208	Ayele 2008	80	2008		Ethiopia	CCA1	Urine Microscopy	S. haematobium	
209	Midzi 2009	81	2009		Zimbabwe	CCA1	Urine Microscopy	S. haematobium	
210	Stothard 2009a	82	2009		Tanzania	CCA1	Urine Microscopy	S. haematobium	
211	Sousa 2020	62	2020		Brazil	CCA1 vs Helmintex	Helmintex		
212	Magalhaes 2020	61	2020		Brazil	CCA1	PCR		
213	Al-Sherbiny 1999	79	1999		Nigeria	CAA	Urine Microscopy	S. haematobium	
214	De Clercq 1995	83	1995		Mali	CAA	Urine Microscopy	S. haematobium	
215	El-Morshedy 1996	84	1996		Egypt	CAA	Urine Microscopy	S. haematobium	
216	Ndhlovu 1996	35	1996		Zimbabwe	CAA	Urine Microscopy	S. haematobium	
217	Magalhaes 2020	61	2020		Brazil	RT-PCR	sextuple KK	S. mansoni	
218	Magalhaes 2020	61	2020		Brazil	RT-PCR	duplicate KK	S. mansoni	
219	Schunk 2015	149	2015			RT-PCR	duplicate KK	S. mansoni	
220	Schunk 2015	149	2015			RT-PCR	duplicate KK	S. mansoni	
221	Adriko 2014	49	2014		Uganda	CCA1	KK	S. mansoni	
222	Adriko 2014	49	2014		Uganda	CCA1	KK	S. mansoni	
223	Adriko 2014	49	2014		Uganda	CCA1	KK	S. mansoni	
224	Al-Shehri 2018	63	2018		Uganda	CCA1	KK	S. mansoni	
225	Assaré, 2018	76	2018		Ivory Coast	CCA1	KK	S. mansoni	
226	Bezerra 2020	77	2020		Brazil	CCA1	KK	S. mansoni	
227	Chernet 2017	43	2017		Eritrea	CCA1	KK	S. mansoni	
228	Colley 2013 cameroon	72	2013		Cameroon	CCA1	KK	S. mansoni	
229	Colley 2013 Côte d'ivoire	72	2013		Ivory Coast	CCA1	KK	S. mansoni	
230	Coulibaly 2013	56	2013		Ivory Coast	CCA1	KK	S. mansoni	
231	Dawson 2013	73	2013		Uganda	CCA1	KK	S. mansoni	
232	Dawson 2013	73	2013		Uganda	CCA1	KK	S. mansoni	
233	Elbasheir 2020	33	2020		Central Soudan	CCA1	KK	S. mansoni	
234	Fereira 2017	64	2017		Brazil	CCA1	KK	S. mansoni	
235	Fereira 2017	64	2017		Brazil	CCA1	KK	S. mansoni	
236	Fereira 2017	64	2017		Brazil	CCA1	KK	S. mansoni	
237	Fuss 2018	65	2018		Tanzania	CCA1	KK	S. mansoni	
238	Lamberton 2014	78	2014		Uganda	CCA1	KK	S. mansoni	
239	Legesse 2008	66	2008		Ethiopia	CCA1	KK	S. mansoni	
240	Lindholz 2018	53	2018		Brazil	CCA1	KK	S. mansoni	
241	Lodh 2013	51	2013		Zambia	CCA1	KK	S. mansoni	
242	Mazigo 2018	74	2018		Tanzania	CCA1	KK	S. mansoni	
243	Navaratnam 2012	67	2012		Uganda	CCA1	KK	S. mansoni	
244	Polman 1995	68	1995		Senegal	CCA1	KK	S. mansoni	
245	Polman 1995	68	1995		Senegal	CCA1	KK	S. mansoni	

ID	author et al	reference number	the study was publi	year the study was done	study location	diagnostic assay investigated	reference diagnostic assay	parasite specie considered	any other relevant information
246	Shane 2011	75	2011		Kenya	CCA1	KK	S. mansoni	
247	Shane 2011	75	2011		Kenya	CCA1	KK	S. mansoni	
248	Sousa 2020	62	2020		Brazil	CCA1	KK	S. mansoni	
249	Sousa 2020	62	2020		Brazil	CCA1	KK	S. mansoni	
250	Sousa 2020	62	2020		Brazil	CCA1	KK	S. mansoni	
251	Standley 2010	69	2010		Tanzania+Keny	CCA1	KK	S. mansoni	
252	Standley 2010	69	2010		Tanzania+Keny	CCA1	KK	S. mansoni	
253	Tchuem Tchuente 2012	70	2012		Cameroon	CCA1	KK	S. mansoni	
254	Tchuem Tchuente 2012	70	2012		Cameroon	CCA1	KK	S. mansoni	
255	Van Lieshout 1995	71	1995		Surinam	CCA1	KK	S. mansoni	
256	Van Lieshout 1995	71	1995		Surinam	CCA1	KK	S. mansoni	
257	Adriko 2014	49	2014		Uganda	CCA2	KK	S. mansoni	
258	Adriko 2014	49	2014		Uganda	CCA2	KK	S. mansoni	
259	Polman 1995	68	1995		Senegal	CAA	KK	S. mansoni	
260	Van Lieshout 1995	71	1995		Surinam	CAA	KK	S. mansoni	
261	Van Lieshout 1995	71	1995		Surinam	CAA	KK	S. mansoni	
262	Glintz 2010	50	2010		Ivory Coast	FLOTAC	KK	S. mansoni	
263	Glintz 2010	50	2010		Ivory Coast	FLOTAC	KK	S. mansoni	
264	Glintz 2010	50	2010		Ivory Coast	FLOTAC	KK	S. mansoni	
265	Mwangi 2018	150	2018		Kenya	LAMP	KK	S. mansoni	
266	Gandasegui 2018	151	2018		Angola	LAMP	KK	S. mansoni	
267	Shane 2011	75	2011		Kenya	SWAP ELISA	KK	S. mansoni	
268	Grenfell 2013	143	2013		Brazil	SWAP ELISA	KK	S. mansoni	
269	Grenfell 2013	143	2013		Brazil	AWE-SEA ELISA	KK	S. mansoni	
270	Chernet 2017	43	2017		Eritrea	AWE-SEA ELISA	KK	S. mansoni	
271	Senra 2018	55	2018			PCR-ELISA laboratorial platforn	KK	S. mansoni	
272	Senra 2018	55	2018			PCR-ELISA commercial platforn	KK	S. mansoni	
273	Magalhaes 2020	61	2020		Brazil	RT-PCR	KK	S. mansoni	
274	Magalhaes 2020	61	2020		Brazil	RT-PCR	KK	S. mansoni	
275	Schunk 2015	149	2015			RT-PCR	KK	S. mansoni	
276	Schunk 2015	149	2015			RT-PCR	KK	S. mansoni	
277	Fuss 2018	65	2018		Tanzania	RT-PCR	KK	S. mansoni	

Test comparison	author et al	Year of study	TP	FP	TN	FN	Sensitivity	Specificity	REF+=TP+FN	REF-=FP+TN	IND+=TP+FP	IND-=FN+TN	pREF	pIND
CCA1 cassette vs single KK	Sousa 2020	2020	10	69	136	2	0.83333333	0.66341463	12	205	79	138	5.5%	36.4%
CCA1 cassette vs duplicate KK	Adriko 2014	2014	6	42	49	2	0.75	0.53846154	8	91	48	51	8.1%	48.5%
CCA1 cassette vs duplicate KK	Al-Shehri 2018	2018	113	34	110	1	0.99122807	0.76388889	114	144	147	111	44.2%	57.0%
CCA1 cassette vs duplicate KK	Elbasheir 2020	2020	168	46	268	7	0.96	0.85350318	175	314	214	275	35.8%	43.8%
CCA1 cassette vs duplicate KK	Ferreira 2017	2017	11	71	211	7	0.61111111	0.74822695	18	282	82	218	6.0%	27.3%
CCA1 cassette vs duplicate KK	Fuss 2018	2018	249	34	10	4	0.98418972	0.22727273	253	44	283	14	85.2%	95.3%
CCA1 cassette vs duplicate KK	Legesse 2008	2008	60	60	46	18	0.76923077	0.43396226	78	106	120	64	42.4%	65.2%
CCA1 cassette vs duplicate KK	Lindholz 2018	2018	47	283	123	8	0.85454545	0.30295567	55	406	330	131	11.9%	71.6%
CCA1 cassette vs duplicate KK	Lodh 2013	2013	45	8	10	26	0.63380282	0.55555556	71	18	53	36	79.8%	59.6%
CCA1 cassette vs duplicate KK	Navaratnam 2012	2012	149	193	220	34	0.81420765	0.53268765	183	413	342	254	30.7%	57.4%
CCA1 cassette vs duplicate KK	Polman 1995	1995	327	29	9	57	0.8515625	0.23684211	384	38	356	66	91.0%	84.4%
CCA1 cassette vs duplicate KK	Polman 1995	1995	341	24	14	43	0.88802083	0.36842105	384	38	365	57	91.0%	86.5%
CCA1 cassette vs duplicate KK	Sousa 2020	2020	15	64	136	2	0.88235294	0.68	17	200	79	138	7.8%	36.4%
CCA1 cassette vs duplicate KK	Standley 2010	2010	103	17	37	14	0.88034188	0.68518519	117	54	120	51	68.4%	70.2%
CCA1 cassette vs duplicate KK	Standley 2010	2010	116	44	10	1	0.99145299	0.18518519	117	54	160	11	68.4%	93.6%
CCA1 cassette vs duplicate KK	Tchuem Tchuente 2012	2012	11	71	211	7	0.61111111	0.74822695	18	282	82	218	6.0%	27.3%
CCA1 cassette vs duplicate KK	Van Lieshout 1995	1995	21	10	136	37	0.36206897	0.93150685	58	146	31	173	28.4%	15.2%
CCA1 cassette vs duplicate KK	Van Lieshout 1995	1995	36	23	123	22	0.62068966	0.84246575	58	146	59	145	28.4%	28.9%
CCA1 cassette vs quadruple KK	Adriko 2014	2014	8	40	49	3	0.72727273	0.5505618	11	89	48	52	11.0%	48.0%
CCA1 cassette vs quadruple KK	Chernet 2017	2017	21	22	62	2	0.91304348	0.73809524	23	84	43	64	21.5%	40.2%
CCA1 cassette vs quadruple KK	Colley 2013 cameroon	2013	247	208	231	27	0.90145985	0.5261959	274	439	455	258	38.4%	63.8%
CCA1 cassette vs quadruple KK	Colley 2013 C“te d'ivoire	2013	278	42	249	38	0.87974684	0.8556701	316	291	320	287	52.1%	52.7%
CCA1 cassette vs quadruple KK	Coulibaly 2013	2011	18	43	49	6	0.75	0.5326087	24	92	61	55	20.7%	52.6%
CCA1 cassette vs quadruple KK	Dawson 2013	2013	9	11	18	4	0.69230769	0.62068966	13	29	20	22	31.0%	47.6%
CCA1 cassette vs quadruple KK	Dawson 2013	2013	23	8	8	1	0.95833333	0.5	24	16	31	9	60.0%	77.5%
CCA1 cassette vs quadruple KK	Ferreira 2017	2017	11	71	216	10	0.52380952	0.75261324	21	287	82	226	6.8%	26.6%
CCA1 cassette vs quadruple KK	Mazigo 2018	2018	233	132	45	9	0.96280992	0.25423729	242	177	365	54	57.8%	87.1%
CCA1 cassette vs quadruple KK	Shane 2011	2011	231	664	833	35	0.86842105	0.55644623	266	1497	895	868	15.1%	50.8%
CCA1 cassette vs sextuple KK	Adriko 2014	2014	10	38	47	3	0.76923077	0.55294118	13	85	48	50	13.3%	49.0%
CCA1 cassette vs sextuple KK	Assaré, 2018	2018	38	187	449	7	0.84444444	0.70597484	45	636	225	456	6.6%	33.0%
CCA1 cassette vs sextuple KK	Bezerra 2020	2020	46	11	54	16	0.74193548	0.83076923	62	65	57	70	48.8%	44.9%
CCA1 cassette vs sextuple KK	Ferreira 2017	2017	10	72	224	8	0.55555556	0.75675676	18	296	82	232	5.7%	26.1%
CCA1 cassette vs sextuple KK	Lamberton 2014	2014	66	1	3	6	0.91666667	0.75	72	4	67	9	94.7%	88.2%
CCA1 cassette vs sextuple KK	Shane 2011	2011	176	88	129	11	0.94117647	0.59447005	187	217	264	140	46.3%	65.3%
CCA1 cassette vs sextuple KK	Tchuem Tchuente 2012	2012	322	94	150	59	0.84514436	0.6147541	381	244	416	209	61.0%	66.6%
CCA1 cassette vs 16KK	Sousa 2020	2020	25	54	132	6	0.80645161	0.70967742	31	186	79	138	14.3%	36.4%
CCA2 cassette vs double KK	Adriko 2014	2014	5	4	88	3	0.625	0.95652174	8	92	9	91	8.0%	9.0%
CCA2 cassette vs quadruple KK	Adriko 2014	2014	5	4	85	6	0.45454545	0.95505618	11	89	9	91	11.0%	9.0%
CAA vs duplicate KK	Polman 1995	1995	344	24	14	40	0.89583333	0.36842105	384	38	368	54	91.0%	87.2%
CAA vs duplicate KK	Van Lieshout 1995	1995	6	1	145	52	0.10344828	0.99315068	58	146	7	197	28.4%	3.4%
CAA vs duplicate KK	Van Lieshout 1995	1995	27	20	126	31	0.46551724	0.8630137	58	146	47	157	28.4%	23.0%
FLOTAC (fresh) vs triplicate KK	Glinz 2010	2010	51	9	31	21	0.70833333	0.775	72	40	60	52	64.3%	53.6%
FLOTAC (10 days) vs triplicate KK	Glinz 2010	2010	69	12	28	3	0.95833333	0.7	72	40	81	31	64.3%	72.3%
FLOTAC (30 days) vs triplicate KK	Glinz 2010	2010	71	14	26	1	0.98611111	0.65	72	40	85	27	64.3%	75.9%
SmCTF-RDT vs quadruple KK	Coulibaly 2013	2011	18	62	32	6	0.75	0.34042553	24	94	80	38	20.3%	67.8%
SmCTF-RDT vs quadruple KK	Dawson 2013	2013	21	14	2	3	0.875	0.125	24	16	35	5	60.0%	87.5%
SmCTF-RDT vs quadruple KK	Dawson 2013	2013	7	17	12	6	0.53846154	0.4137931	13	29	24	18	31.0%	57.1%
SmCTF-RDT vs quadruple KK	Nausch 2014	2014	53	23	15	0	1	0.39473684	53	38	76	15	58.2%	83.5%
SmCTF-RDT vs Urine Microscopy	Coulibaly 2013	2011	4	74	37	2	0.66666667	0.33333333	6	111	78	39	5.1%	66.7%

Test comparison	author et al	Year of study	TP	FP	TN	FN	Sensitivity	Specificity	REF+=TP+FN	REF-=FP+TN	IND+=TP+FP	IND-=FN+TN	pREF	pIND
Sm DNA PCR vs duplicate KK	Lodh 2013	2013	45	34	10	0	1	0.22727273	45	44	79	10	50.6%	88.8%
SWAP ELISA vs Sextuple KK	Shane 2011	2011	172	126	169	15	0.9197861	0.57288136	187	295	298	184	38.8%	61.8%
SWAP ELISA vs 18 KK	Grenfell 2013	2013	18	2	18	2	0.9	0.9	20	20	20	20	50.0%	50.0%
IgM ELISA vs triplicate KK	De Oliveira 2005	2005	49	2	85	1	0.98	0.97701149	50	87	51	86	36.5%	37.2%
IgG ELISA vs triplicate KK	Barakat 1983	1983	177	25	290	15	0.921875	0.92063492	192	315	202	305	37.9%	39.8%
IgG ELISA vs triplicate KK	De Oliveira 2005	2005	48	1	86	2	0.96	0.98850575	50	87	49	88	36.5%	35.8%
IgG ELISA vs triplicate KK	Eltiro 1992	1992	163	23	10	4	0.9760479	0.3030303	167	33	186	14	83.5%	93.0%
AWE-SEA ELISA vs quadruple KK	Chernet 2017	2017	22	32	52	1	0.95652174	0.61904762	23	84	54	53	21.5%	50.5%
AWE-SEA ELISA vs 18 KK	Grenfell 2013	2013	17	4	16	3	0.85	0.8	20	20	21	19	50.0%	52.5%
IgG SEA-ELISA vs CCA1	Al-Shehri 2018	2018	142	52	59	5	0.96598639	0.53153153	147	111	194	64	57.0%	75.2%
IgG SEA-ELISA vs Urine Microscopy	Bouilhac 1981	1981	42	5	95	12	0.77777778	0.95	54	100	47	107	35.1%	30.5%
IgG SEA-ELISA vs Urine Microscopy	Mahmoud 2021	2021	34	19	232	5	0.87179487	0.92430279	39	251	53	237	13.4%	18.3%
IgG SEA-ELISA vs Urine Microscopy	Song 2018	2018	55	64	27	3	0.94827586	0.2967033	58	91	119	30	38.9%	79.9%
IgG SEA-ELISA vs Urine Microscopy	Stothard 2009a	2009	41	31	73	5	0.89130435	0.70192308	46	104	72	78	30.7%	48.0%
IgG SEA-ELISA vs Urine Microscopy	Uga 1989	1989	36	2	9	3	0.92307692	0.81818182	39	11	38	12	78.0%	76.0%
IMB-based ELISA vs Urine Microscopy	Mahmoud 2021	2021	37	13	238	2	0.94871795	0.94820717	39	251	50	240	13.4%	17.2%
Anti IGg RDT-Sh vs Urine Microscopy	Sheele 2013	2013	38	70	8	44	0.46341463	0.1025641	82	78	108	52	51.3%	67.5%
Haematuria (R strip) vs Urine Microscopy	Abdel-Wahab 1992	1992	80	102	178	62	0.56338028	0.63571429	142	280	182	240	33.6%	43.1%
Haematuria (R strip) vs Urine Microscopy	Abdel-Wahab 2000	2000	502	1032	3388	196	0.71919771	0.76651584	698	4420	1534	3584	13.6%	30.0%
Haematuria (R strip) vs Urine Microscopy	Anosike 2001	2001	240	106	482	345	0.41025641	0.81972789	585	588	346	827	49.9%	29.5%
Haematuria (R strip) vs Urine Microscopy	Aryeetey 2000	2000	1117	335	191	919	0.54862475	0.36311787	2036	526	1452	1110	79.5%	56.7%
Haematuria (R strip) vs Urine Microscopy	Bassiouny 2014	2014	78	34	536	48	0.61904762	0.94035088	126	570	112	584	18.1%	16.1%
Haematuria (R strip) vs Urine Microscopy	Birrie 1995 (HPA)	1995	44	22	135	25	0.63768116	0.85987261	69	157	66	160	30.5%	29.2%
Haematuria (R strip) vs Urine Microscopy	Birrie 1995 (LPA)	1995	4	6	144	1	0.8	0.96	5	150	10	145	3.2%	6.5%
Haematuria (R strip) vs Urine Microscopy	Birrie 1995 (MPA)	1995	20	17	81	6	0.76923077	0.82653061	26	98	37	87	21.0%	29.8%
Haematuria (R strip) vs Urine Microscopy	Bocanegra 2015	2015	749	95	408	31	0.96025641	0.8111332	780	503	844	439	60.8%	65.8%
Haematuria (R strip) vs Urine Microscopy	Bogoch 2012	2012	19	18	243	0	1	0.93103448	19	261	37	243	6.8%	13.2%
Haematuria (R strip) vs Urine Microscopy	Bosompem 1996	1996	83	8	112	26	0.76146789	0.93333333	109	120	91	138	47.6%	39.7%
Haematuria (R strip) vs Urine Microscopy	Bosompem 2004	2004	33	5	51	52	0.38823529	0.91071429	85	56	38	103	60.3%	27.0%
Haematuria (R strip) vs Urine Microscopy	Cooppan 1987	1987	632	21	159	129	0.8304862	0.88333333	761	180	653	288	80.9%	69.4%
Haematuria (R strip) vs Urine Microscopy	Deribew 2022	2022	142	122	906	1	0.99300699	0.88132296	143	1028	264	907	12.2%	22.5%
Haematuria (R strip) vs Urine Microscopy	El-Sayed 1995	1995	9	176	440	12	0.42857143	0.71428571	21	616	185	452	3.3%	29.0%
Haematuria (R strip) vs Urine Microscopy	Eltoum 1992	1992	145	123	123	34	0.81005587	0.5	179	246	268	157	42.1%	63.1%
Haematuria (R strip) vs Urine Microscopy	Fatiregun 2005	2005	49	49	471	23	0.68055556	0.90576923	72	520	98	494	12.2%	16.6%
Haematuria (R strip) vs Urine Microscopy	French 2007	2007	219	45	1671	41	0.84230769	0.97377622	260	1716	264	1712	13.2%	13.4%
Haematuria (R strip) vs Urine Microscopy	Gabr 2000	2000	648	1829	9007	426	0.60335196	0.83121078	1074	10836	2477	9433	9.0%	20.8%
Haematuria (R strip) vs Urine Microscopy	Gandasegui 2018	2018	62	21	64	25	0.71264368	0.75294118	87	85	83	89	50.6%	48.3%
Haematuria (R strip) vs Urine Microscopy	Gundersen 1996	1996	50	158	51	1	0.98039216	0.24401914	51	209	208	52	19.6%	80.0%
Haematuria (R strip) vs Urine Microscopy	Hammad 1997	1997	712	2408	8490	360	0.6641791	0.77904203	1072	10898	3120	8850	9.0%	26.1%
Haematuria (R strip) vs Urine Microscopy	Hammam 2000a	2000	409	2526	9134	257	0.61411411	0.78336192	666	11660	2935	9391	5.4%	23.8%
Haematuria (R strip) vs Urine Microscopy	Hammam 2000b	2000	245	1464	7503	343	0.41666667	0.83673469	588	8967	1709	7846	6.2%	17.9%
Haematuria (R strip) vs Urine Microscopy	Kassim 1989	1989	99	11	791	21	0.825	0.98628429	120	802	110	812	13.0%	11.9%
Haematuria (R strip) vs Urine Microscopy	Kiliku 1991	1991	159	14	159	94	0.6284585	0.91907514	253	173	173	253	59.4%	40.6%
Haematuria (R strip) vs Urine Microscopy	King 1988a	1988	1362	47	741	459	0.74794069	0.94035533	1821	788	1409	1200	69.8%	54.0%
Haematuria (R strip) vs Urine Microscopy	King 1988b	1988	199	38	187	215	0.48067633	0.83111111	414	225	237	402	64.8%	37.1%
Haematuria (R strip) vs Urine Microscopy	Kitange 1993	1993	80	17	153	3	0.96385542	0.9	83	170	97	156	32.8%	38.3%
Haematuria (R strip) vs Urine Microscopy	Knopp 2015	2015	31	18	1125	26	0.54385965	0.98425197	57	1143	49	1151	4.8%	4.1%
Haematuria (R strip) vs Urine Microscopy	Knopp 2018	2018	1861	2695	52660	751	0.71248086	0.95131424	2612	55355	4556	53411	4.5%	7.9%
Haematuria (R strip) vs Urine Microscopy	Kosinski 2011	2011	52	13	120	70	0.42622951	0.90225564	122	133	65	190	47.8%	25.5%

Test comparison	author et al	Year of study	TP	FP	TN	FN	Sensitivity	Specificity	REF+=TP+FN	REF-=FP+TN	IND+=TP+FP	IND-=FN+TN	pREF	pIND
Haematuria (R strip) vs Urine Microscopy	Lengeler 1993	1993	228	117	797	66	0.7755102	0.87199125	294	914	345	863	24.3%	28.6%
Haematuria (R strip) vs Urine Microscopy	Mahmoud 2021	2021	18	44	207	21	0.46153846	0.8247012	39	251	62	228	13.4%	21.4%
Haematuria (R strip) vs Urine Microscopy	Mafe 1997	1997	416	91	359	190	0.68646865	0.79777778	606	450	507	549	57.4%	48.0%
Haematuria (R strip) vs Urine Microscopy	Mafe 2000	2000	134	61	296	38	0.77906977	0.82913165	172	357	195	334	32.5%	36.9%
Haematuria (R strip) vs Urine Microscopy	Magnussen 2001	2001	107	3	27	33	0.76428571	0.9	140	30	110	60	82.4%	64.7%
Haematuria (R strip) vs Urine Microscopy	Mohammed 2022	2022	23	434	16002	0	1	0.97359455	23	16436	457	16002	0.1%	2.8%
Haematuria (R strip) vs Urine Microscopy	Morenikeji 2014	2014	69	11	198	209	0.24820144	0.94736842	278	209	80	407	57.1%	16.4%
Haematuria (R strip) vs Urine Microscopy	Mott 1985 Ghana	1985	267	20	154	121	0.68814433	0.88505747	388	174	287	275	69.0%	51.1%
Haematuria (R strip) vs Urine Microscopy	Mott 1985 Zambia	1985	382	9	191	74	0.8377193	0.955	456	200	391	265	69.5%	59.6%
Haematuria (R strip) vs Urine Microscopy	Mtasiwa 1996	1996	253	18	113	20	0.92673993	0.86259542	273	131	271	133	67.6%	67.1%
Haematuria (R strip) vs Urine Microscopy	Murare 1987	1987	126	12	58	36	0.77777778	0.82857143	162	70	138	94	69.8%	59.5%
Haematuria (R strip) vs Urine Microscopy	N'Goran 1989	1989	160	111	256	19	0.89385475	0.69754768	179	367	271	275	32.8%	49.6%
Haematuria (R strip) vs Urine Microscopy	Ndamukong 2001	2001	169	4	157	17	0.90860215	0.97515528	186	161	173	174	53.6%	49.9%
Haematuria (R strip) vs Urine Microscopy	Nduka 1995	1995	38	3	917	207	0.15510204	0.99673913	245	920	41	1124	21.0%	3.5%
Haematuria (R strip) vs Urine Microscopy	Ndyomugyenyi 2001	2001	194	36	195	58	0.76984127	0.84415584	252	231	230	253	52.2%	47.6%
Haematuria (R strip) vs Urine Microscopy	Ng'andu 1988	1988	130	43	200	39	0.76923077	0.82304527	169	243	173	239	41.0%	42.0%
Haematuria (R strip) vs Urine Microscopy	Ngasala 2020 Mta Dam area	2020	18	80	249	6	0.75	0.75683891	24	329	98	255	6.8%	27.8%
Haematuria (R strip) vs Urine Microscopy	Ngasala 2020 Uwandani Shehia	2020	56	16	76	2	0.96551724	0.82608696	58	92	72	78	38.7%	48.0%
Haematuria (R strip) vs Urine Microscopy	Nwaorgu 1992	1992	427	149	388	53	0.88958333	0.72253259	480	537	576	441	47.2%	56.6%
Haematuria (R strip) vs Urine Microscopy	Ofori 1986	1986	45	0	54	19	0.703125	1	64	54	45	73	54.2%	38.1%
Haematuria (R strip) vs Urine Microscopy	Okeke 2014 (LPA)	2014	11	8	273	4	0.73333333	0.97153025	15	281	19	277	5.1%	6.4%
Haematuria (R strip) vs Urine Microscopy	Okeke 2014 (MPA)	2014	21	17	118	28	0.42857143	0.87407407	49	135	38	146	26.6%	20.7%
Haematuria (R strip) vs Urine Microscopy	Poggensee 2000 (HPA)	2000	44	26	35	23	0.65671642	0.57377049	67	61	70	58	52.3%	54.7%
Haematuria (R strip) vs Urine Microscopy	Poggensee 2000 (LPA)	2000	4	48	120	3	0.57142857	0.71428571	7	168	52	123	4.0%	29.7%
Haematuria (R strip) vs Urine Microscopy	Pugh 1980	1980	415	444	3993	515	0.44623656	0.89993239	930	4437	859	4508	17.3%	16.0%
Haematuria (R strip) vs Urine Microscopy	Rasendramino 1998	1998	352	32	95	68	0.83809524	0.7480315	420	127	384	163	76.8%	70.2%
Haematuria (R strip) vs Urine Microscopy	Robinson 2009	2009	135	222	317	3	0.97826087	0.58812616	138	539	357	320	20.4%	52.7%
Haematuria (R strip) vs Urine Microscopy	Rollinson 2005	2005	125	16	113	26	0.82781457	0.87596899	151	129	141	139	53.9%	50.4%
Haematuria (R strip) vs Urine Microscopy	Sarda 1986	1986	275	54	918	53	0.83841463	0.94444444	328	972	329	971	25.2%	25.3%
Haematuria (R strip) vs Urine Microscopy	Savioli 1990	1990	113	38	305	64	0.63841808	0.88921283	177	343	151	369	34.0%	29.0%
Haematuria (R strip) vs Urine Microscopy	Sellin 1982	1982	463	356	268	75	0.8605948	0.42948718	538	624	819	343	46.3%	70.5%
Haematuria (R strip) vs Urine Microscopy	Shaw 1998	1998	216	105	415	121	0.64094955	0.79807692	337	520	321	536	39.3%	37.5%
Haematuria (R strip) vs Urine Microscopy	Stephenson 1984	1984	150	6	182	21	0.87719298	0.96808511	171	188	156	203	47.6%	43.5%
Haematuria (R strip) vs Urine Microscopy	Stothard 2009b	2009	42	9	14	1	0.97674419	0.60869565	43	23	51	15	65.2%	77.3%
Haematuria (R strip) vs Urine Microscopy	Tanner 1983 (Liberia)	1983	129	10	68	60	0.68253968	0.87179487	189	78	139	128	70.8%	52.1%
Haematuria (R strip) vs Urine Microscopy	Tanner 1983 (Tanzania)	1983	139	42	344	23	0.85802469	0.89119171	162	386	181	367	29.6%	33.0%
Haematuria (R strip) vs Urine Microscopy	Traore 1998	1998	420	74	392	155	0.73043478	0.84120172	575	466	494	547	55.2%	47.5%
Haematuria (R strip) vs Urine Microscopy	Ugbomoiko 2009a	2009	333	25	189	23	0.93539326	0.88317757	356	214	358	212	62.5%	62.8%
Haematuria (R strip) vs Urine Microscopy	Ugbomoiko 2009a	2009	598	78	630	151	0.79839786	0.88983051	749	708	676	781	51.4%	46.4%
Haematuria (R strip) vs Urine Microscopy	Ugbomoiko 2009b	2009	155	37	183	72	0.68281938	0.83181818	227	220	192	255	50.8%	43.0%
Haematuria (R strip) vs Urine Microscopy	Verl, 1994	1994	205	15	31	101	0.66993464	0.67391304	306	46	220	132	86.9%	62.5%
Haematuria (R strip) vs Urine Microscopy	Wilkins 1979	1979	585	95	771	493	0.54267161	0.89030023	1078	866	680	1264	55.5%	35.0%
Haematuria (R strip) vs Urine Microscopy	Zumstein 1983	1983	134	15	199	48	0.73626374	0.92990654	182	214	149	247	46.0%	37.6%
Proteinuria (R strip) vs Urine Microscopy	Abdel-Wahab 1992	1992	32	31	249	110	0.22535211	0.88928571	142	280	63	359	33.6%	14.9%
Proteinuria (R strip) vs Urine Microscopy	Abdel-Wahab 2000	2000	97	81	4338	602	0.13876967	0.98167006	699	4419	178	4940	13.7%	3.5%
Proteinuria (R strip) vs Urine Microscopy	Aryeetey 2000	2000	610	842	107	1003	0.37817731	0.11275026	1613	949	1452	1110	63.0%	56.7%
Proteinuria (R strip) vs Urine Microscopy	Bogoch 2012	2012	8	53	208	11	0.42105263	0.79693487	19	261	61	219	6.8%	21.8%
Proteinuria (R strip) vs Urine Microscopy	Bosompem 1996	1996	44	10	110	65	0.40366972	0.91666667	109	120	54	175	47.6%	23.6%
Proteinuria (R strip) vs Urine Microscopy	Bosompem 2004	2004	26	17	39	59	0.30588235	0.69642857	85	56	43	98	60.3%	30.5%

Test comparison	author et al	Year of study	TP	FP	TN	FN	Sensitivity	Specificity	REF+=TP+FN	REF-=FP+TN	IND+=TP+FP	IND-=FN+TN	pREF	pIND
Proteinuria (R strip) vs Urine Microscopy	Cooppan 1987	1987	616	112	68	145	0.80946124	0.37777778	761	180	728	213	80.9%	77.4%
Proteinuria (R strip) vs Urine Microscopy	Gabr 2000	2000	185	293	10540	889	0.17225326	0.97295301	1074	10833	478	11429	9.0%	4.0%
Proteinuria (R strip) vs Urine Microscopy	Gundersen 1996	1996	43	163	46	8	0.84313725	0.22009569	51	209	206	54	19.6%	79.2%
Proteinuria (R strip) vs Urine Microscopy	Hammad 1997	1997	662	3081	7817	410	0.61753731	0.71728758	1072	10898	3743	8227	9.0%	31.3%
Proteinuria (R strip) vs Urine Microscopy	Hammam 2000a	2000	297	1174	10487	369	0.44594595	0.89932253	666	11661	1471	10856	5.4%	11.9%
Proteinuria (R strip) vs Urine Microscopy	Hammam 2000b	2000	155	605	8362	433	0.26360544	0.93253039	588	8967	760	8795	6.2%	8.0%
Proteinuria (R strip) vs Urine Microscopy	Kassim 1989	1989	96	98	704	24	0.8	0.87780549	120	802	194	728	13.0%	21.0%
Proteinuria (R strip) vs Urine Microscopy	Kiliku 1991	1991	197	67	106	56	0.77865613	0.61271676	253	173	264	162	59.4%	62.0%
Proteinuria (R strip) vs Urine Microscopy	King 1988a	1988	1343	118	670	478	0.73750686	0.85025381	1821	788	1461	1148	69.8%	56.0%
Proteinuria (R strip) vs Urine Microscopy	Kitange 1993	1993	27	4	166	56	0.3253012	0.97647059	83	170	31	222	32.8%	12.3%
Proteinuria (R strip) vs Urine Microscopy	Mahmoud 2021	2021	29	129	122	10	0.74358974	0.48605578	39	251	158	132	13.4%	54.5%
Proteinuria (R strip) vs Urine Microscopy	Mott 1985 Ghana	1985	334	99	47	38	0.89784946	0.32191781	372	146	433	85	71.8%	83.6%
Proteinuria (R strip) vs Urine Microscopy	Mott 1985 Zambia	1985	428	25	123	75	0.85089463	0.83108108	503	148	453	198	77.3%	69.6%
Proteinuria (R strip) vs Urine Microscopy	Murare 1987	1987	140	25	45	22	0.86419753	0.64285714	162	70	165	67	69.8%	71.1%
Proteinuria (R strip) vs Urine Microscopy	Ndamukong 2001	2001	155	17	144	31	0.83333333	0.89440994	186	161	172	175	53.6%	49.6%
Proteinuria (R strip) vs Urine Microscopy	Ng'andu 1988	1988	90	58	185	79	0.53254438	0.76131687	169	243	148	264	41.0%	35.9%
Proteinuria (R strip) vs Urine Microscopy	Nwaorgu 1992	1992	537	85	352	43	0.92586207	0.80549199	580	437	622	395	57.0%	61.2%
Proteinuria (R strip) vs Urine Microscopy	Ofori 1986	1986	42	13	41	22	0.65625	0.75925926	64	54	55	63	54.2%	46.6%
Proteinuria (R strip) vs Urine Microscopy	Okeke 2014 (LPA)	2014	8	9	272	7	0.53333333	0.96797153	15	281	17	279	5.1%	5.7%
Proteinuria (R strip) vs Urine Microscopy	Okeke 2014 (MPA)	2014	15	18	117	34	0.30612245	0.86666667	49	135	33	151	26.6%	17.9%
Proteinuria (R strip) vs Urine Microscopy	Onayade 1996	1996	53	1	11	40	0.56989247	0.91666667	93	12	54	51	88.6%	51.4%
Proteinuria (R strip) vs Urine Microscopy	Poggensee 2000 (HPA)	2000	8	6	55	59	0.11940299	0.90163934	67	61	14	114	52.3%	10.9%
Proteinuria (R strip) vs Urine Microscopy	Poggensee 2000 (LPA)	2000	1	14	154	6	0.14285714	0.91666667	7	168	15	160	4.0%	8.6%
Proteinuria (R strip) vs Urine Microscopy	Pugh 1980	1980	508	887	3550	422	0.54623656	0.80009015	930	4437	1395	3972	17.3%	26.0%
Proteinuria (R strip) vs Urine Microscopy	Rasendramino 1998	1998	316	20	107	104	0.75238095	0.84251969	420	127	336	211	76.8%	61.4%
Proteinuria (R strip) vs Urine Microscopy	Sarda 1986	1986	234	173	799	94	0.71341463	0.82201646	328	972	407	893	25.2%	31.3%
Proteinuria (R strip) vs Urine Microscopy	Sellin 1982	1982	376	227	397	162	0.69888476	0.63621795	538	624	603	559	46.3%	51.9%
Proteinuria (R strip) vs Urine Microscopy	Stephenson 1984	1984	113	11	177	58	0.66081871	0.94148936	171	188	124	235	47.6%	34.5%
Proteinuria (R strip) vs Urine Microscopy	Tanner 1983 (Liberia)	1983	108	10	68	81	0.57142857	0.87179487	189	78	118	149	70.8%	44.2%
Proteinuria (R strip) vs Urine Microscopy	Tanner 1983 (Tanzania)	1983	136	68	318	26	0.83950617	0.8238342	162	386	204	344	29.6%	37.2%
Proteinuria (R strip) vs Urine Microscopy	Traore 1998	1998	340	84	382	235	0.59130435	0.81974249	575	466	424	617	55.2%	40.7%
Proteinuria (R strip) vs Urine Microscopy	Ugbomoiko 2009a	2009	207	12	202	149	0.58146067	0.94392523	356	214	219	351	62.5%	38.4%
Proteinuria (R strip) vs Urine Microscopy	Ugbomoiko 2009a	2009	602	9	699	147	0.80373832	0.98728814	749	708	611	846	51.4%	41.9%
Proteinuria (R strip) vs Urine Microscopy	Ugbomoiko 2009b	2009	121	45	175	106	0.53303965	0.79545455	227	220	166	281	50.8%	37.1%
Proteinuria (R strip) vs Urine Microscopy	Verl, 1994	1994	168	21	25	138	0.54901961	0.54347826	306	46	189	163	86.9%	53.7%
Proteinuria (R strip) vs Urine Microscopy	Wilkins 1979	1979	701	251	615	377	0.65027829	0.71016166	1078	866	952	992	55.5%	49.0%
Leukocyturia vs Urine Microscopy	Abdel-Wahab 1992	1992	46	20	260	96	0.32394366	0.92857143	142	280	66	356	33.6%	15.6%
Leukocyturia vs Urine Microscopy	Gundersen 1996	1996	37	160	49	14	0.7254902	0.23444976	51	209	197	63	19.6%	75.8%
Leukocyturia vs Urine Microscopy	Poggensee 2000 (HPA)	2000	38	32	29	29	0.56716418	0.47540984	67	61	70	58	52.3%	54.7%
Leukocyturia vs Urine Microscopy	Poggensee 2000 (LPA)	2000	4	92	76	3	0.57142857	0.45238095	7	168	96	79	4.0%	54.9%
Leukocyturia vs Urine Microscopy	Rasendramino 1998	1998	238	30	97	182	0.56666667	0.76377953	420	127	268	279	76.8%	49.0%
LAMP vs triplicate KK	Mwangi 2018	2018	171	0	207	5	0.97159091	1	176	207	171	212	46.0%	44.6%
LAMP vs duplicate KK	Gandasegui 2018	2018	12	37	112	1	0.92307692	0.75167785	13	149	49	113	8.0%	30.2%
LAMP vs Urine Microscopy	Gandasegui 2015	2015	18	8	61	7	0.72	0.88405797	25	69	26	68	26.6%	27.7%
LAMP vs Urine Microscopy	Gandasegui 2018	2018	75	52	33	12	0.86206897	0.38823529	87	85	127	45	50.6%	73.8%
LAMP vs Urine Microscopy	Bayoumi 2016	2016	31	14	24	0	1	0.63157895	31	38	45	24	44.9%	65.2%
IHA vs Urine Microscopy	Zhang 2020	2020	66	16	41	23	0.74157303	0.71929825	89	57	82	64	61.0%	56.2%
SchistoScope vs Urine Microscopy	Coulibaly 2022	2022	30	9	126	5	0.85714286	0.93333333	35	135	39	131	20.6%	22.9%
Colorimetric test vs Urine Microscopy	Bocanegra 2015	2015	415	124	364	376	0.52465234	0.74590164	791	488	539	740	61.8%	42.1%

Test comparison	author et al	Year of study	TP	FP	TN	FN	Sensitivity	Specificity	REF+=TP+FN	REF-=FP+TN	IND+=TP+FP	IND-=FN+TN	pREF	pIND
COPT vs double KK	Espirito-Santo 2015	2015	4	25	542	1	0.8	0.95590829	5	567	29	543	0.9%	5.1%
PCR vs KK	Anyan 2020	2020	45	8	38	0	1	0.82608696	45	46	53	38	49.5%	58.2%
PCR vs KK	Anyan 2020	2020	21	58	84	0	1	0.5915493	21	142	79	84	12.9%	48.5%
PCR vs KK	Pontes 2003	2003	58	16	118	2	0.96666667	0.88059701	60	134	74	120	30.9%	38.1%
PCR vs KK	Oliveira 2010	2010	25	7	61	9	0.73529412	0.89705882	34	68	32	70	33.3%	31.4%
PCR vs KK	Oliveira 2010	2010	28	12	56	6	0.82352941	0.82352941	34	68	40	62	33.3%	39.2%
PCR vs CCA1	Al-Shehri 2018	2018	125	49	62	22	0.85034014	0.55855856	147	111	174	84	57.0%	67.4%
Helmintex vs duplicate KK	Lindholz 2018	2018	54	133	273	1	0.98181818	0.67241379	55	406	187	274	11.9%	40.6%
Helmintex vs RT-PCR	Magalhaes 2020	2020	71	6	66	33	0.68269231	0.91666667	104	72	77	99	59.1%	43.8%
DDIA vs Urine Microscopy	Zhang 2020	2020	53	22	35	36	0.59550562	0.61403509	89	57	75	71	61.0%	51.4%
CCA1 vs Urine Microscopy	Al-Sherbiny 1999	1999	52	90	213	15	0.7761194	0.7029703	67	303	142	228	18.1%	38.4%
CCA1 vs Urine Microscopy	Ayele 2008	2008	51	39	69	47	0.52040816	0.63888889	98	108	90	116	47.6%	43.7%
CCA1 vs Urine Microscopy	Midzi 2009	2009	84	88	70	23	0.78504673	0.44303797	107	158	172	93	40.4%	64.9%
CCA1 vs Urine Microscopy	Stothard 2009a	2009	4	2	102	42	0.08695652	0.98076923	46	104	6	144	30.7%	4.0%
CCA1 vs Helmintex	Sousa 2020	2020	30	46	115	23	0.56603774	0.71428571	53	161	76	138	24.8%	35.5%
CCA1 vs RT-PCR	Magalhaes 2020	2020	70	24	64	38	0.64814815	0.72727273	108	88	94	102	55.1%	48.0%
CAA vs Urine Microscopy	Al-Sherbiny 1999	1999	11	18	285	56	0.1641791	0.94059406	67	303	29	341	18.1%	7.8%
CAA vs Urine Microscopy	De Clercq 1995	1995	199	82	105	55	0.78346457	0.56149733	254	187	281	160	57.6%	63.7%
CAA vs Urine Microscopy	El-Morshedy 1996	1996	117	0	110	30	0.79591837	1	147	110	117	140	57.2%	45.5%
CAA vs Urine Microscopy	Ndhlovu 1996	1996	93	63	20	3	0.96875	0.24096386	96	83	156	23	53.6%	87.2%
RT-PCR vs sextuple KK	Magalhaes 2020	2020	61	42	92	3	0.953125	0.68656716	64	134	103	95	32.3%	52.0%
RT-PCR vs duplicate KK	Magalhaes 2020	2020	41	73	92	0	1	0.55757576	41	165	114	92	19.9%	55.3%
RT-PCR vs duplicate KK	Schunk 2015	2015	32	0	20	3	0.91428571	1	35	20	32	23	63.6%	58.2%
RT-PCR vs duplicate KK	Schunk 2015	2015	33	0	20	2	0.94285714	1	35	20	33	22	63.6%	60.0%
CCA1 vs KK	Adriko 2014	2014	6	42	49	2	0.75	0.53846154	8	91	48	51	8.1%	48.5%
CCA1 vs KK	Adriko 2014	2014	8	40	49	3	0.72727273	0.5505618	11	89	48	52	11.0%	48.0%
CCA1 vs KK	Adriko 2014	2014	10	38	47	3	0.76923077	0.55294118	13	85	48	50	13.3%	49.0%
CCA1 vs KK	Al-Shehri 2018	2018	113	34	110	1	0.99122807	0.76388889	114	144	147	111	44.2%	57.0%
CCA1 vs KK	Assar, 2018	2018	38	187	449	7	0.84444444	0.70597484	45	636	225	456	6.6%	33.0%
CCA1 vs KK	Bezerra 2020	2020	46	11	54	16	0.74193548	0.83076923	62	65	57	70	48.8%	44.9%
CCA1 vs KK	Chernet 2017	2017	21	22	62	2	0.91304348	0.73809524	23	84	43	64	21.5%	40.2%
CCA1 vs KK	Colley 2013 cameroon	2013	247	208	231	27	0.90145985	0.5261959	274	439	455	258	38.4%	63.8%
CCA1 vs KK	Colley 2013 C"te d'ivoire	2013	278	42	249	38	0.87974684	0.8556701	316	291	320	287	52.1%	52.7%
CCA1 vs KK	Coulibaly 2013	2011	18	43	49	6	0.75	0.5326087	24	92	61	55	20.7%	52.6%
CCA1 vs KK	Dawson 2013	2013	23	8	8	1	0.95833333	0.5	24	16	31	9	60.0%	77.5%
CCA1 vs KK	Dawson 2013	2013	9	11	18	4	0.69230769	0.62068966	13	29	20	22	31.0%	47.6%
CCA1 vs KK	Elbasheir 2020	2020	168	46	7	268	0.3853211	0.13207547	436	53	214	275	89.2%	43.8%
CCA1 vs KK	Fereira 2017	2017	11	71	211	7	0.61111111	0.74822695	18	282	82	218	6.0%	27.3%
CCA1 vs KK	Fereira 2017	2017	11	71	216	10	0.52380952	0.75261324	21	287	82	226	6.8%	26.6%
CCA1 vs KK	Fereira 2017	2017	10	72	224	8	0.55555556	0.75675676	18	296	82	232	5.7%	26.1%
CCA1 vs KK	Fuss 2018	2018	249	34	10	4	0.98418972	0.22727273	253	44	283	14	85.2%	95.3%
CCA1 vs KK	Lamberton 2014	2014	66	1	6	3	0.95652174	0.85714286	69	7	67	9	90.8%	88.2%
CCA1 vs KK	Legesse 2008	2008	60	60	46	18	0.76923077	0.43396226	78	106	120	64	42.4%	65.2%
CCA1 vs KK	Lindholz 2018	2018	47	283	123	8	0.85454545	0.30295567	55	406	330	131	11.9%	71.6%
CCA1 vs KK	Lodh 2013	2013	45	8	10	26	0.63380282	0.55555556	71	18	53	36	79.8%	59.6%
CCA1 vs KK	Mazigo 2018	2018	233	132	45	9	0.96280992	0.25423729	242	177	365	54	57.8%	87.1%
CCA1 vs KK	Navaratnam 2012	2012	149	193	220	34	0.81420765	0.53268765	183	413	342	254	30.7%	57.4%
CCA1 vs KK	Polman 1995	1995	327	29	9	57	0.8515625	0.23684211	384	38	356	66	91.0%	84.4%
CCA1 vs KK	Polman 1995	1995	341	24	14	43	0.88802083	0.36842105	384	38	365	57	91.0%	86.5%

Test comparison	author et al	Year of study	TP	FP	TN	FN	Sensitivity	Specificity	REF+=TP+FN	REF-=FP+TN	IND+=TP+FP	IND-=FN+TN	pREF	pIND
CCA1 vs KK	Shane 2011	2011	231	664	833	35	0.86842105	0.55644623	266	1497	895	868	15.1%	50.8%
CCA1 vs KK	Shane 2011	2011	176	88	129	11	0.94117647	0.59447005	187	217	264	140	46.3%	65.3%
CCA1 vs KK	Sousa 2020	2020	10	69	136	2	0.83333333	0.66341463	12	205	79	138	5.5%	36.4%
CCA1 vs KK	Sousa 2020	2020	15	64	136	2	0.88235294	0.68	17	200	79	138	7.8%	36.4%
CCA1 vs KK	Sousa 2020	2020	25	54	132	6	0.80645161	0.70967742	31	186	79	138	14.3%	36.4%
CCA1 vs KK	Standley 2010	2010	116	44	10	1	0.99145299	0.18518519	117	54	160	11	68.4%	93.6%
CCA1 vs KK	Standley 2010	2010	103	17	37	14	0.88034188	0.68518519	117	54	120	51	68.4%	70.2%
CCA1 vs KK	Tchuem Tchuente 2012	2012	322	94	150	59	0.84514436	0.6147541	381	244	416	209	61.0%	66.6%
CCA1 vs KK	Tchuem Tchuente 2012	2012	11	71	211	7	0.61111111	0.74822695	18	282	82	218	6.0%	27.3%
CCA1 vs KK	Van Lieshout 1995	1995	36	23	123	22	0.62068966	0.84246575	58	146	59	145	28.4%	28.9%
CCA1 vs KK	Van Lieshout 1995	1995	21	10	136	37	0.36206897	0.93150685	58	146	31	173	28.4%	15.2%
CCA2 vs KK	Adriko 2014	2014	5	4	88	3	0.625	0.95652174	8	92	9	91	8.0%	9.0%
CCA2 vs KK	Adriko 2014	2014	5	4	85	6	0.45454545	0.95505618	11	89	9	91	11.0%	9.0%
CAA vs KK	Polman 1995	1995	344	24	14	40	0.89583333	0.36842105	384	38	368	54	91.0%	87.2%
CAA vs KK	Van Lieshout 1995	1995	6	1	145	52	0.10344828	0.99315068	58	146	7	197	28.4%	3.4%
CAA vs KK	Van Lieshout 1995	1995	27	20	126	31	0.46551724	0.8630137	58	146	47	157	28.4%	23.0%
FLOTAC vs KK	Glintz 2010	2010	69	12	28	3	0.95833333	0.7	72	40	81	31	64.3%	72.3%
FLOTAC vs KK	Glintz 2010	2010	51	9	31	21	0.70833333	0.775	72	40	60	52	64.3%	53.6%
FLOTAC vs KK	Glintz 2010	2010	71	14	26	1	0.98611111	0.65	72	40	85	27	64.3%	75.9%
LAMP vs KK	Mwangi 2018	2018	171	0	207	5	0.97159091	1	176	207	171	212	46.0%	44.6%
LAMP vs KK	Gandasegui 2018	2018	12	37	112	1	0.92307692	0.75167785	13	149	49	113	8.0%	30.2%
SWAP ELISA vs KK	Shane 2011	2011	172	126	169	15	0.9197861	0.57288136	187	295	298	184	38.8%	61.8%
SWAP ELISA vs KK	Grenfell 2013	2013	18	2	18	2	0.9	0.9	20	20	20	20	50.0%	50.0%
AWE-SEA ELISA vs KK	Grenfell 2013	2013	17	4	16	3	0.85	0.8	20	20	21	19	50.0%	52.5%
AWE-SEA ELISA vs KK	Chernet 2017	2017	22	32	52	1	0.95652174	0.61904762	23	84	54	53	21.5%	50.5%
PCR-ELISA laboratorial platform vs KK	Senra 2018	2018	37	15	153	1	0.97368421	0.91071429	38	168	52	154	18.4%	25.2%
PCR-ELISA commercial platform vs KK	Senra 2018	2018	37	25	143	1	0.97368421	0.85119048	38	168	62	144	18.4%	30.1%
RT-PCR vs KK	Magalhaes 2020	2020	61	42	92	3	0.953125	0.68656716	64	134	103	95	32.3%	52.0%
RT-PCR vs KK	Magalhaes 2020	2020	41	73	92	0	1	0.55757576	41	165	114	92	19.9%	55.3%
RT-PCR vs KK	Schunk 2015	2015	32	0	20	3	0.91428571	1	35	20	32	23	63.6%	58.2%
RT-PCR vs KK	Schunk 2015	2015	33	0	20	2	0.94285714	1	35	20	33	22	63.6%	60.0%
RT-PCR vs KK	Fuss 2018	2018	245	31	13	8	0.96837945	0.29545455	253	44	276	21	85.2%	92.9%
